# Supplementary material for: Patchy nanoparticles by atomic stencilling
Source: Nature. 2025 Oct 15;646(8085):592–600. doi: 10.1038/s41586-025-09605-8 (PMC12527929; doi:10.1038/s41586-025-09605-8)
Supplement: Supplementary file 1 — This file contains Supplementary Notes 1–9, Supplementary Tables 1–15, Supplementary Figs. 1–60 and Supplementary References. [file 41586_2025_9605_MOESM1_ESM.pdf]

nature portfolio

https://doi.org/10.1038/s41586-025-09605-8

Supplementary information

Patchy nanoparticles by atomic stencilling

In the format provided by the authors and unedited

Supplementary information

Patchy Nanoparticles by Atomic Stencilling

Ahyoung Kim[1,14], Chansong Kim[1,14], Tommy Waltmann[2,14], Thi Vo[3], Eun Mi Kim[4], Junseok Kim[4], Yu-Tsun Shao[5], Aaron Michelson[6], John R. Crockett[1], Falon C. Kalutantirige[7], Eric Yang[1], Lehan Yao[1], Chu-Yun Hwang[1], Yugang Zhang[6], Yu-Shen Liu[1], Hyosung An[1,8], Zirui Gao[9], Jiyeon Kim[1], Sohini Mandal[7], David A. Muller[5,10], Kristen A. Fichthorn[4,11]*, Sharon C. Glotzer[2,3,12]*, Qian Chen[1,7,13]*

[1]Department of Materials Science and Engineering, the Grainger College of Engineering, University of Illinois, Urbana, IL 61801, United States

[2]Department of Physics, University of Michigan, Ann Arbor, MI 48109, United States

[3]Department of Chemical Engineering, University of Michigan, Ann Arbor, MI 48109, United States

[4]Department of Chemical Engineering, The Pennsylvania State University, University Park, PA 16802, United States

[5]School of Applied and Engineering Physics, Cornell University, Ithaca, NY 14853, United States

[6]Center for Functional Nanomaterials, Brookhaven National Laboratory, Upton, NY 11973, United States

[7]Department of Chemistry, University of Illinois, Urbana, IL 61801, United States

[8]Department of Petrochemical Materials Engineering, Chonnam National University, Yeosu, 59631, Republic of Korea

[9]National Synchrotron Light Source II, Brookhaven National Laboratory, Upton, NY 11973, United States

[10]Kavli Institute at Cornell for Nanoscale Science, Ithaca, NY 14853, United States

[11]Department of Physics, The Pennsylvania State University, University Park, PA 16802, United States

[12]Biointerfaces Institute, University of Michigan, Ann Arbor, MI 48109, United States

[13]Beckman Institute for Advanced Science and Technology and Materials Research Laboratory, University of Illinois, Urbana, IL 61801, United States

[14]These authors contributed equally.

*To whom the correspondence should be addressed:

fichthorn@psu.edu; sglotzer@umich.edu; qchen20@illinois.edu

Contents

Supplementary Notes 1–9

Supplementary Tables 1–15

Supplementary Figures 1–60

Legends for Supplementary Videos 1–5

References

Table of contents

Supplementary Notes 4
Supplementary Note 1: Synthesis of gold nanoparticles (NPs) 4
1.1. Synthesis of gold nanorods (NRs) 4
1.2. Preparation of universal gold seeds 4
1.3. Synthesis of gold octahedra 5
1.4. Synthesis of gold cuboctahedra 6
1.5. Synthesis of gold rhombic dodecahedra 6
1.6. Synthesis of gold cubes 6
1.7. Synthesis of gold bipyramids 6
Supplementary Note 2: Synthesis of patchy NPs 7
2.1. Synthesis of patchy octahedra 7
2.1.1. Iodide adsorption on octahedra 7
2.1.2. Ligand-mediated polymer grafting on iodide-adsorbed octahedra 8
2.2. Halide ion effect as control experiments 8
2.2.1. Polymer grafting without iodide adsorption on octahedra 8
2.2.2. Polymer grafting with octahedra incubated with other halide ions 9
2.3. Synthesis of patchy bipyramids 9
2.4. Synthesis of patchy cuboctahedra 9
2.5. Synthesis of patchy cubes 10
2.6. Synthesis of patchy large rhombic dodecahedra 10
2.7. Synthesis of patchy small rhombic dodecahedra 11
2.8. Synthesis of patchy palladium nanocubes 11
2.9. Large-scale synthesis of patchy gold octahedra 12
Supplementary Note 3. Transmission electron microscopy (TEM) image-based data analysis for patchy gold NPs 12
3.1. False-coloring on EM images 12
3.2. Local patch thickness analysis (t_{loc}) 12
3.3. Image preprocessing for neural network-based TEM image analysis 13
3.4. Neural network training and prediction 13
3.5. Preparation for neural network-based TEM image analysis 13
3.5.1. Particle isolation function 13
3.5.2. Shape fingerprint extraction 13
3.6. Neural network-based maximum patch thickness (t_m) and coverage fraction (f_{cov}) analysis 13
Supplementary Note 4. Density functional theory (DFT) calculations 14
Supplementary Note 5. Prediction of the iodide-masked region on NPs 14
Supplementary Note 6. Theoretical modeling of polymer grafting 16

Supplementary Note 7. Monte Carlo (MC) simulation of polymer grafting 16
Supplementary Note 8. Molecular Dynamics (MD) simulation of polymer patch grafting 17
8.1. Simulation model for core NPs 17
8.2. Simulation model for polymer chains on iodide-masked NPs 17
8.3. Simulation protocol 18
Supplementary Note 9. MC simulation of patchy NP self-assembly into lattices 18
Supplementary Tables 1–15 19
Supplementary Figures 1–60 35
Legends for Supplementary Videos 96
References 96

Supplementary Notes

Supplementary Note 1: Synthesis of gold nanoparticles (NPs)

1.1. Synthesis of gold nanorods (NRs)

Gold NRs are synthesized by a seeded growth approach following the literature methods.[1,2] The gold NR seed solution is prepared by mixing 125 μL of 10 mM gold(III) chloride trihydrate ($HAuCl_4$) with 5 mL of 100 mM cetyltrimethylammonium bromide (CTAB) solution in a 20 mL vial under mild stirring at 200 rpm. Subsequently, 300 μL of freshly prepared 10 mM ice-cold sodium borohydride ($NaBH_4$) is rapidly injected into the solution under vigorous stirring at 1100 rpm, followed by further stirring the solution at 1100 rpm for 30 s. The resultant gold NR seed solution is left undisturbed for 1 h in a 30 °C water bath before use.

The growth solution is prepared by sequentially mixing 10 mL of 10 mM $HAuCl_4$, 1.8 mL of 10 mM silver nitrate ($AgNO_3$), and 1.14 mL of 100 mM L-ascorbic acid (AA) with 200 mL of 100 mM CTAB solution in a 250 mL Erlenmeyer flask under mild stirring at 200 rpm. Then, 240 μL of the gold NR seed solution is rapidly added to the growth solution under vigorous stirring at 700 rpm. The mixture is stirred further at 700 rpm for 60 s, and left undisturbed for 2 h in a 30 °C water bath during the growth of gold NRs. To remove unreacted reactants, the as-synthesized gold NR solution is equally aliquoted into six 50 mL centrifuge tubes, followed by centrifugation twice at 9,900 × g for 20 min each. After the first round of centrifugation, the supernatant is removed, and the pellets are combined into three centrifuge tubes and dispersed with 40 mL of 50 mM CTAB in each tube. Following the second round of centrifugation, the supernatants are removed, and the pellets are combined into one centrifuge tube and dispersed with 20 mL of 50 mM CTAB. The gold NRs have slight batch-to-batch variations in their size and aspect ratio, while typical UV-Vis spectra of gold NRs have a maximum extinction wavelength λ_{max} of approximately 706 nm.

1.2. Preparation of universal gold seeds

The universal gold seeds are prepared by iterative oxidative dissolution and reductive growth of gold NRs following the literature method.[2] Specifically, 983 μL of 10 mM $HAuCl_4$ is rapidly injected into 142.9 mL of gold NR solution from above in a 250 mL Erlenmeyer flask (**Supplementary Note 1.1**, λ_{max} of 2.0 optical density (OD)). Note that the volume of $HAuCl_4$ solution is adjusted based on the size and aspect ratio of the gold NRs, determined by a 0.5 mL test-scale etching with varying $HAuCl_4$ solution amount using a thermoblock (Eppendorf, ThermoMixer F2.0), following the literature protocol.[2] The reaction mixture is stirred at 200 rpm for 4 h in the 40 °C water bath, where the solution color gradually turns red from dark brown, indicating the etching of gold NRs into pseudo-spherical NPs through a comproportionation reaction.[3] To remove any unreacted reagents, the etched gold NR solution is equally aliquoted into four 50 mL centrifuge tubes, followed by centrifugation twice at 13,800 × g for 30 min each. After the first round of centrifugation, the supernatant is removed, and the pellets are combined into two centrifuge tubes and redispersed with 30 mL of 100 mM cetylpyridinium chloride (CPC) in each tube. Following the second round of centrifugation, the supernatants are removed, and the pellets are combined into one centrifuge tube and redispersed with 10 mL of 100 mM CPC. This solution is noted as Solution I, λ_{max} = 522 nm.

Subsequently, regrowth and etching of Solution I are performed to obtain uniform spherical gold seeds. For regrowth, 10.4 mL of water, 1.62 mL of 10 mM $HAuCl_4$, 27.8 mL of Solution I diluted in 10 mM CPC (1.0 OD at λ_{max} after dilution) are sequentially mixed with 92. 7 mL of 10 mM CPC solution in a 250 mL Erlenmeyer flask under 300 rpm stirring at room temperature (RT). Then, 10.4 mL of 200 mM AA is rapidly added, followed by stirring the solution for 5 s and leaving it undisturbed for 15 min at RT. The color of the solution gradually turns purple from red, as pseudo-spherical gold seeds grow into concave rhombic dodecahedron NPs. To remove unreacted reactants, the solution is equally aliquoted into four 50 mL centrifuge tubes, followed by centrifugation twice at 16,600 × g for 10 min each. After the first round

of centrifugation, the supernatant is removed, and the pellets are combined into two centrifuge tubes and redispersed with 30 mL of 50 mM CTAB in each tube. Following the second round of centrifugation, the supernatants are removed, and the pellets are combined into one centrifuge tube and redispersed with 12 mL of 50 mM CTAB. This solution is noted as Solution II, λ_{max} = 538 nm.

For the second round of etching, 550 µL of 10 mM $HAuCl_4$ is rapidly added to 106.0 mL of Solution II diluted in 100 mM CTAB (1.0 OD at λ_{max} after dilution) under 300 rpm stirring. The reaction solution is stirred at 200 rpm for 4 h in the 40 °C water bath. The color of the solution turns from purple to red as concaved rhombic dodecahedra NPs are etched into spherical NPs. To remove unreacted reactants, the solution is equally aliquoted into four 50 mL centrifuge tubes, followed by centrifugation twice at 16,600 × *g* for 30 min each. After the first round of centrifugation, the supernatants are removed, and the pellets are combined into two centrifuge tubes and redispersed with 30 mL of 100 mM CPC in each tube. Following the second round of centrifugation, the supernatants are removed, and the pellets are combined into one centrifuge tube and diluted with 12 mL of 100 mM CPC (noted as Universal seed I, λ_{max} = 522 nm). Universal seed I is used for gold octahedron and cube syntheses (**Supplementary Fig. 1a**).

Similarly, the seed solutions for rhombic dodecahedra used in patchy NP synthesis and the octahedra incubated with other halide ions as control experiments are obtained, with minor adjustments in reagent concentrations in the etching and regrowth steps as a different batch of gold NR is used. Specifically, in the first round of the etching step, 1138 µL of 10 mM $HAuCl_4$ is used for 162.5 mL of gold NRs in 50 mM CTAB solution to make Solution I (λ_{max} = 524 nm). In the regrowth step, 14.625 mL of water, 2.275 mL of 10 mM $HAuCl_4$, 39 mL of the Solution I (1.0 OD at λ_{max} after dilution), 130 mL of 10 mM CPC, and 14.625 mL of 200 mM AA are used to make Solution II (λ_{max} = 554 nm). In the second round of the etching step, 993 µL of 10 mM $HAuCl_4$ and 185.3 mL of Solution II (1.0 OD at λ_{max} after dilution) are used to make Universal seed II (λ_{max} = 523 nm) (**Supplementary Fig. 1b**). The rest of the procedure and washing steps are the same as above.

Likewise, the universal seed for gold cuboctahedra synthesis is obtained, with minor adjustments in reagent concentrations in the regrowth and etching steps to achieve bigger seeds (Universal seed III, λ_{max} = 525, 1.81 OD at λ_{max}). Specifically, the Universal Seed I undergoes additional regrowth and etching steps. In the regrowth step, 50 mL of Universal Seed I (1.0 OD at λ_{max} after dilution with 100 mM CPC), 166.67 mL of 10 mM CPC, 2.92 mL $HAuCl_4$, and 37.5 mL of 100 mM AA are used. The rest of the procedure and washing steps are the same as above. In the etching step, while keeping every reagent in the same ratio, the volume of 10 mM $HAuCl_4$ is decreased by 10% (**Supplementary Fig. 1c**).

1.3. Synthesis of gold octahedra

Gold octahedra for patchy NP synthesis are synthesized following the literature method with slight modifications.[2] Specifically, 4 mL of 10 mM $HAuCl_4$ and 520 µL of 100 mM AA are sequentially mixed with 200 mL of 100 mM CPC solution in a 250 mL Erlenmeyer flask under 200 rpm stirring at RT. Subsequently, 7.4 mL of Universal Seed I is rapidly added into the reaction mixture under vigorous stirring at 700 rpm, and then left undisturbed for 30 min at RT. To remove unreacted reactants, the solution is equally aliquoted into six 50 mL centrifuge tubes, followed by centrifugation twice at 3,400 × *g* for 15 min each. After the first round of centrifugation, the supernatants are removed, and the pellets are combined into three centrifuge tubes and redispersed with 30 mL of 40 mM CTAB in each tube. Following the second round of centrifugation, the supernatants are removed, and the pellets are combined into one centrifuge tube and redispersed with 30 mL of 40 mM CTAB (λ_{max} = 566 nm, **Supplementary Fig. 2a**).

Gold octahedra used in the control experiments for the halide ion effect (see **Supplementary Note 2.2.2**) are synthesized using the same literature method as above with slight modifications.[2] Specifically, 1 mL of 10 mM $HAuCl_4$ and 130 µL of 100 mM AA are sequentially mixed with 50 mL of 100 mM CPC solution in a 125 mL Erlenmeyer flask under 200 rpm stirring at RT. Subsequently, 1 mL of Universal Seed II is rapidly added into the reaction mixture under vigorous stirring at 700 rpm, and then left undisturbed

for 30 min at RT. To remove unreacted reactants, the solution is equally aliquoted into two 50 mL centrifuge tubes, followed by centrifugation twice at 2,800 × *g* for 15 min each. After the first round of centrifugation, the supernatants are removed, and the pellets are combined into one centrifuge tube and redispersed with 20 mL of 50 mM CTAB (λ_{max} = 582 nm).

1.4. Synthesis of gold cuboctahedra

Gold cuboctahedra are synthesized following the literature method with a slight modification.[2] Specifically, 17.5 mL of 100 mM KBr, 1.75 mL of 10 mM $HAuCl_4$, and 2.625 mL of 100 mM AA are added sequentially to 175 mL of 100 mM CPC in a 250 mL Erlenmeyer flask. Subsequently, 35 mL of Universal seed III is rapidly added into the reaction mixture under vigorous stirring at 700 rpm, and then left undisturbed for 2 h at RT. The remaining steps of washing and redispersion in CTAB are the same as described in **Supplementary Note 1.3**, except that centrifugation is performed at 3,700 × *g* for 15 min (λ_{max} = 543 nm, **Supplementary Fig. 2b**).

1.5. Synthesis of gold rhombic dodecahedra

Gold small rhombic dodecahedra are synthesized following the literature method with a slight modification.[2] Specifically, 7.5 mL of 1 M HCl, 7.5 mL of 10 mM $HAuCl_4$, 390 µL of 10 mM $AgNO_3$, and 900 µL of 100 mM AA are sequentially mixed with 150 mL of 100 mM CPC in a 250 mL Erlenmeyer flask under mild stirring at 200 rpm at RT. Subsequently, 30 mL of the Universal seed II diluted in 100 mM CPC (0.4 OD at λ_{max} after dilution) is rapidly added into the reaction mixture under vigorous stirring at 700 rpm, and then left undisturbed for 5 h in RT. The remaining steps of washing and redispersion in CTAB are the same as described in **Supplementary Note 1.3**, except that centrifugation is performed at 4,300 × *g* for 15 min and redispersed in 20 mL of 50 mM CTAB instead (λ_{max} = 544 nm, **Supplementary Fig. 2c**).

To synthesize the large rhombic dodecahedra, 2 mL of 1 M HCl, 2 mL of 10 mM $HAuCl_4$, 104 µL of 10 mM $AgNO_3$, and 240 µL of 100 mM AA are sequentially mixed with 40 mL of 100 mM CPC in a 125 mL Erlenmeyer flask under mild stirring at 200 rpm. Subsequently, 400 µL of Universal seed II is rapidly added into the reaction mixture under vigorous stirring at 700 rpm, and then left undisturbed for 5 h at RT. The remaining steps of washing and redispersion in CTAB are the same as above, except centrifugation is performed at 1,900 × *g* for 15 min instead (λ_{max} = 561 nm, **Supplementary Fig. 2d**).

1.6. Synthesis of gold cubes

Gold cubes are synthesized following the literature method.[2] 2 mL of 100 mM KBr, 400 µL of 10 mM $HAuCl_4$, and 600 µL of 100 mM AA are sequentially mixed with 20 mL of 100 mM CPC in a 50 mL Erlenmeyer flask under mild stirring at 200 rpm. Subsequently, 640 µL of Universal seed I is rapidly added into the reaction mixture under vigorous stirring at 700 rpm, and then left undisturbed for 1 h at RT. The remaining steps of washing and redispersion in CTAB are the same as described in **Supplementary Note 1.3**, except centrifugation is performed at 3,900 × *g* for 12 min and redispersed in 6 mL of 40 mM CTAB instead (λ_{max} = 571 nm, **Supplementary Fig. 2e**).

1.7. Synthesis of gold bipyramids

Gold bipyramids are synthesized by a seeded growth approach following the literature method.[4] The seed solution for bipyramids is prepared by mixing 250 µL of 10 mM $HAuCl_4$ and 500 µL of 10 mM citric acid with 18.95 mL of water under 500 rpm stirring at RT. Subsequently, 300 µL of 10 mM freshly prepared ice-cold $NaBH_4$ is rapidly injected into the solution, and then stirring for 2 h at RT. This as-prepared bipyramid seed solution is left undisturbed for a week in a dark room before use. The growth solution is prepared by sequentially mixing 7.5 mL of 10 mM $HAuCl_4$, 1.5 mL of 10 mM $AgNO_3$, 3 mL of 1 M HCl, and 1.2 mL of 100 mM AA with 150 mL of 100 mM CTAB solution in a 250 mL Erlenmeyer flask under 400 rpm stirring at a 30 °C water bath. Then, 1.5 mL of the above bipyramid seed solution is rapidly added to the growth solution, and then left undisturbed for 2 h at RT. To remove unreacted reactants,

the as-synthesized solution is equally aliquoted into four 50 mL centrifuge tubes, and then centrifugation twice at 9,900 × *g* for 15 min each. After the first round of centrifugation, the supernatants are removed, and the pellets are combined into three centrifuge tubes and redispersed with 20 mL of 50 mM CTAB in each tube. Following the second round of centrifugation, the supernatants are removed, and the pellets are combined into one 50 mL centrifuge tube and dispersed with 9 mL of water.

The as-prepared gold bipyramids are purified using depletion attraction to remove spherical byproducts.[4] In a typical purification step, 15.2 mL of 500 mM benzyldimethylhexadecylammonium chloride (BDAC) is added to the centrifuge tube containing as-prepared bipyramids under a mild vortex to achieve the final BDAC concentration of 314 mM. Note that the final BDAC concentration varies with the size and aspect ratio of bipyramids. The mixture is left undisturbed for 12 h in the 30 °C water bath. Subsequently, the red supernatant solution containing spherical impurities is removed by pipetting out without disturbing the sediment. The 40 µL sediment is then redispersed by adding 5 mL of water into the tube. This purification process is repeated until the solution reaches the desired level of purity (typically, > 90% bipyramids). We adjust BDAC concentration after each round of purification, depending on the purification results. For example, if the pellet still contains a significant amount of byproduct, the purification is repeated with a 10% lower concentration of BDAC. Conversely, if the bipyramids do not sediment and stay in the supernatant, the purification is repeated with a 10% higher concentration of BDAC. Once the purification is completed, the gold bipyramids are redispersed and stored in 10 mL of 20 mM CTAB (λ_{max} = 729 nm) (**Supplementary Fig. 2f**).

Supplementary Note 2: Synthesis of patchy NPs

2.1. Synthesis of patchy octahedra

2.1.1. Iodide-masking on octahedra

First, we degas 100 mL of deionized (DI) water by purging it in a plastic bottle with N_2 for 1 h to reduce the oxygen content. This degassed water is used throughout the masking process to minimize oxidative etching of gold NPs in the presence of oxygen, which can be described as follows:[5]

$$I_2 + I^- = I_3^- \quad \text{Eq. S1}$$

$$2Au + I_3^- + I^- = 2AuI_2^- \quad \text{Eq. S2}$$

Next, approximately 2.28 mL of gold octahedra solution (**Supplementary Note 1.3**, exact volume varies depending on the concentration of core NP stock solution) is transferred and divided into two 1.5 mL microcentrifuge tubes and centrifuged at 2,400 × *g* for 15 min. After removing the supernatant, the 10 µL pellet from each tube is combined and redispersed in a 15 mL centrifuge tube using 20 mM cetyltrimethylammonium chloride (CTAC) to precisely dilute the solution to achieve a final NP concentration of 0.5 OD at λ_{max} (noted as Stock Solution I). In our typical masking step, the 20 mM CTAC solution is freshly prepared by gently mixing the degassed water and 200 mM CTAC in a 9:1 volume ratio. Subsequently, 6.9 mL of the Stock Solution I is transferred to another 15 mL centrifuge tube for the following iodide incubation procedure.

The iodide solution for the masking step is freshly prepared by mixing 1 mL of 10 mM NaI and 500 µL of 200 mM NaOH with 8.5 mL of degassed water in a 20 mL glass vial. It is important to gently mix this solution by hand swirling to minimize oxygen dissolution into the mixture. Note that the addition of NaOH makes the solution basic, with a pH around 8.0, which can further hinder the oxidative etching of the gold NPs during incubation by shifting the reaction equilibrium, as shown below.[5]

$$2I_2 + 4OH^- = 4I^- + O_2 + 2H_2O \quad \text{Eq. S3}$$

Desired volume of the iodide solution is then added dropwise into Stock solution I in the tube under mild vortex, and the mixture is left undisturbed for 30 min at RT to allow iodide to adsorb on the NP surface

(**Supplementary Tables 2–8**). After 30 min incubation, 2.1 mL of 40 mM CTAB is added to the mixture, followed by two rounds of centrifugation to remove the residual reagents from the gold NPs. After the first round of centrifugation at 5,200 × *g* for 15 min, the pellet is redispersed with 10 mL of 20 mM CTAB, and the solution is centrifuged again at 4,100 × *g* for 15 min. After the second round of centrifugation, the pellet is redispersed with 1 mL of DI water and transferred into 1.5 mL microcentrifuge tubes, followed by centrifugation at 2,200 × *g* for 15 min to reduce the CTAB concentration. Note that centrifugation at this step requires extra caution both in centrifugation time and speed, as extremely low CTAB concentration during centrifugation can easily lead to NP aggregation. To avoid such aggregation, as a general rule of thumb, we perform the final round of centrifugation at above 0.3 mM CTAB during this process.

After removing the supernatant from the centrifuged tube, the 10 µL pellet is redispersed with 1 mL of water. The UV-Vis spectrum is obtained by diluting 10 µL of this NP solution by 10 times in a cuvette with water to determine the dilution factor. Next, we achieve the precise NP concentration of 5.0 OD at λ_{max} and CTAB concentration of 0.07 mM (noted as Stock solution II, the final CTAB concentration can be varied depending on differently shaped NPs. See **Supplementary Tables 2–8**), using additional water and 0.16 mM CTAB (each volume varies depending on the dilution factor calculated from the OD at λ_{max}). In our typical polymer grafting experiments, Stock Solution II provides a sufficient number of NPs to allow about three reaction conditions (batches) of patch grafting, as described in **Supplementary Note 2.1.2**.

2.1.2. Ligand-mediated polymer grafting on iodide-masked octahedra

For the synthesis of patchy octahedra (**Fig. 3c** and **Supplementary Figs. 3**,**4**), 817 µL of dimethylformamide (DMF) is first added to an 8 mL vial. Subsequently, 5 µL of 2-naphthalenethiol (2-NAT) solution (2 mg/mL in DMF, the amount can be varied depending on differently shaped NPs and patch geometry, see **Supplementary Table 2–8** for detailed conditions), 200 µL of Stock solution II, and 80 µL of polystyrene-*b*-polyacrylic acid (PS-*b*-PAA) solution (8 mg/mL in DMF, fixed throughout) are sequentially mixed by dropwise addition under mild vortex. The vial is tightly capped with a Teflon-lined cap, sonicated for 5 s, sealed with parafilm, heated at 110 °C in an oil bath, and left undisturbed for 2 h. The reaction mixture is then cooled down to RT in the oil bath, which typically takes 90 min. The solution is transferred to 1.5 mL microcentrifuge tubes and centrifuged three times at 3,400 × *g*, 1,300 × *g*, and 1,000 × *g* for 15 min each, to separate the residual 2-NAT and PS-*b*-PAA from the patchy gold NPs. After each centrifugation, 1.49 mL of the supernatant is removed, and the 10 µL pellet is redispersed with 1.49 mL of water. Following the third round of centrifugation, the 10 µL pellet is diluted by 90 µL of water as the final patchy octahedra, which is later used for characterizations and self-assembly experiments (see **Methods**). For the patchy octahedra synthesized with biphenyl-4-thiol, the same ligand concentration (2 mg/mL in DMF) is used in place of 2-NAT, with all other conditions kept the same (**Supplementary Fig. 18b**). The as-prepared patchy NPs solution remains colloidally stable for several months and even a long-term storage for up to one year, especially when the patches are sufficiently large and provide strong electrostatic repulsion. The reaction conditions for synthesizing a library of patchy octahedra with varied patch morphologies and dimensions are described in **Supplementary Table 2**.

2.2. Halide ion effect as control experiments

2.2.1. Polymer grafting without iodide adsorption on octahedra

The synthesis of fully coated octahedron NPs (**Supplementary Fig. 3**) is achieved without iodide masking while keeping the remaining steps the same. The gold octahedra (**Supplementary Note 1.3**) are directly used as pristine NPs. Specifically, 6.9 mL of the Stock solution I is transferred to another 15 mL centrifuge tube, and 2.1 mL of 40 mM CTAB is added to the mixture. The remaining washing steps and the ligand-mediated polymer grafting are performed in the same way as described in **Supplementary Note 2.1**. The detailed reaction condition is summarized in **Supplementary Table 2**.

2.2.2. Polymer grafting with octahedra incubated with other halide ions

The synthesis of patchy octahedron NPs using bromide and chloride in comparison with iodide (**Supplementary Fig. 16**) follows the procedure as outlined in **Supplementary Note 2.1**. All other procedures and reaction conditions, including chemical concentrations and washing step, remain unchanged. Specifically, Stock Solution I is mixed with halide ion solutions, prepared by mixing 1 mL of 10 mM halide ions (NaCl, NaBr, or NaI as the three types of halide ions for comparison in different batches), 500 μL of 200 mM NaOH, and 8.5 mL water in 20 mL glass vials. The remaining halide ion incubation step is the same as described in **Supplementary Note 2.1**. After 30 min of incubation, 2.1 mL of 40 mM CTAB is added into each tube, followed by centrifugation at 5,200 × *g* for 15 min to separate the residual reagents from the gold NPs. The 40 μL pellets are then redispersed with 10 mL of 20 mM CTAB, and the solution is centrifuged at 5,200 × *g* for 15 min, followed by removing the supernatant. The 40 μL pellets are redispersed with 1 mL of water and transferred into 1.5 mL microcentrifuge tubes, followed by another centrifugation at 1,200 × *g* for 15 min. After the last round of centrifugation, the supernatants are removed without disturbing the 10 μL pellet, and the concentration of NPs is adjusted to be precisely 5.0 OD at λ_{max} and the CTAB concentration is set to be 0.07 mM, following the method as described in **Supplementary Note 2.1**. The remaining ligand-mediated polymer grafting and washing steps are the same as described in **Supplementary Note 2.1**. The detailed reaction conditions are summarized in **Supplementary Table 3**.

2.3. Synthesis of patchy bipyramids

The synthesis of patchy bipyramids (**Fig. 3f** and **Supplementary Figs. 5**,**6**) is achieved following similar procedures as described in **Supplementary Note 2.1**. Specifically, approximately 540 μL of bipyramid solution in 1.5 mL microcentrifuge tube is centrifuged at 6,900 × *g* for 15 min. The pellet is redispersed in a 15 mL centrifuge tube using 20 mM CTAC to dilute the NP concentration precisely to 0.5 OD at λ_{max}. Subsequently, 7.8 mL of the diluted NP solution is transferred to another 15 mL centrifuge tube. The NP incubation in iodide-masking solution is performed following the procedures in **Supplementary Note 2.1.1** for 30 min, followed by adding 3.2 mL of 40 mM CTAB. The remaining washing and dilution steps follow the same protocol as described in **Supplementary Note 2.1**, with three rounds of centrifugation performed at 11,000 × *g*, 11,000 × *g*, and 6,900 × *g* for 15 min each. After the last round of centrifugation, the supernatant is removed without disturbing the 10 μL pellet, and the concentration of NPs is adjusted to be precisely 5.0 OD at λ_{max} and the CTAB concentration is set to be 0.007 mM, following the method as described in **Supplementary Note 2.1**. The remaining ligand-mediated polymer grafting and washing steps followed the same protocol as described in **Supplementary Note 2.1**, except that three rounds of centrifugation during the washing step are performed at 5,300 × *g* for 15 min, 4,700 × *g* for 7 min, and 4,700 × *g* for 7 min each. Detailed reaction conditions for synthesizing a library of patchy bipyramids with varied patch morphologies and dimensions are summarized in **Supplementary Table 4**.

2.4. Synthesis of patchy cuboctahedra

The synthesis of patchy cuboctahedra (**Fig. 3i–n**, **Extended Data Fig. 6**, **Supplementary Figs. 7–9**) follows similar procedures as described in **Supplementary Note 2.1**. Specifically, approximately 1.2 mL of cuboctahedra solution in 1.5 mL microcentrifuge tube is centrifuged at 2,900 × *g* for 12 min. The 10 μL pellet is redispersed in a 50 mL centrifuge tube using 2 mL of 200 mM CTAB and 18 mL of degassed water to dilute the NP concentration precisely to 0.5 OD at λ_{max}. Subsequently, 6.6 mL of the diluted NP solution is transferred to another 15 mL centrifuge tube. The NP incubation in iodide-masking solution follows the procedures in **Supplementary Note 2.1.1** for 30 min, followed by adding 3 mL of 40 mM CTAB. The remaining washing and dilution steps follow the same protocol as described in **Supplementary Note 2.1**, with three rounds of centrifugation performed at 3,600 × *g*, 3,400 × *g*, and 2,000 × *g* for 15 min each. After the last round of centrifugation, the supernatant is removed without disturbing the 10 μL pellet, and the concentration of NPs is adjusted to be precisely 5.0 OD at λ_{max} and the CTAB concentration is set to be 0.005 mM, following the method as described in **Supplementary Note 2.1**. The remaining ligand-mediated

polymer grafting and washing steps follow the same protocol as described in **Supplementary Note 2.1**, except that three rounds of centrifugation during the washing step are performed at 2,900 × *g* for 25 min, 1,200 × *g* for 20 min, and 900 × *g* for 20 min each. Detailed reaction conditions for synthesizing a library of patchy cuboctahedra with varied patch morphologies and dimensions are summarized in **Supplementary Table 5**.

2.5. Synthesis of patchy cubes

The synthesis of patchy cubes (**Fig. 3o**,**p**, **Supplementary Figs. 10–12**) is achieved following similar procedures as described in **Supplementary Note 2.1**. Specifically, approximately 5.4 mL of cubes solution divided into four 1.5 mL microcentrifuge tubes is centrifuged at 2,900 × *g* for 15 min. The 10 μL pellet from each tube is combined and redispersed in a 15 mL centrifuge tube using 20 mM CTAC to dilute the NP concentration precisely to 0.5 OD at λ_{max}. Subsequently, 7.2 mL of the diluted NP solution is transferred to another 15 mL centrifuge tube. The NP incubation in the iodide solution follows the procedures in **Supplementary Note 2.1.1** for 30 min, followed by adding 2.8 mL of 40 mM CTAB. The remaining washing and dilution steps follow the same protocol as described in **Supplementary Note 2.1**, except that three rounds of centrifugation are performed at 5,200 × *g*, 5,200 × *g*, and 1,200 × *g* for 15 min each. After the last round of centrifugation, the supernatant is removed without disturbing the 10 μL pellet, and the concentration of NPs is adjusted to be precisely 5.0 OD at λ_{max} and the CTAB concentration is set to be 0.01 mM, following the method as described in **Supplementary Note 2.1**. The remaining ligand-mediated polymer grafting and washing steps follow the same protocol as described in **Supplementary Note 2.1**, except that three rounds of centrifugation during the washing step are performed at 1,900 × *g* for 15 min, 500 × *g* for 20 min, and 300 × *g* for 20 min each. For the patchy cubes synthesized with PS-*b*-poly(acrylamide) and PS-*b*-poly(ethylene oxide) (**Supplementary Fig. 18d,f**), the same polymer concentration (8 mg/mL in DMF) is used instead of PS-*b*-PAA, while all other conditions remain the same as described above. Detailed reaction conditions for synthesizing a library of patchy cubes with varied patch morphologies and dimensions are summarized in **Supplementary Table 6**.

2.6. Synthesis of patchy large rhombic dodecahedra

The synthesis of patchy large rhombic dodecahedra (**Fig. 3q**,**r**, **Supplementary Fig. 13**) follows similar procedures as described in **Supplementary Note 2.1**. Specifically, approximately 7.8 mL large rhombic dodecahedra solution divided into six 1.5 mL microcentrifuge tubes is centrifuged at 700 × *g* for 15 min. The 10 μL pellet from each tube is combined and redispersed in a 15 mL centrifuge tube using 20 mM CTAC to dilute the NP concentration precisely to 0.5 OD at λ_{max}. Subsequently, 7.2 mL of the diluted NP solution is transferred to another 15 mL centrifuge tube. The iodide-masking is performed following the procedures in **Supplementary Note 2.1.1** for 30 min, followed by adding 3.8 mL of 40 mM CTAB and centrifugation at 1,000 × *g* for 15 min. After removing the supernatant, the 40 μL pellet is then redispersed with 10 mL of 20 mM CTAB, and the solution is centrifuged at 1,000 × *g* for 15 min. After removing the supernatant, the 40 μL pellet is redispersed with 1 mL of water and transferred into 1.5 mL microcentrifuge tubes, followed by another centrifugation at 1,000 × *g* for 15 min. After the last round of centrifugation, the supernatant is removed without disturbing the 10 μL pellet, and the concentration of NPs has ensured precisely 5.0 OD at $\lambda_{max,}$ and the CTAB concentration is set to be 0.07 mM, following the method as described in **Supplementary Note 2.1**. The remaining ligand-mediated polymer grafting and washing steps follow the same protocol as described in **Supplementary Note 2.1**, except that three rounds of centrifugation during the washing step are performed at 2,900 × *g*, 1,000 × *g*, and 1,500 × *g* for 12 min each. Detailed reaction conditions for synthesizing a library of patchy large rhombic dodecahedra with varied patch morphologies and dimensions are summarized in **Supplementary Table 7**.

2.7. Synthesis of patchy small rhombic dodecahedra

The synthesis of patchy small rhombic dodecahedra (**Extended Data Figs. 7**, **8**, **Supplementary Fig. 14**) follows similar procedures as described in **Supplementary Note 2.1**. Specifically, approximately 2.7 mL of small rhombic dodecahedra solution divided into two 1.5 mL microcentrifuge tubes is centrifuged at 2,900 × *g* for 15 min. The 10 μL pellet from each tube is combined and redispersed in a 15 mL centrifuge tube using 20 mM CTAC to dilute the NP concentration precisely to 0.5 OD at λ_{max}. Subsequently, 7.2 mL of the diluted NP solution is transferred to another 15 mL centrifuge tube. The NP incubation in the iodide solution is performed following the procedures in **Supplementary Note 2.1.1** for 30 min, followed by adding 3.8 mL of 40 mM CTAB. The remaining washing and dilution steps follow the same protocol as described in **Supplementary Note 2.1**, except that three rounds of centrifugation are performed at 7,500 × *g*, 7,500 × *g*, and 2,200 × *g* for 15 min each. After the last round of centrifugation, the supernatant is removed without disturbing the 10 μL pellet, and the concentration of NPs is adjusted to be precisely 5.0 OD at $\lambda_{max,}$ and the CTAB concentration is set to be 0.07 mM, following the method as described in **Supplementary Note 2.1**. The remaining ligand-mediated polymer grafting and washing steps follow the same protocol as described in **Supplementary Note 2.1**, except that three rounds of centrifugation during the washing step are performed at 2,900 × *g* for 12 min, 1,900 × *g* for 7 min, and 1,500 × *g* for 12 min each. Detailed reaction conditions for synthesizing a library of patchy small rhombic dodecahedra with varied patch morphologies and dimensions are summarized in **Supplementary Table 8**.

2.8. Synthesis of patchy palladium nanocubes

Palladium nanocubes are synthesized following a previously reported method with slight modifications.[6] 20 mM palladium(II) chloride (H_2PdCl_4) is first prepared by dissolving 11.78 mg of sodium tetrachloropalladate(II) (Na_2PdCl_4) in 2 mL of 40 mM HCl. The solution is tightly capped and kept undisturbed in a water bath at 30 °C for 1 h. Then, 625 μL of the prepared 20 mM H_2PdCl_4 is added to 6.25 mL of 100 mM CTAB in a 20 mL vial, followed by sequential addition of 1.25 mL of 40 mM potassium iodide (KI), 0.5 mL of 100 mM AA, and 0.75 mL of DI water under shaking at 400 rpm. The vial is capped and quickly transferred to an oil bath, followed by heating at 90 °C for 1 h with stirring at 400 rpm. Afterward, the reaction solution is cooled by immersion in a water bath. To remove unreacted reagents, the as-synthesized palladium cube solution is transferred into a 15 mL centrifuge tube and centrifuged twice at 9,700 × *g* for 10 min each. After the first centrifugation, the supernatant is removed, and the pellet is redispersed in 5 mL of DI water. Following the second round, the supernatant is removed again, and the pellet is dispersed in 2.5 mL of 20 mM CTAB.

Patchy palladium cubes shown in **Supplementary Fig. 60m,n** are synthesized following the same general procedure as described in **Supplementary Note 2.1**, but with adjusted [2-NAT] and without the iodide masking step, as the palladium cubes already have iodide adsorbed on their surfaces from synthesis involving potassium iodide. Specifically, 213 μL of the palladium cube solution above is transferred into a 1.5 mL microcentrifuge tube and centrifuged at 2,900 × *g* for 15 min. The 10 μL pellet is redispersed in a 15 mL centrifuge tube using 20 mM CTAC to reach a total volume of 7.2 mL. The solution is then centrifuged twice. After the first centrifugation at 7,700 × *g* for 15 min, the pellet is redispersed in 10 mL of 20 mM CTAC and centrifuged again at 7,700 × *g* for 15 min. After the second centrifugation, the pellet is redispersed in 1 mL of DI water and transferred into a 1.5 mL microcentrifuge tube, followed by a final centrifugation at 1,000 × *g* for 15 min. After removing the supernatant, the final CTAB concentration and volume are adjusted to 0.07 mM and 610 μL, respectively, using additional DI water and 0.16 mM CTAB solution. For polymer grafting, 815 μL of DMF is first added to an 8 mL vial. Then 5 μL of 2-NAT solution (0.02 mg/mL in DMF), 200 μL of the adjusted palladium cube solution, and 80 μL of PS-*b*-PAA solution (8 mg/mL in DMF) are sequentially mixed by dropwise addition under mild vortex. The vial is tightly capped with a Teflon-lined cap, sonicated for 5 s, sealed with parafilm, heated at 110 °C in an oil bath, and left undisturbed for 2 h. The reaction mixture is then cooled down to RT in the oil bath, which typically takes 90 min. The solution is transferred to 1.5 mL centrifuge tubes and centrifuged three times at 1,900 ×

g, 500 × *g*, and 300 × *g* for 15 min each, to separate the residual 2-NAT and PS-*b*-PAA from the patchy palladium NPs. After the first and second centrifugations, 1.49 mL of the supernatant is removed, and the 10 μL pellet is redispersed with 1.49 mL of water. Following the third round, the 10 μL pellet is diluted with 490 μL of water for long-term storage for TEM observations. Patchy palladium cubes shown in **Supplementary 60o,p** are synthesized the same as above, except that the as-synthesized palladium nanocubes undergo iodide masking step as described in **Supplementary Note 2.1.1** with a final NaI concentration of 6.62 μM.

2.9. Large-scale synthesis of patchy gold octahedra

The large-scale synthesis of patchy gold octahedra is performed following the recipe described in **Supplementary Note 2.1** with the total reaction volumes increased. The experimental results and the synthesis conditions are summarized in **Supplementary Fig. 17** and **Supplementary Table 2**. Take the 20-fold scaled-up synthesis of patchy octahedra as an example, seven duplicates of iodide masked gold octahedra solutions as described in **Supplementary Note 2.1.1** are prepared. After the last step of centrifugation and removal of supernatant, the pellets are combined into one 15 mL centrifuge tube, with the final CTAB concentration and NP concentration adjusted to 0.007 mM and 5.0 OD at λ_{max}, respectively, using additional DI water and 0.16 mM CTAB. For this step, we use the duplicates of small-volume reactions to ensure a good control of the degassing and mixing process. This step can be scaled up potentially as long as good degassing and mixing can be achieved in a big flask.

For the polymer grafting step, direct scaling up by 20 times in a 50 mL round-bottom flask is achieved. Specifically, 16.35 mL of DMF is first added to a 50 mL round-bottom flask. Then, 50 μL of 2-NAT solution (2 mg/mL in DMF), 4 mL of the gold octahedron solution prepared after iodide masking, and 1.6 mL of PS-*b*-PAA solution (8 mg/mL in DMF) are sequentially added into the flask dropwise under sonication for about 20 s to ensure good mixing. After sealing the flask with a glass stopper, wrapping the flask neck with parafilm, and securing it with a flask clip, the flask is heated at 110 °C in an oil bath, and left undisturbed for 2 h. The reaction mixture is then cooled down to RT in the oil bath, which typically takes 90 min. For TEM and SEM characterizations, 1 mL of the reaction mixture was washed by three rounds of centrifugation at 1,900 × *g* for 15 min, 600 × *g* for 15 min, and 300 × *g* for 15 min each, to separate the residual 2-NAT and PS-*b*-PAA from the product patchy octahedra. After the first and second centrifugations, 1.49 mL of the supernatant is removed, and the 10 μL pellet is redispersed with 1.49 mL of water. Following the third round, the 10 μL pellet is diluted with 490 μL of water for long-term storage.

Supplementary Note 3. Transmission electron microscopy (TEM) image-based data analysis for patchy gold NPs

3.1. False-coloring on EM images

False-coloring of patches in the scanning electron microscopy (SEM) and TEM images is performed manually using Adobe Photoshop.

3.2. Local patch thickness analysis (t_{loc})

The local thickness of a polymer patch at a pixel that belongs to the polymer patch (**Fig. 4d**,**h**, **Extended Data Fig. 8a**) is defined as the diameter of the largest circle that incorporates this pixel and stays within the polymer patch.[7] We manually outline the contour of polymer patches on patchy NPs in ImageJ using the built-in "polygon selections" tool to define the area corresponding to the patches. The local thickness map is then obtained using the built-in "Local Thickness" function in ImageJ, as also detailed in our previous work.[7,8]

3.3. Image preprocessing for neural network-based TEM image analysis

TEM images of patchy NPs in dm3 format with a 2048×2048-pixel resolution are rescaled and saved in .tiff files with 8-bit format with a 512×512-pixel resolution using the Python PIL | Image.resize() method. The rescaled pixel resolution matches the input size of the neural network that we use.

3.4. Neural network training and prediction

The training dataset is generated using a range of patchy NP TEM images, including 8 rhombic dodecahedra, 12 octahedra, and 3 bipyramids. To ensure that our trained neural network can be applied to TEM images of NPs with various shapes (i.e., octahedra, rhombic dodecahedra, cubes, cuboctahedra, and bipyramids) and patch morphologies, we select images of patchy NPs synthesized under different [I^-] and [2-NAT], resulting in diverse patch patterns. The "polygon selections" tool in ImageJ is used to manually label the contours of patches, NP, and background region in each TEM image (**Supplementary Fig. 32**), which serve as ground truth. Next, the Keras library in Python is used to augment the manually labeled ground truth and TEM image pairs, where rotation and flipping are randomly combined to generate 1,000 augmented image pairs.[9-11] Next, a randomly initialized U-Net with three output channels, as described in our previous works,[9,10] is trained with an Adam optimizer (learning rate = 10^{-4}) at a validation split of 0.2 (800 images for training and 200 images for validation) in the Keras library on Google Colab. The training is stopped at 40 epochs, which yield training loss, training accuracy, validation loss, and validation accuracy of 0.1692, 98.90%, 0.1702, and 98.40%, respectively. The prediction results are further "trinarized" by assigning each pixel to be polymer patches, NP, or background depending on the highest probability of this pixel among the three channels predicted by the U-Net (**Supplementary Fig. 33**).

3.5. Preparation for neural network-based TEM image analysis

3.5.1. Particle isolation function

After the trinarization of the images as in **Supplementary Note 3.4**, the 'incomplete' NPs that appear at the edge of the TEM images, showing only partial shapes, are excluded from our data analysis (the workflow is described in **Supplementary Fig. 33**). The particle isolation function script, adapted with slight modifications from our previous work,[9] is used to remove these 'incomplete' patchy NPs from trinarized TEM images. Specifically, the 'incomplete' NPs are identified using the MATLAB function "regionprops", which calculates the area of all features in an image. Features with areas that are too small to be recognized as NPs are then removed by converting their pixels to black.

3.5.2. Shape fingerprint extraction

The shape fingerprint t is extracted from the patch contours of each patchy NP to describe the patch shape and position, following our previous report.[9] The centroid of the NP is first determined, and the rays covering from $\theta = -180°$ to $\theta = 179°$ at an interval of 1° are created from the centroid. The distance that a ray travels within the patch region is recorded as a function of the ray angle θ and noted as a shape fingerprint t. Before the shape fingerprint extraction, each patchy NP image is reoriented so that one of the vertices in its projection points upwards. This reorientation process is applied to set a baseline orientation for all other patchy NP TEM images (**Supplementary Figs. 34–36**).

3.6. Neural network-based maximum patch thickness (t_m) and coverage fraction (f_{cov}) analysis

The maximum patch thickness is determined by finding the maxima of the shape fingerprint, following our previous report.[9] The maxima are found using a MATLAB function "findpeaks(a,b)", where input 'a is the shape fingerprint array with θ corresponding t, and 'b' is the numbering in the shape fingerprint. For example, 6 for octahedra and 8 for rhombic dodecahedra are used as 'b'. The patch coverage fraction f_{cov} is calculated by the range of θ where t is non-zero, divided by the total θ range of the NP contour projection, which is 360°.

Supplementary Note 4. Density functional theory (DFT) calculations

All DFT calculations are performed using the Vienna *Ab initio* Simulation Package[12-14] with projector augmented-waves.[15] The Generalized Gradient Approximation by Perdew, Burke, and Ernzerhof is used for the exchange-correlation functional.[16] We choose an energy cutoff of 450 eV as an optimal value for our plane-wave basis set. For the sampling of the first Brillouin zone, Monkhorst-Pack grids are used.[17] We also include the DFT-D3 method of Grimme with the Becke-Jonson damping to describe long-range van der Waals interactions.[18]

A (15 × 15 × 15) k-point mesh is used to calculate the energy of bulk Au. To calculate the total energy of a 2-NAT molecule in the gas phase, a cubic unit cell with a side length of 30 Å and a single k point is used. To investigate the adsorption of iodide and 2-NAT on Au surfaces, we employ a periodic slab consisting of six Au layers. We fix the bottom three Au layers as the bulk positions, with a calculated lattice constant of 4.13 Å, which is in good agreement with other theoretical calculations, including the dispersion correction, and slightly higher than the experimentally observed value of 4.08 Å.[19-22] We include a vacuum spacing of 30 Å and a dipole correction along the z-direction to prevent any interaction between Au periodic slabs. An energy convergence criterion of 10^{-6} eV and a force convergence criterion of 0.01 eV/Å are used for all structural optimizations.

To calculate the binding energy for 2-NAT on Au surfaces, the following equation is used:

$$E_{\text{bind,2NAT}} = [(E_{\text{Au-I slab}} + N_{\text{2NAT}} E_{\text{2NAT}}) - E_{\text{Au-I-2NAT}}]/N_{\text{2NAT}} \qquad \text{Eq. S4}$$

where $E_{\text{Au-I slab}}$ is the energy of an optimized Au-I slab without 2-NAT, N_{2NAT} is the number of 2-NAT on the Au surface, E_{2NAT} is the energy of a single 2-NAT molecule in the gas phase in vacuum, and $E_{\text{Au-I-2NAT}}$ is the energy of the Au slab with the co-adsorption of iodide and 2-NAT. **Supplementary Table 11** shows the results of the convergence tests for the binding energy of 2-NAT on each Au surface with respect to the k-point mesh and cutoff energy. The unit cell sizes used for Au surfaces are also listed in **Supplementary Table 12** with the corresponding optimal k-point meshes.

To identify the chemical potential range for the facet-selectivity of 2-NAT binding to the Au surface in presence of iodide, the surface energy is calculated using the following equation:

$$\gamma_{\text{Au-I-2NAT}} = \frac{E_{\text{Au-I-2NAT}} - N_{\text{Au}} E_{\text{Au}}^{\text{bulk}} - N_{\text{I}} \mu_{\text{I}^-} - N_{\text{2NAT}} \mu_{\text{2NAT}}}{A_{\text{surf}}} - \gamma_{\text{Au}}^{\text{fixed}} \qquad \text{Eq. S5}$$

where $E_{\text{Au-I-2NAT}}$ is the energy of the Au slab with the co-adsorption of iodide and 2-NAT, N_{Au} is the number of Au atoms, $E_{\text{Au}}^{\text{bulk}}$ is the DFT bulk energy per Au atom, N_{I} is the number of absorbed iodide atoms, μ_{I^-} is the chemical potential of solution-phase iodide I^-, N_{2NAT} is the number of adsorbed 2-NAT molecules, μ_{2NAT} is the chemical potential of 2-NAT in solution, and A_{surf} is the surface area of the Au slab. Since adsorbed species are on only one side of the Au slab in our calculations, we subtract the surface energy of a bare Au surface slab $\gamma_{\text{Au}}^{\text{fixed}}$, with atoms fixed at the bulk coordinates.

Supplementary Note 5. Prediction of the iodide-masked region on NPs

The governing principle underlying the formation of facet-selective patches on polyhedral NP surfaces revolves around the pre-adsorption of iodide on NPs. This prevents subsequent polymer chain attachment to the core NP where iodide is present, driving the formation of polymeric patches only at "free" surface sites that are not masked by iodide. As such, it is of interest to develop an *a priori* way to predict the iodide distribution on the NP surfaces. Due to core NP anisotropy, different facets expose different gold face-centered cubic (FCC) planes to the iodide solution. Each plane exhibits different interaction energies with iodide, as confirmed via DFT calculations in **Supplementary Note 4**. From a statistical mechanics perspective, the above problem reduces to one where we have a single species (i.e., iodide) that can attach to a surface with multiple types of interaction sites. Here, we assume that there are three different planes –

{100}, {110}, and {111} – corresponding to three different site types. For ease of notation, we define the {100}, {110}, and {111} planes as types A, B, and C, respectively. Each type has a different interaction energy—ε_A, ε_B, and ε_C —that is guided by DFT calculations. The statistical mechanics problem in the microcanonical ensemble takes the following form. The number of ways to occupy the different interaction sites, W, follows the multinominal distribution as described:

$$W = \prod_i \frac{g_i!}{n_{A_i}!\, n_{B_i}!\, n_{C_i}!\, \left(g_i - \sum_u n_{u_i}\right)!} \qquad \text{Eq. S6}$$

where i is over all the phase space energy levels, g_i is the total number of occupancy sites on each sublevel, u defines the set of site types in the system ($u \in \{A, B, C\}$), and n_{A_i}, n_{B_i}, n_{C_i} are the number of occupied sites for A, B, and C types for a given energy level, respectively. Eq. S6 is then subjected to the following two constraints:

$$E = \sum_i n_{A_i}\varepsilon_A + n_{B_i}\varepsilon_B + n_{C_i}\varepsilon_C \qquad \text{Eq. S7}$$

$$n_i = \sum n_{A_i} + n_{B_I} + n_{C_i} \qquad \text{Eq. S8}$$

where $n_{u,max}$ defines the maximum number of sites of the uth type on the surface. Solving Eq. S6 for the equilibrium A, B, and C occupancy given that the constraints defined by Eqs. S7 and S8 involve the standard Lagrange multiplier approach. It is straightforward, albeit tedious, to perform the requisite derivatives and rearrangement to give a set of equations as follows:

$$\Theta_A = \frac{D_C}{1 + D_A}\left[1 - \frac{D_A}{1 + D_B + D_A D_B}\right]\left\{D_C + \frac{D_A}{1 + D_A}\left[1 - \frac{D_A}{1 + D_B + D_A D_B}\right]\right\}^{-1} \qquad \text{Eq. S9}$$

$$\Theta_B = \frac{D_A D_C}{1 + D_B + D_A D_B}\left\{D_C + \frac{D_A}{1 + D_A}\left[1 - \frac{D_A}{1 + D_B + D_A D_B}\right]\right\}^{-1} \qquad \text{Eq. S10}$$

$$\Theta_C = \frac{D_A}{1 + D_A}\left[1 - \frac{D_A}{1 + D_B + D_A D_B}\right]\left\{D_C + \frac{D_A}{1 + D_A}\left[1 - \frac{D_A}{1 + D_B + D_A D_B}\right]\right\}^{-1} \qquad \text{Eq. S11}$$

where $D_j = e^{\beta(\varepsilon_j - \mu)}$, μ is the chemical potential, $\beta = {}^{1}/_{k_\mathrm{B}T}$, and $\Theta_j = {}^{n_j}/_{g}$ is the equilibrium fractional occupancy of type j sites on the surface. By inspection, it is clear that μ controls the total surface saturation of occupied versus unoccupied sites in the system (i.e., $\sum_u {}^{n_u}/_{g}$). Increasing μ results in more sites being occupied. As a result, μ serves as a free parameter for matching to the iodide concentration employed in experiments. The prediction of the iodide-masked regions from Eqs. S9–S11 are employed in our Monte Carlo grafting simulation to determine the equilibrium polymer patch morphology.

This mapping of iodide-masked regions onto three-dimensional (3D) NP shapes allows us to quantify how surface masking affects the thermodynamics that govern chain conformations, thereby influencing polymer patch formation. The physical description of the microscopic mechanisms responsible for the observed patch morphologies is as follows. Firstly, in the absence of iodide and excess 2-NAT concentration, theory predicts that NPs exhibit negligible chain partitioning and are fully coated by polymers (**Fig. 4b,f**, **Supplementary Fig. 31**), consistent with our experiments. Iodide masking, however, complicates this physical picture. Increasing iodide concentration confines polymer chains to unmasked regions and forces chains to pick between these free regions on the NP, thereby perturbing their equilibrium surface distribution. Phenomenologically, anchoring polymers crowds chains together on the NP surface. Such crowding induces chain extension, creating a high degree of entropic penalty. As such, if given a choice, chain will preferentially occupy high curvature locations (vertices) on the NP surface to minimize

crowding with neighboring chains, thereby maximizing conformational entropy. Through this lens, locations of iodide-masked regions can significantly impact patching behaviors. Face masking prevents chains from occupying low curvature surfaces on the NP, enhancing entropic effects to further drive partitioning to vertices of each respective NP geometry. Conversely, vertex masking or masking of higher curvature facets force chains to occupy sub-optimal facets, counteracting entropic effects. In these cases, chains seek alternative ways to combat additional entropic penalties incurred due to increased local crowding. It is here that enthalpic effects in the form of polymer–polymer attractions play a significant role. Such interactions arise due to the block polymeric nature of the grafts (PS-*b*-PAA). At minimized crowding (high curvature locations), PS-*b*-PAA microphase separation plays a minor role as polymers are dispersed within a good solvent (DMF): that is, chain entropy dominates. However, increased crowding drives chain extension and thus solvent expulsion between neighboring chains. This shift removes solvent screening between the PS and PAA blocks, driving PS blocks to self-aggregate to maximize PS–PS attraction. As such, chain partitioning shifts from being entropy dominated to enthalpy dominated, and polymer distribution favors larger facets out of the non-iodide coated NP surfaces to maximize chain packing. This competition between conformational entropy and enthalpically driven microphase separation determines the free energy of a grafted polymer chain at the various NP surface locations, providing a theoretical basis for understanding all experimentally observed patch morphologies.

Supplementary Note 6. Theoretical modeling of polymer grafting

The PS-*b*-PAA block copolymer system and all relevant solvent conditions in the polymer grafting reaction here are identical to that of our previous work.[23] Built upon the same scaling theory developed in our previous work,[23] we model the relevant polymer interactions governing ligand-mediated polymer grafting. Briefly, due to the presence of chain–chain interaction, the size of the adsorbed polymer chains on the surface of an anisotropic NP core necessarily incorporates the traditional Flory-Huggins parameter χ , as in Eq. S12:

$$R \sim r_o \sigma^{\frac{1}{5}} v_o^{\frac{1}{5}} [1-2\chi]^{\frac{1}{5}} b_l^{\frac{2}{5}} \left[\frac{N}{\Omega}\frac{b_l}{r_o}\right]^{\frac{3}{5}} \qquad \text{Eq. S12}$$

where r_o is the core size, v_o is the excluded volume of Kuhn monomer, b_l is the Kuhn length, N is the degree of polymerization, σ is the grafting density (corresponding to [2-NAT] in the experiment, as the available polymers for grafting onto NPs are determined by [2-NAT])[23] and Ω is a curvature-related shape parameter that defines both the NP core geometry and position on its surface. It is defined as the value of the maximum deviation from a planar surface for each respective core NP geometry. Additionally, we select the location of the maximum curvature on the shape. For convex shapes, the maximal Ω value directly corresponds to the circumsphere-to-insphere diameter ratio. Eq. S12 can be used to compute the free energy of anchoring each chain to the surface via Eq. S13:

$$\beta F \sim \frac{R^2}{N b_l^2} + \frac{v_o N^2 f}{(\Omega R)^3} \qquad \text{Eq. S13}$$

where f is the number of chains anchored to the core. The probability of anchoring chains to the different surface locations on an anisotropic NP is then simply defined as in Eq. S14.

$$P_{graft} \sim e^{-\beta F} \qquad \text{Eq. S14}$$

Supplementary Note 7. Monte Carlo (MC) simulation of polymer grafting

Our grafting simulation protocol involves two steps: 1) determining the surface distribution of iodides on a core NP and 2) placing polymer chains onto the "free" surface sites that are unoccupied by iodide while simultaneously accounting for relevant chain–chain interactions. We start by constructing a grid of points covering the surface of the core NP shapes of interest. This defines the set of locations for

iodide or polymer attachment on NPs. Eqs. S9–S11 defines the equilibrium fraction of iodide coverage at various facets on the surface of the core NP. In other words, the relative ratios between θ_A, θ_B, and θ_C describe the probability of an iodide occupying different facets upon surface adsorption and $\theta_T = \sum_u \theta_i$ defines the fraction of total sites that are occupied by iodide. The placement of iodide at all the various surface sites employs the traditional Metropolis algorithm.[24] The sites occupied by iodide cannot accommodate polymer adsorption, but the remaining open sites are considered "free" sites for polymer attachment. Considerations of polymer attachment begin after the requisite amount of iodides has been placed onto the NP surface. For polymer grafting simulation, we follow the same protocol as described in our previous work.[23] Briefly, we first define an occupancy matrix that logs where all iodides currently sit on the NP surface. Next, the first chain gets attached to the NP surface based on the grafting probability P_{graft} as in Eq. S14 and the Metropolis acceptance criterion. The selected location for this chain attachment is then updated in the occupancy matrix. All "free" surface sites within a correlation length ξ of any polymer-occupied sites gain a favorable chain–chain interaction energy controlled by the Flory-Huggins χ parameter. This entire process of logging and grafting repeats until the targeted grafting density is achieved. For full details, we refer the reader to our previous work.[23]

Supplementary Note 8. Molecular Dynamics (MD) simulation of polymer patch grafting

We conducted MD simulations of octahedra, cuboctahedra, rhombic dodecahedra, and bipyramids grafted with polymer bead chains of defined length at nominal grafting density (grafts per unit area).

8.1. Simulation model for core NPs

To simulate NPs with different shapes in MD, we use the anisotropic Lennard-Jones (ALJ) pairwise interaction potential,[25] as described in Eq. S15:

$$U_{ALJ} = 4\epsilon_s\left[\left(\frac{\sigma_a}{r_a}\right)^{12} - \left(\frac{\sigma_a}{r_a}\right)^{6}\right] + 4\epsilon\left[\left(\frac{\sigma_c}{r_c}\right)^{12} - \left(\frac{\sigma_c}{r_c}\right)^{6}\right] \qquad \text{Eq. S15}$$

The ALJ potential models NPs with distinct shapes by evaluating the canonical Lennard-Jones potential at both the center-to-center distance r_a and at the distance of closest contact between the particle surfaces r_c. The variable $\sigma_c = 0.15\sigma_a$ defines the diameter of the contact interaction, where σ_a is nominal graft density, and $\epsilon_s = (L_1/L_2)\epsilon$ ensures that the NP shape as the interaction distance changes. The variables L_1 and L_2 are defined as follows:

$$L_1 = \left(\frac{\sigma_{ij}}{r_a - M_{ij}}\right)^{12} - \left(\frac{\sigma_{ij}}{r_a - M_{ij}}\right)^{6} \qquad \text{Eq. S16}$$

$$L_2 = \left(\frac{\sigma_a}{r_a}\right)^{12} - \left(\frac{\sigma_a}{r_a}\right)^{6} \qquad \text{Eq. S17}$$

Here, σ_{ij} is the average of the diameters of the two NPs and $M_{ij} = (\omega_i - 0.5\sigma_i) + (\omega_j - 0.5\sigma_j)$, where ω_i is the distance from the center to the point of closest contact on the ith particle. This potential has been used in our previous work[25] to model NP systems similar to those in the current study. For the current work, we choose ALJ interaction strength parameters as follows: $\epsilon_{core-graft} = 1000$, $\epsilon_{core-monomer} = 1$, $\epsilon_{graft-monomer} = 0$, $\epsilon_{monomer-monomer} = 3$. The ALJ diameters for the core NPs are chosen to be their insphere diameter, and 1 for the graft and monomer beads.

8.2. Simulation model for polymer chains on iodide-masked NPs

We model the polymer chains with an attractive bead-spring model, where each monomer is connected to its adjacent monomers through a combined finite extensible non-linear elastic (FENE) + Weeks–Chandler–Andersen (WCA) potential $U_{FENEWCA}$ as in Eq. S18:

$$U_{FENEWCA} = -\frac{1}{2}kr_0{}^2 ln\left[1-\left(\frac{r_a}{r_0}\right)^2\right] + 4\epsilon\left[\left(\frac{\sigma_a}{r_a}\right)^{12} - \left(\frac{\sigma_a}{r_a}\right)^6\right] \quad \text{Eq. S18}$$

Here, $k = 70$, $r_0 = 1.725$, and ϵ and σ_a are chosen consistent with their values for the ALJ pairwise potential. These parameters are consistent with our previous study[25] involving beads of the same diameter, which demonstrate good solvent scaling for polymer chains without attractions.

In our systems, we must modify previous simulation model[25] by making specific sections of the core NP surface inaccessible to the grafted chains to model the iodide adsorption on the NP surface. This is achieved by adding a dense coating of repulsive "mask" particles to areas of the core surface, which are highly masked places by iodide, as predicted by theoretical calculations in **Supplementary Note 5**. The "mask" particles only interact with the graft particles and have solely repulsive interactions, with parameters $\epsilon = 5$ and $\sigma_a = 1.2$.

8.3. Simulation protocol

Simulations are conducted in the NVT ensemble with $k_{\mathrm{B}}T = 1.0$ and a simulation timestep of $dt = 0.0001$. Simulations are first run for 10^7 timesteps without monomer–monomer attractions, allowing the polymer chains to migrate to the non-masked regions of the NP surface and reach their natural equilibrium. After this equilibration period, the attractions between the monomers are set to the values mentioned above, and the simulations run for another 10^7 timesteps.

Supplementary Note 9. MC simulation of patchy NP self-assembly into lattices

We model the steric repulsion between PAAs on the polymer patches with hard-sphere interactions at patch locations on the core NP surface. A hard-sphere radius is chosen to maintain the relative patch size ratio of R_{patch}/R_{core} between simulation and experiment. We model the Au–Au van der Waals attractions between core NPs with a directional, short-range interaction potential at each NP face without patches (**Supplementary Fig. 49**). For the face-patched rhombic dodecahedra, we place the potential at each exposed vertex with $\{111\}$ facet containing no patch. The interaction potential between two points $\vec{r}_i$, $\vec{r}_j$ on NPs with orientations Ω_i, Ω_j is defined by:

$$U(\vec{r}_i, \vec{r}_j, \Omega_i, \Omega_j) = \begin{cases} 0, & \text{if } r_{ij} > \sigma_d \\ -\epsilon f(\Omega_i, \Omega_j), & \text{if } r_{ij} > \sigma_d \end{cases} \quad \text{Eq. S19}$$

where $\epsilon > 0$ is the attraction strength, σ_d is the attraction distance, and

$$f(\Omega_i, \Omega_j) = \begin{cases} 1, & \text{if } \hat{e}_i \cdot \hat{r}_{ij} > \cos(\delta/2) \text{ and } \hat{e}_j \cdot \hat{r}_{ji} > \cos(\delta/2) \\ -\epsilon f(\Omega_i, \Omega_j), & \text{otherwise} \end{cases} \quad \text{Eq. S20}$$

where $\hat{e}_\alpha$ is the director of the interaction on particle α and δ is the interaction opening angle. We use parameters $\epsilon = 3.0$, $\sigma_d = R_{core}$, and $\delta = \pi/4$.

We perform MC simulations of $N = 10{,}000$ particles with the HOOMD-Blue simulation toolkit[26] using the simulation model described above for face-patched rhombic dodecahedra (filling fraction $\phi = 0.45$), face-patched cuboctahedra ($\phi = 0.47$), corner-patched octahedra ($\phi = 0.37$), and face-patched cubes ($\phi = 0.53$ and $\phi = 0.33$). To get final assemblies consisting of a single crystal grain, simulations are annealed from an initial temperature $k_{\mathrm{B}}T = 1.0$ up to a final temperature between $k_{\mathrm{B}}T = 1.2$ and $k_{\mathrm{B}}T = 2.0$, and slowly cooled back down at the end. We run the annealing step for 4×10^6 MC sweeps and cooled the simulation slowly for 3×10^6 MC sweeps.

Supplementary Tables 1–15

Supplementary Table 1. The yield analysis counts the ratio of the number of NPs with the desired patchy pattern to the total number of NPs observed in the TEM and SEM images. As noted, 17 out of the 21 types of patchy NPs with distinctive patterns exhibit synthesis yields higher than 80% (shaded in light blue). Unless otherwise noted, the core particles are made of gold.

Patchy pattern	Yield (%)	Number of NPs examined	Figure index
Octahedron 1*	94.7	57	Fig. 1 “w/ Iodide” 1st row
Octahedron 2	87.0	77	Fig. 1 “w/ Iodide” 2nd row
Octahedron 3	87.0	192	Fig. 1 “w/ Iodide” 3rd row
Octahedron 4**	86.4	110	Fig. 1 “w/ Iodide” 4th row
Bipyramid 1*	96.3	161	Fig. 1 “w/ Iodide” 1st row
Bipyramid 2	76.8	207	Fig. 1 “w/ Iodide” 2nd row
Bipyramid 3	73.6	148	Fig. 1 “w/ Iodide” 3rd row
Bipyramid 4**	90.2	132	Fig. 1 “w/ Iodide” 4th row
Cuboctahedron 1	70.8	120	Fig. 1 “w/ Iodide” 1st & 2nd rows
Cuboctahedron 2	73.5	170	Fig. 1 “w/ Iodide” 3rd & 4th rows
Cuboctahedron 3**	82.0	89	Supplementary Fig. 8 4th column
Cuboctahedron 4**	85.1	67	Supplementary Fig. 15c
Cube 1	93.9	132	Fig. 1 “w/ Iodide” 1st & 2nd rows
Cube 2	96.7	183	Fig. 1 “w/ Iodide” 3rd & 4th rows
Cube 3**	90.1	91	Supplementary Fig. 15d
Rhombic dodecahedron 1	92.9	140	Fig. 1 “w/ Iodide” 1st row
Rhombic dodecahedron 2*	96.8	93	Fig. 1 “w/ Iodide” 2nd row
Rhombic dodecahedron 3	81.8	214	Extended Data 7h–k
Rhombic dodecahedron 4**	85.6	188	Fig. 1 “w/ Iodide” 4th row
Rhombic dodecahedron 5*	80.3	183	Supplementary Fig. 13e 2nd column
Palladium cube	92.5	67	Supplementary Fig. 60m,n

*** Patchy NPs with extended patches.**

**** Patchy NPs with symmetry-broken structures.** Since symmetry-broken patchy NPs can form with varying numbers of patches and locations on NPs, all NPs exhibiting symmetry-broken structures are included in the count.

Supplementary Table 2. Reaction conditions for synthesizing patchy octahedra with various patch patterns.

Masking step*	Polymer grafting step**			Figure index
Final iodide concentration for NP incubation (μM)	Volume of DMF (μL)	Concentration of 2-NAT solution (mg/mL)	Volume of 2-NAT solution (μL)	
0	810	0.2	10	Fig. 1 "w/o Iodide", Fig. 4d, Supplementary Fig. 3
0.17	810	0.2	10	Fig. 1 "w/ Iodide" 1st row, Fig. 4d, Supplementary Fig. 3
0.42	810	0.2	10	Fig. 1 "w/ Iodide" 2nd row, Fig. 4d, Supplementary Fig. 3
0.83	810	0.2	10	Fig. 1 "w/ Iodide" 3rd row, Fig. 3c, Supplementary Figs. 3,4a,b, 47
16.4	810	0.2	10	Supplementary Fig. 3
117.6	810	0.2	10	Fig. 4d, Supplementary Fig. 3
0.83	815	0.2	5	Supplementary Fig. 4a,b
0.83	800	0.2	20	Supplementary Fig. 4a,b
0.83	810	0.2	10	Fig. 1 "w/ Iodide" 4th row, Supplementary Fig. 15a***
0	815	0.002	5	Supplementary Fig. 4c,d
0	815	0.02	5	Supplementary Fig. 4c,d
0	815	0.2	5	Supplementary Fig. 4c,d
0	800	0.2	20	Supplementary Fig. 4c,d
6.62	1630	2	10	Supplementary Fig. 17a 2× scale****
6.62	3260	2	20	Supplementary Fig. 17a 4× scale****
6.62	8150	2	50	Supplementary Fig. 17a 10× scale****
6.62	16350	2	50	Supplementary Fig. 17b,c****
6.62	814	0.5	6	Supplementary Fig. 18b*****

* During the masking step, the volume of Stock Solution I (0.5 OD at λ_{max}) is fixed as 6.9 mL.

** Note that during the polymer grafting step, fixed volumes of 80 μL of PS-*b*-PAA solution (8 mg/mL in DMF) and 200 μL of Stock Solution II (5.0 OD at λ_{max}, 0.07 mM CTAB) are added to the reactor. The polymer grafting reaction is done at 110 °C.

*** The polymer grafting reaction temperature is 90 °C, instead of 110 °C.

**** For scaled-up synthesis, the synthesis protocol is slightly modified. For detailed synthesis conditions, see **Supplementary Note 2.9**.

***** For the patchy cubes synthesized with biphenyl-4-thiol, the same ligand concentration (2 mg/mL in DMF) is used in place of 2-NAT, with all other conditions kept the same as above.

Supplementary Table 3. Reaction conditions for polymer coating on gold octahedra incubated with different halide ions.

Masking step*		Polymer grafting step**			Supplementary figure index
Halide ion type	Final halide ion concentration for NP incubation (μM)	Volume of DMF (μL)	Concentration of 2-NAT solution (mg/mL)	Volume of 2-NAT solution (μL)	
NaI	6.62	814	0.5	6	16c
NaBr	6.62	814	0.5	6	16d
NaCl	6.62	814	0.5	6	16e

* During the masking step, the volume of Stock Solution I (0.5 OD at λ_{max}) is fixed as 8.5 mL.

** During the polymer grafting step, fixed volumes of 80 μL of PS-*b*-PAA solution (8 mg/mL in DMF) and 200 μL of Stock Solution II (5.0 OD at λ_{max}, 0.07 mM CTAB) are added to the reactor. The polymer grafting reaction is done at 110 °C.

Supplementary Table 4. Reaction conditions for synthesizing patchy bipyramids with various patch patterns.

Masking step*	Polymer grafting step**			Figure index
Final iodide concentration for NP incubation (μM)	Volume of DMF (μL)	Concentration of 2-NAT solution (mg/mL)	Volume of 2-NAT solution (μL)	
0	810	0.0002	10	Fig. 1 "w/o Iodide", Supplementary Fig. 6
0.83	810	0.0002	10	Fig. 1 "w/ Iodide" 1st row, Supplementary Fig. 6
1.66	810	0.0002	10	Fig. 1 "w/ Iodide" 2nd row, Supplementary Fig. 6
16.4	810	0.0002	10	Fig. 1 "w/ Iodide" 3rd row, Fig. 3f, Supplementary Figs. 5,6
32.3	810	0.0002	10	Fig. 1 "w/ Iodide" 4th row, Supplementary Fig. 15b***

* During the masking step, the volume of Stock Solution I (0.5 OD at λ_{max}) is fixed as 7.8 mL.

** Note that during the polymer grafting step, fixed volumes of 80 μL of PS-*b*-PAA solution (8 mg/mL in DMF) and 200 μL of Stock Solution II (5.0 OD at λ_{max}, 0.007 mM CTAB) are added to the reactor. The polymer grafting reaction is done at 110 °C.

*** The polymer grafting reaction temperature is 90 °C, instead of 110 °C.

Supplementary Table 5. Reaction conditions for synthesizing patchy cuboctahedra with various patch patterns.

Masking step*	Polymer grafting step**			Figure index
Final iodide concentration for NP incubation (μM)	Volume of DMF (μL)	Concentration of 2-NAT solution (mg/mL)	Volume of 2-NAT solution (μL)	
0.33	745	0.002	75	Supplementary Fig. 9
0.83	745	0.002	75	Fig. 4h, Supplementary Fig. 9
1.66	745	0.002	75	Supplementary Fig. 9
4.98	745	0.002	75	Fig. 1 "w/ Iodide" 3rd & 4th rows, Fig. 3l, Extended Fig. 6g,i, Supplementary Fig. 9
0	810	0.2	10	Fig. 1 "w/o Iodide", Fig. 4h, Supplementary Fig. 8
0.17	810	0.2	10	Supplementary Fig. 8
0.42	810	0.2	10	Fig. 1 "w/ Iodide" 1st & 2nd rows, Figs. 3i, 4h, Extended Fig. 6f,h, Supplementary Fig. 8, 43−46
0.83	810	0.2	10	Supplementary Fig. 8
0	800	0.2	20	Supplementary Fig. 7
0.17	800	0.2	20	Supplementary Fig. 7
0.42	800	0.2	20	Supplementary Fig. 7
69.8	800	0.02	20	Fig. 4h, Supplementary Fig. 15c

* During the masking step, the volume of Stock Solution I (0.5 OD at λ_{max}) is fixed as 7.2 mL.

** Note that during the polymer grafting step, fixed volumes of 80 μL of PS-*b*-PAA solution (8 mg/mL in DMF) and 200 μL of Stock Solution II (5.0 OD at λ_{max}, 0.07 mM CTAB) are added to the reactor. The polymer grafting reaction is done at 110 °C.

Supplementary Table 6. Reaction conditions for synthesizing patchy cubes with various patch patterns.

Masking step*	Polymer grafting step**			
Final iodide concentration for NP incubation (μM)	Volume of DMF (μL)	Concentration of 2-NAT solution (mg/mL)	Volume of 2-NAT solution (μL)	Figure index
0	800	0.2	20	Fig. 1 "w/o Iodide", Supplementary Figs. 10c,d, 11
0.83	800	0.2	20	Supplementary Figs. 11,12
0.83	800	0.2	20	Supplementary Fig. 18d,f***
1.66	800	0.2	20	Supplementary Fig. 11
3.32	800	0.2	20	Fig. 1 "w/ Iodide" 3rd & 4th rows, Fig. 3p, Supplementary Figs. 10g,h, 11
6.62	800	0.2	20	Supplementary Fig. 11
13.2	800	0.2	20	Supplementary Fig. 11
3.32	720	0.2	100	Fig. 1 "w/ Iodide" 1st & 2nd rows, Fig. 3o, Supplementary Fig. 10e,f
0.83	785	0.2	35	Supplementary Fig. 12
0.83	770	0.2	50	Supplementary Fig. 12
0.83	720	0.2	100	Supplementary Fig. 12
26.0	815	0.2	5	Supplementary Fig. 15d

* During the masking step, the volume of Stock Solution I (0.5 OD at λ_{max}) is fixed as 7.2 mL.

** Note that during the polymer grafting step, fixed volumes of 80 μL of PS-*b*-PAA solution (8 mg/mL in DMF) and 200 μL of Stock Solution II (5.0 OD at λ_{max}, 0.01 mM CTAB) are added to the reactor. The polymer grafting reaction is done at 110 °C.

*** For the patchy cubes synthesized with PS-*b*-poly(acrylamide) and PS-*b*-poly(ethylene oxide), the same polymer concentration (8 mg/mL in DMF) is used in place of PS-*b*-PAA, while all other conditions remain identical to those described above.

Supplementary Table 7. Reaction conditions for synthesizing patchy large rhombic dodecahedra with various patch patterns.

Masking step*	Polymer grafting step**			Figure index
Final iodide concentration for NP incubation (μM)	Volume of DMF (μL)	Concentration of 2-NAT solution (mg/mL)	Volume of 2-NAT solution (μL)	
0.50	720	0.002	100	Fig. 1 "w/ Iodide" 3rd row, Fig. 3r, Supplementary Fig. 13c,d
8.26	720	0.2	100	Fig. 1 "w/ Iodide" 1st row, Fig. 3q, Supplementary Fig. 13a,b,e
8.26	720	0.02	100	Supplementary Fig. 13e
8.26	720	0.002	100	Supplementary Fig. 13e

* During the masking step, the volume of Stock Solution I (0.5 OD at λ_{max}) is fixed as 7.2 mL.

** Note that during the polymer grafting step, fixed volumes of 80 μL of PS-*b*-PAA solution (8 mg/mL in DMF) and 200 μL of Stock Solution II (5.0 OD at λ_{max}, 0.007 mM CTAB) are added to the reactor. The polymer grafting reaction is done at 110 °C.

Supplementary Table 8. Reaction conditions for synthesizing patchy small rhombic dodecahedra with various patch patterns.

Masking step*	Polymer grafting step**			
Final iodide concentration for NP incubation (μM)	Volume of DMF (μL)	Concentration of 2-NAT solution (mg/mL)	Volume of 2-NAT solution (μL)	Figure index
0	815	0.002	5.0	Fig. 1 “w/o Iodide”, Extended Fig. 8, Supplementary Fig. 14
0.17	815	0.002	5.0	Fig. 1 “w/ Iodide” 2nd row, Extended Fig. 8, Supplementary Fig. 14
0.67	815	0.002	5.0	Extended Figs. 7,8, Supplementary Figs. 14, 39−42
1.66	815	0.002	5.0	Supplementary Fig. 14
32.3	815	0.002	5.0	Supplementary Fig. 14
62.5	815	0.002	5.0	Extended Fig. 8, Supplementary Fig. 14
117.6	815	0.002	5.0	Fig. 1 “w/ Iodide” 4th row, Supplementary Fig. 15e

* During the masking step, the volume of Stock Solution I (0.5 OD at λ_{max}) is fixed as 7.2 mL.

** Note that during the polymer grafting step, fixed volumes of 80 μL of PS-*b*-PAA solution (8 mg/mL in DMF) and 200 μL of Stock Solution II (5.0 OD at λ_{max}, 0.007 mM CTAB) are added to the reactor. The polymer grafting reaction is done at 110 °C.

Supplementary Table 9. Reaction conditions for synthesizing iodide-masked octahedra for XPS characterizations. For detailed experimental procedures, see **Methods**.

Masking step*
Final iodide concentration for NP incubation (μM)
0
0.17
0.83
13.3

* During the masking step, the volume of Stock Solution I (0.5 OD at λ_{max}) is fixed as 6.9 mL. Note that XPS samples are obtained without ligand-mediated polymer grafting reaction.

Supplementary Table 10. Reaction conditions for synthesizing iodide-masked octahedra coated only with 2-NAT and without PS-*b*-PAA for Raman characterizations of 2-NAT adsorption. For detailed experimental procedures, see **Methods**.

Masking step*	2-NAT adsorption**		
Final iodide concentration for NP incubation (μM)	Volume of DMF (μL)	Concentration of 2-NAT solution (mg/mL)	Volume of 2-NAT solution (μL)
0.42	860	0.2	40
0.83	860	0.2	40
16.4	860	0.2	40

* During the masking step, the volume of Stock Solution I (0.5 OD at λ_{max}) is fixed as 9.5 mL.

** Note that during the 2-NAT grafting step, fixed volumes of 200 μL of Stock Solution II (5.0 OD at λ_{max}, 0.07 mM CTAB) are added to the reactor without PS-*b*-PAA. The reaction is done at 110 °C.

Supplementary Table 11. Convergence test on the molecular binding energy of 2-NAT on gold surfaces with respect to the *k*-point mesh and energy cutoff.

k-point Mesh					
Au (111) – p(2×2)<br>$E_{bind, 2NAT}$ (eV)	(10×10×1)<br>−1.81	(11×11×1)<br>−1.82	(12×12×1)<br>−1.81	(13×13×1)<br>−1.81	(14×14×1)<br>−1.81
Au (100) – (2×2)<br>$E_{bind, 2NAT}$ (eV)	(10×10×1)<br>−2.47	(11×11×1)<br>−2.46	(12×12×1)<br>−2.45	(13×13×1)<br>−2.46	(14×14×1)<br>−2.46
Au (110) – (2×2)<br>$E_{bind, 2NAT}$ (eV)	(6×9×1)<br>−3.17	(7×10×1)<br>−3.19	(8×11×1)<br>−3.18	(9×12×1)<br>−3.18	(10×14×1)<br>−3.19
**Energy cutoff**					
Au (111) – (2×2)<br>$E_{bind, 2NAT}$ (eV)	450 eV<br>−1.81	480 eV<br>−1.82	500 eV<br>−1.82	530 eV<br>−1.81	550 eV<br>−1.81

* Note that all convergence tests are performed within 0.01 eV using single point energy calculations. The shaded boxes are chosen *k*-point mesh after the convergence tests. Centered unit cells are used, unless a unit cell is noted by p (*i.e.,* primitive unit cell).

Supplementary Table 12. Unit cells used for gold surfaces with corresponding *k*-point meshes.

Au(111)					
Unit Cell	p(2×2)	(2×2)	(√3×√3)	(4×3)	(4×4)
k-point Mesh	(10×10×1)	(12×10×1)	(10×10×1)	(4×5×1)	(4×3×1)
Au(100)					
Unit Cell	(2×2)	(2×6)	(4×4)		
k-point Mesh	(11×11×1)	(9×3×1)	(5×5×1)		
Au(110)					
Unit Cell	(2×2)	(2×6)	(4×4)		
k-point Mesh	(8×11×1)	(9×4×1)	(3×4×1)		

* Note that centered unit cells are used, unless a unit cell is noted by p (*i.e.,* primitive unit cell).

Supplementary Table 13. The surface coverages of iodide and 2-NAT (in monolayer, ML) on three different facets, predicted by DFT calculations. The region numbers are depicted in the phase diagram below, which is also shown in **Fig. 2e**. Regions where stencil effect is predicted are shaded in cyan.

	Au(111)		Au(100)		Au(110)	
Region	I^-	2-NAT	I^-	2-NAT	I^-	2-NAT
1	0.33	0	0	0.25	0.5	0
2	0.33	0	0.44	0	0.5	0
3	0.33	0	0	0.33	0	0.5
4	0.33	0	0.5	0	0	0.5
5	0.33	0	0.25	0.25	0	0.5
6	0	0.33	0	0.33	0	0.5
7	0	0.33	0.25	0.25	0	0.5

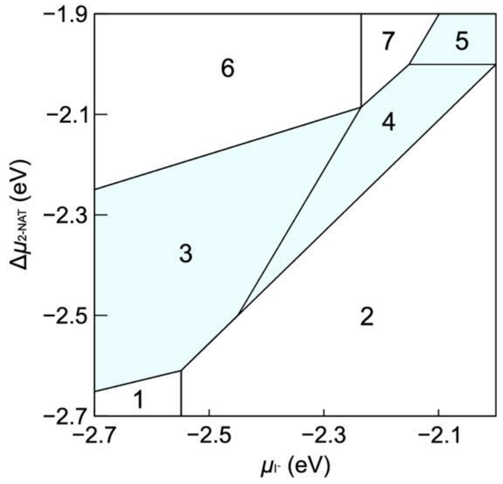

Supplementary Table 14. Interparticle spacings of self-assembled structures prepared with pristine and patchy NPs of different core NP shapes. SEM images of the assembly structures are shown in **Fig. 5** and **Supplementary Figs. 39−48**.

NP shape	Lattice spacing of pristine NP assembly (nm)	Lattice spacing of patchy NP assembly (nm)
Rhombic dodecahedron	54.9 ± 2.2	77.5 ± 6.9
Cuboctahedron	53.0 ± 2.1	73.8 ± 5.2
Octahedron	70.4 ± 2.1	80.2 ± 5.5
Cube	73.6 ± 2.7	86.6 ± 8.1

* A total of 80 nearest NP center-to-center distances are measured from SEM images to determine average interparticle spacing and standard deviation.

Supplementary Table 15. Assembly lattice structures of pristine and patchy NPs of different core NP shapes. SEM images of the assembly structures are shown in **Fig. 5** and **Supplementary Figs. 39−48**.

NP shape	Assembly lattice of pristine NPs	Assembly lattice of patchy NPs
Rhombic dodecahedron	Face-centered cubic (FCC)[27]	Body-centered cubic (BCC)
Cuboctahedron	FCC[28]	BCC
Octahedron	Simple hexagonal[27,28]	BCC
Cube	Simple cubic[27]<br>Rhombic[29]	Body-centered tetragonal (BCT)

The pristine NP assembly structures we experimentally observed are consistent with previous reports cited herein.

Supplementary Figures 1–60

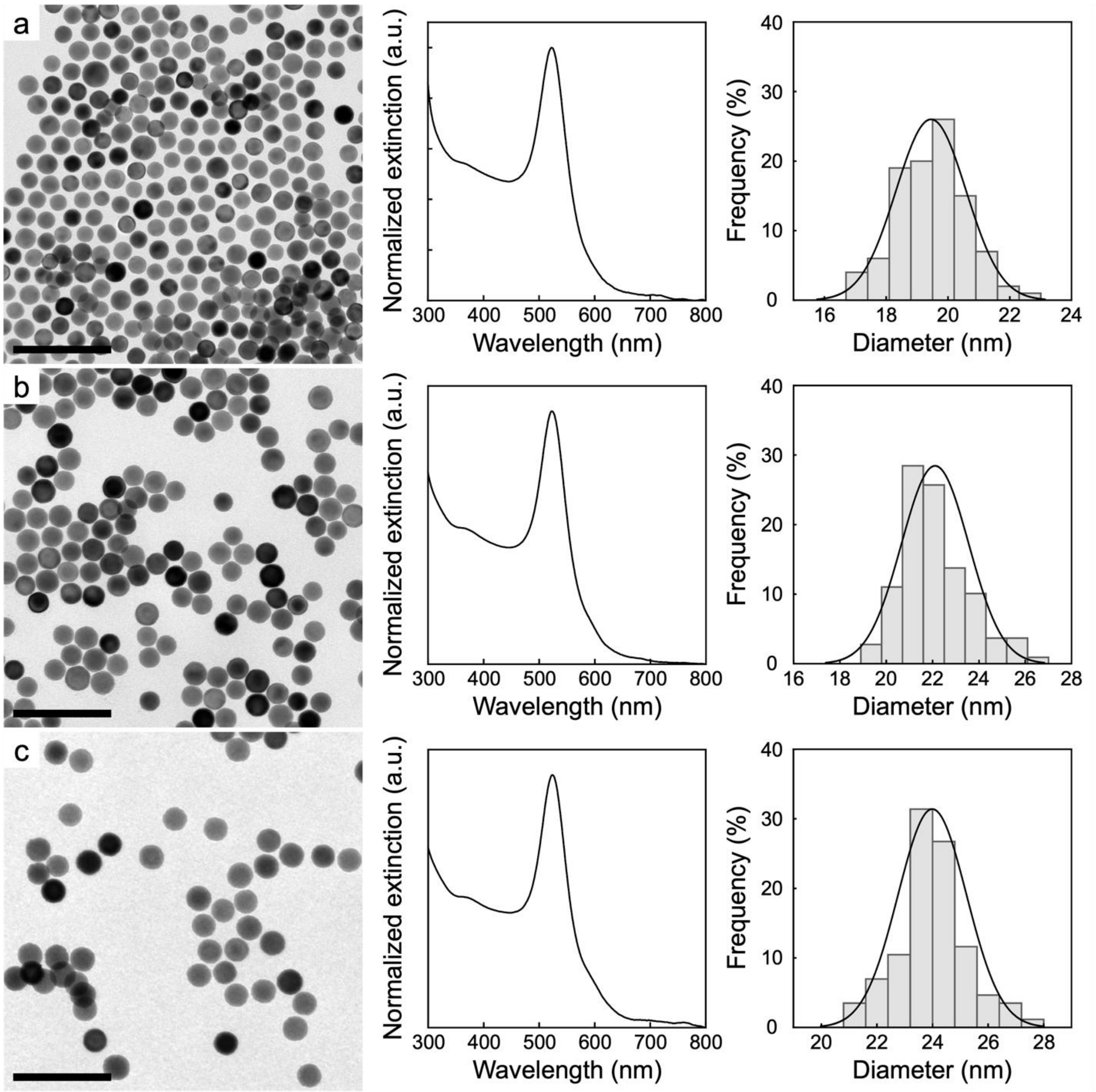

Supplementary Fig. 1. Universal seeds for the seeded growth of differently shaped NPs. Representative TEM images, normalized UV-Vis spectra, and diameters of (**a**) Universal seed I (λ_{max} = 522 nm) used for synthesis of gold octahedra and cubes; (**b**) Universal seed II (λ_{max} = 523 nm) used for synthesis of octahedra for the halide ion control experiment (**Supplementary Note 2.2**) and both small and large rhombic dodecahedra; and (**c**) Universal seed III (λ_{max} = 525 nm) used for synthesis of cuboctahedra. The diameter of each universal seed is (**a**) 19.5 ± 1.23 nm, (**b**) 22.1 ± 1.44 nm, and (**c**) 24.0 ± 1.23 nm, respectively. Scale bars: 100 nm.

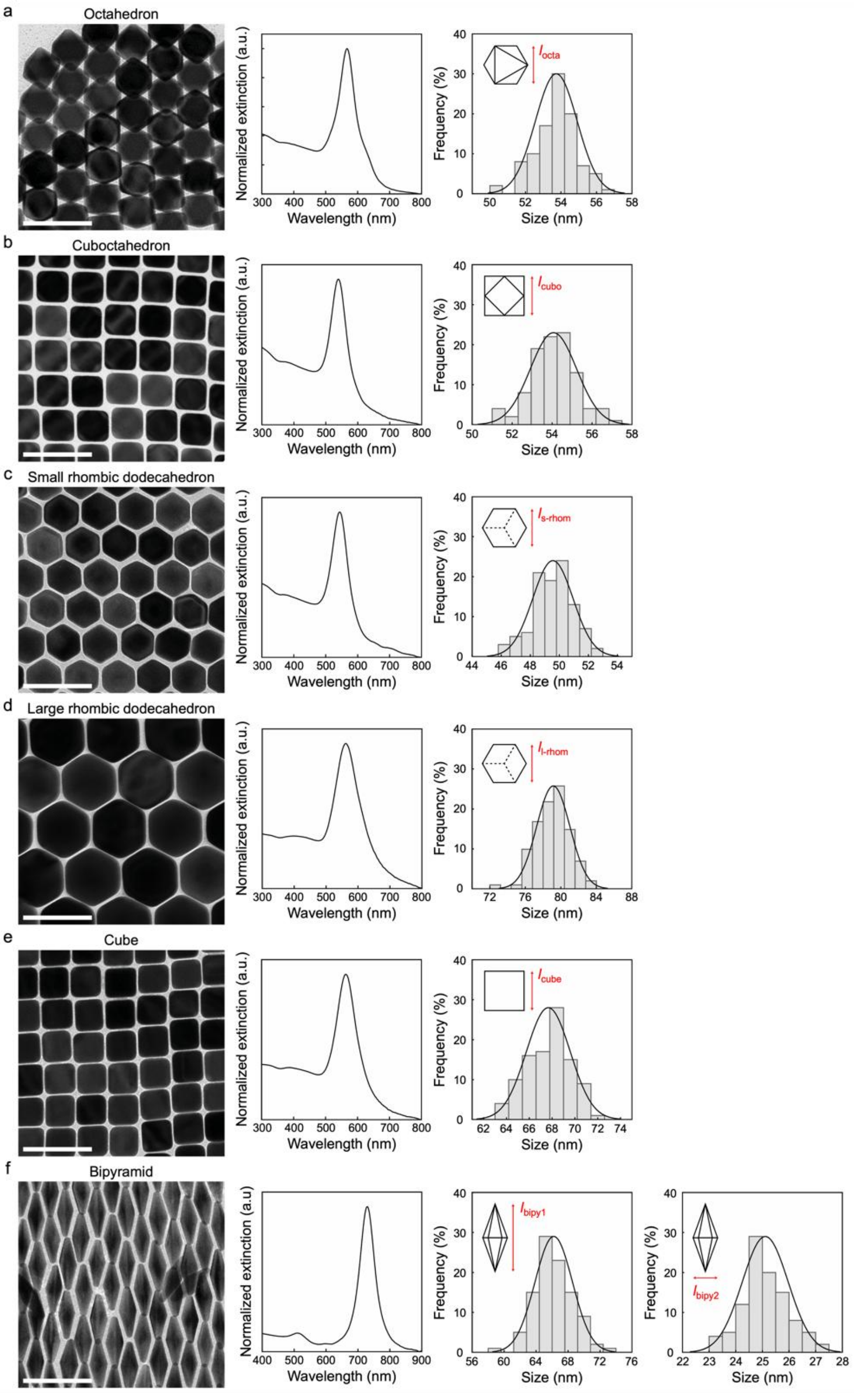

Supplementary Fig. 2. A library of differently shaped gold core NPs used for patchy NP synthesis. (**a**–**f**) Representative TEM images (left), normalized UV-Vis spectra (middle), and characteristic length (right) of gold NPs: octahedra (λ_{max} = 566 nm, edge length l_{octa} = 53.7 ± 1.17 nm) (**a**), cuboctahedra (λ_{max} = 539 nm, [100] projected width l_{cubo} = 54.1 ± 1.15 nm) (**b**), small rhombic dodecahedra (λ_{max} = 544 nm, [111] projected width $l_{s\text{-}rhom}$ = 49.6 ± 1.38 nm) (**c**), large rhombic dodecahedra (λ_{max} = 561 nm, $l_{l\text{-}rhom}$ = 79.1 ± 1.86 nm) (**d**), cubes (λ_{max} = 563 nm, edge length l_{cube} = 67.7 ± 1.91 nm) (**e**), and bipyramids (λ_{max} = 729 nm, height l_{bipy1} = 66.2 ± 2.34 nm, width l_{bipy2} = 25.1 ± 0.86 nm) (**f**). More than 70 NPs (**a**: 74 NPs; **b−f**: 100 NPs) are measured for each shape. Scale bars: 100 nm.

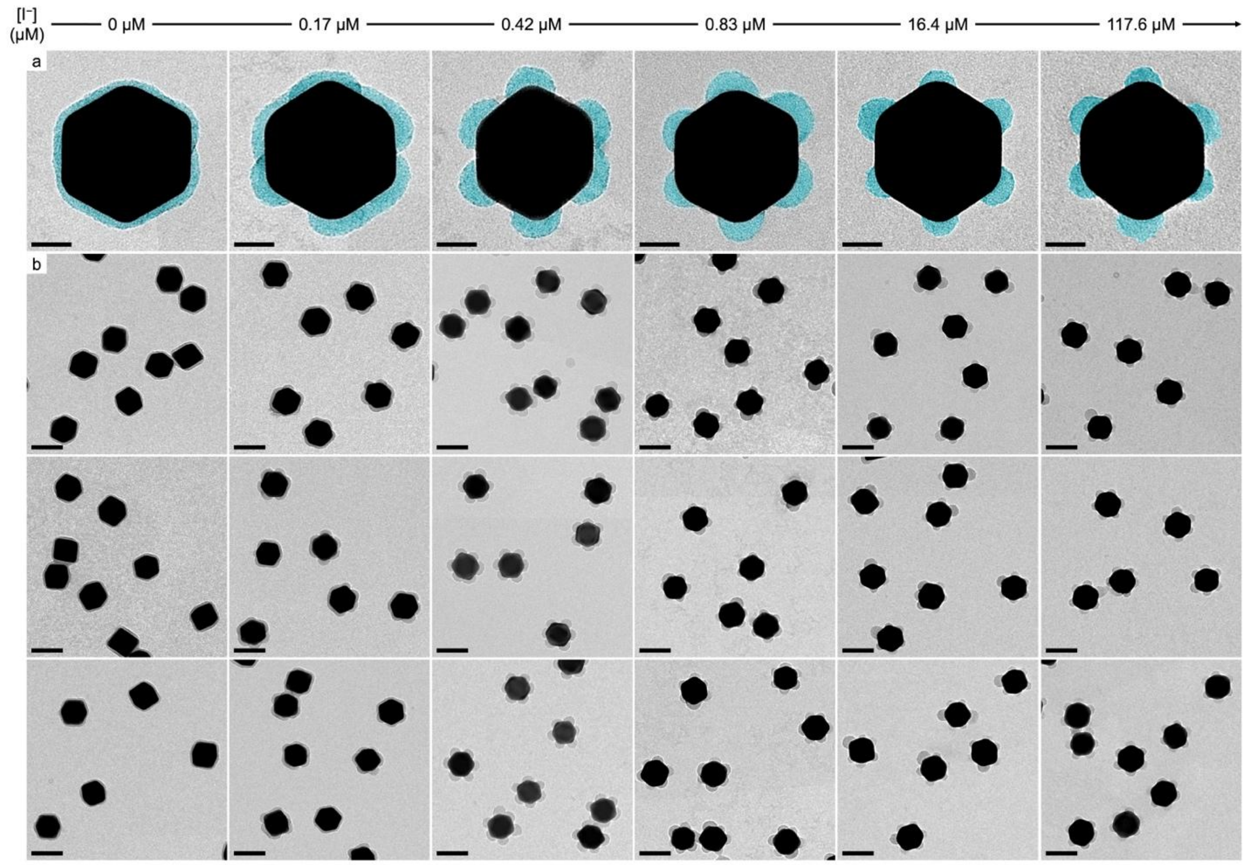

Supplementary Fig. 3. Iodide concentration effect on patch patterns on octahedra. (**a**) Representative TEM images of patchy octahedra synthesized with varying [I$^-$] in the masking step. (**b**) Low-magnification TEM images of corresponding patchy octahedra. As [I$^-$] increases from 0 to 0.42 μM, fully coated octahedra transition into vertex-patched structures. Further increase of [I$^-$] to 117.6 μM makes the patches smaller while keeping the vertex patch location. [2-NAT] is fixed at 11.3 μM. For detailed synthesis conditions, see **Supplementary Note 2.1** and **Supplementary Table 2**. Scale bars: (**a**) 20 nm and (**b**) 100 nm.

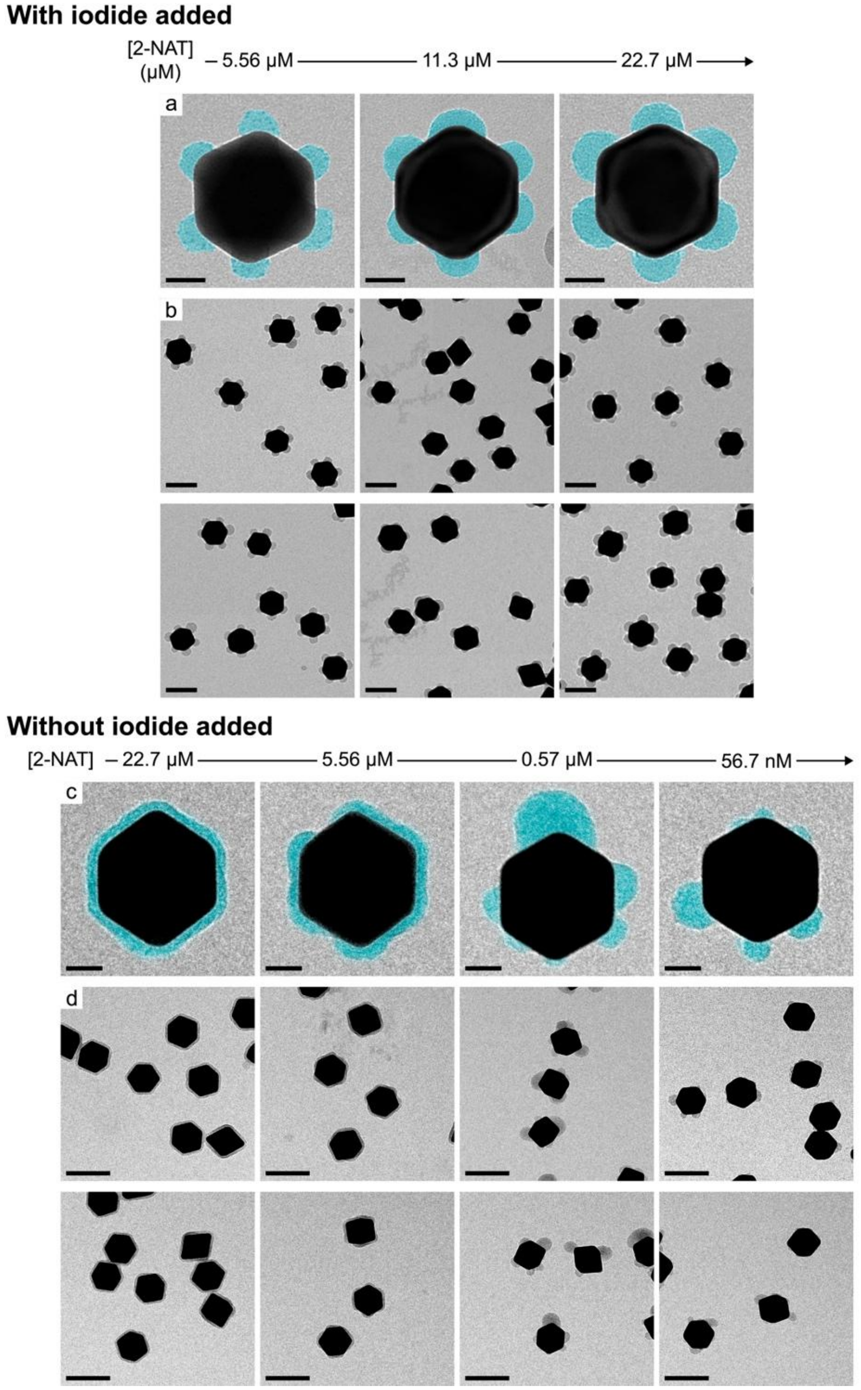

Supplementary Fig. 4. 2-NAT concentration effect on patch patterns on octahedra. (**a**) Representative TEM images of patchy octahedra synthesized with varying [2-NAT] with iodide masking. (**b**) Low-magnification TEM images of corresponding patchy octahedra. As [2-NAT] increases, patch thickness and coverage increase as well. [I^-] is fixed at 0.83 μM. (**c**) Representative TEM images of the control experiment on polymer-coated octahedra synthesized using varying [2-NAT] without iodide masking. (**d**) Low-magnification TEM images of the polymer-coated octahedra. In (**a)** and (**c**)**,** polymer patches are false-colored in cyan. For detailed synthesis conditions, see **Supplementary Note 2.1** and **Supplementary Table 2**. Scale bars: (**a,c**) 20 nm and (**b,d**) 100 nm.

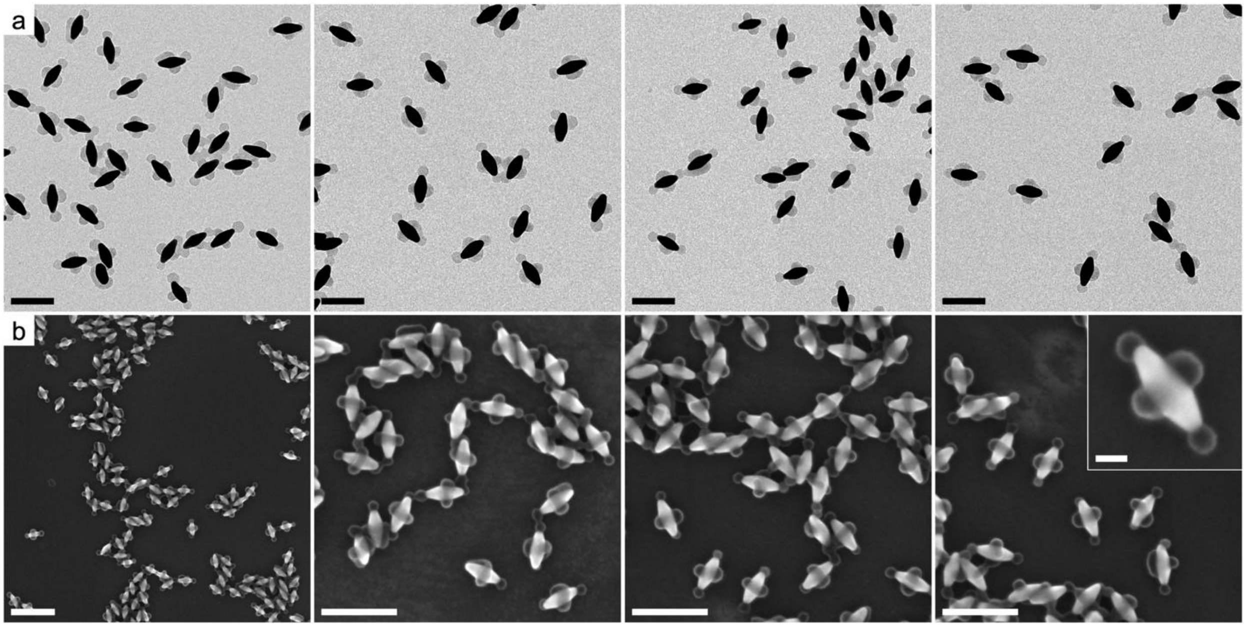

Supplementary Fig. 5. Synthesis of patchy bipyramids. (**a**,**b**) Representative TEM (**a**) and SEM (**b**) images of patchy bipyramids shown in **Fig. 3f**. Patches are formed on both tips and the equator of the bipyramids. For detailed synthesis conditions, see **Supplementary Note 2.3** and **Supplementary Table 4**. Scale bars: 100 nm (inset: 20 nm).

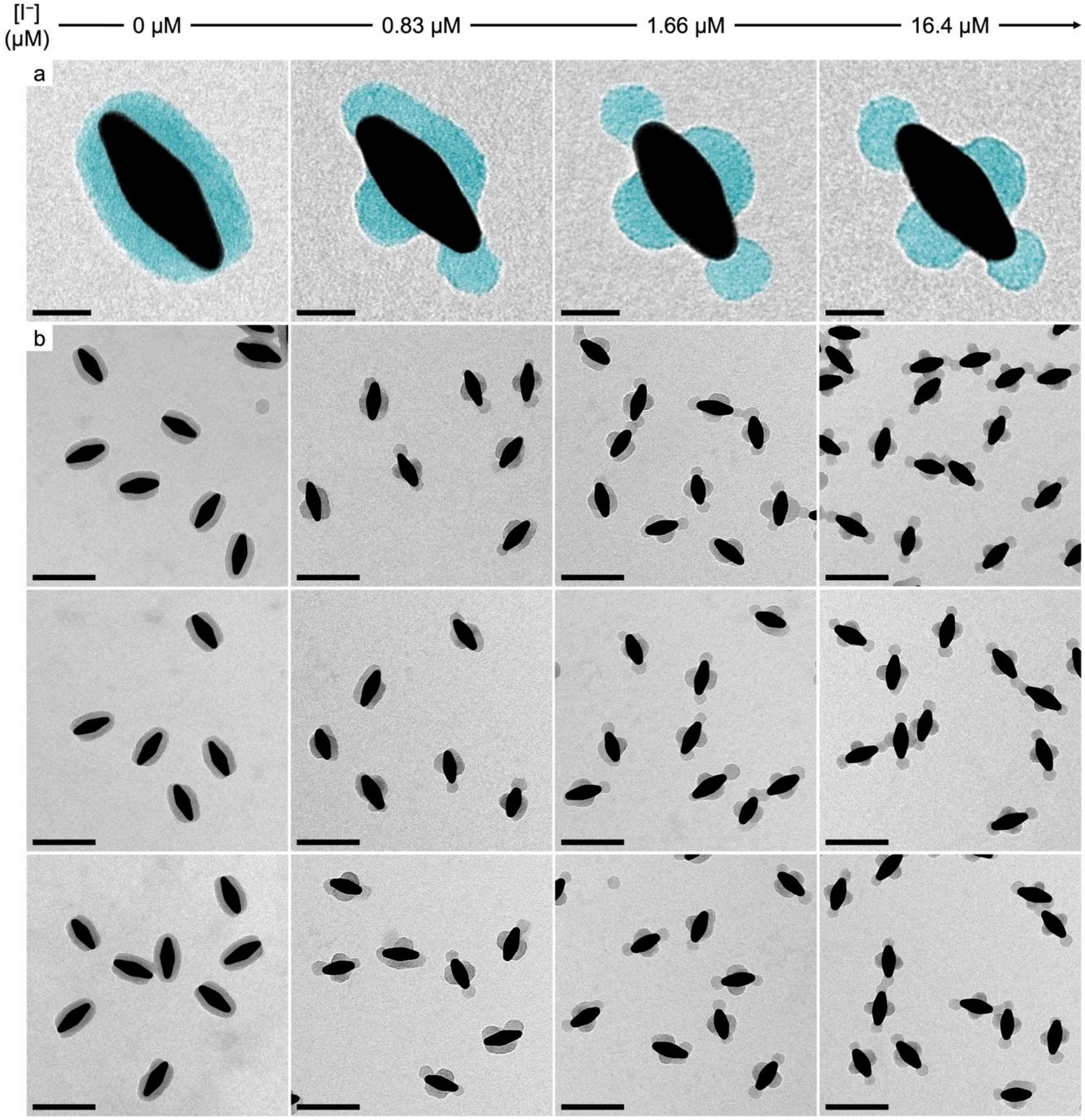

Supplementary Fig. 6. Iodide concentration effect on patch patterns on bipyramids. (**a**) Representative TEM images of patchy bipyramids synthesized with varying [I⁻] in the masking step. (**b**) Low-magnification TEM images of corresponding patchy bipyramids. As [I⁻] increases from 0 to 1.66 μM, fully coated bipyramids transition into patchy structures. Further increase of [I⁻] to 16.4 μM makes the patches smaller. [2-NAT] is fixed at 11.3 nM. For detailed synthesis conditions, see **Supplementary Note 2.3** and **Supplementary Table 4**. Scale bars: (**a**) 20 nm and (**b**) 100 nm.

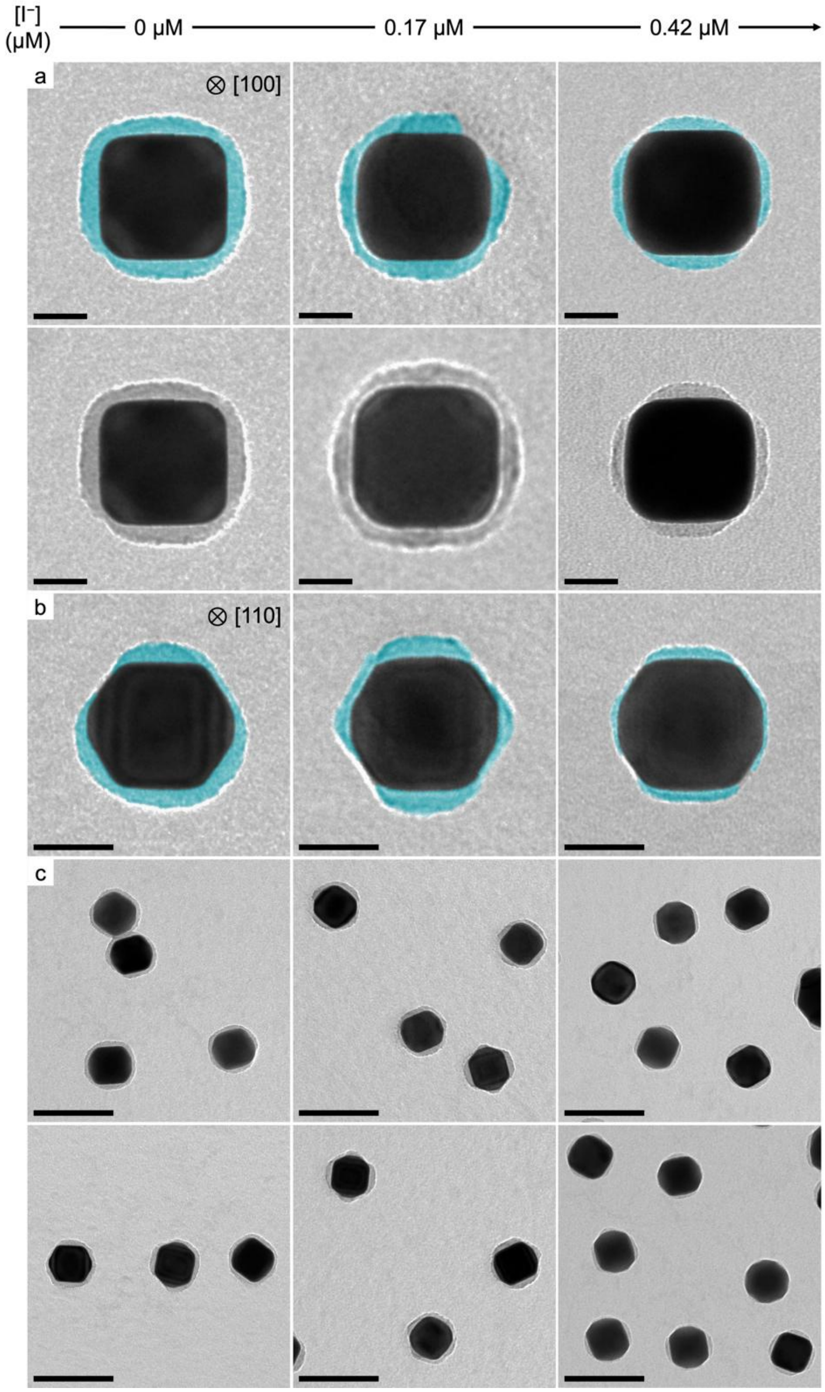

Supplementary Fig. 7. Iodide concentration effect on patch patterns on cuboctahedra at high 2-NAT concentration. (**a**,**b**) Representative TEM images of patchy cuboctahedra synthesized with varying [I^-] in the masking step, viewed along different orientations: [100] (**a**) and [110] (**b**). (**c**) Low-magnification TEM images of corresponding patchy cuboctahedra. As [I^-] increases, fully coated cuboctahedra transition into face-patched cuboctahedra. [2-NAT] is fixed at 22.7 μM. For detailed synthesis conditions, see **Supplementary Note 2.4** and **Supplementary Table 5**. Scale bars: (**a**,**b**) 20 nm and (**c**) 100 nm.

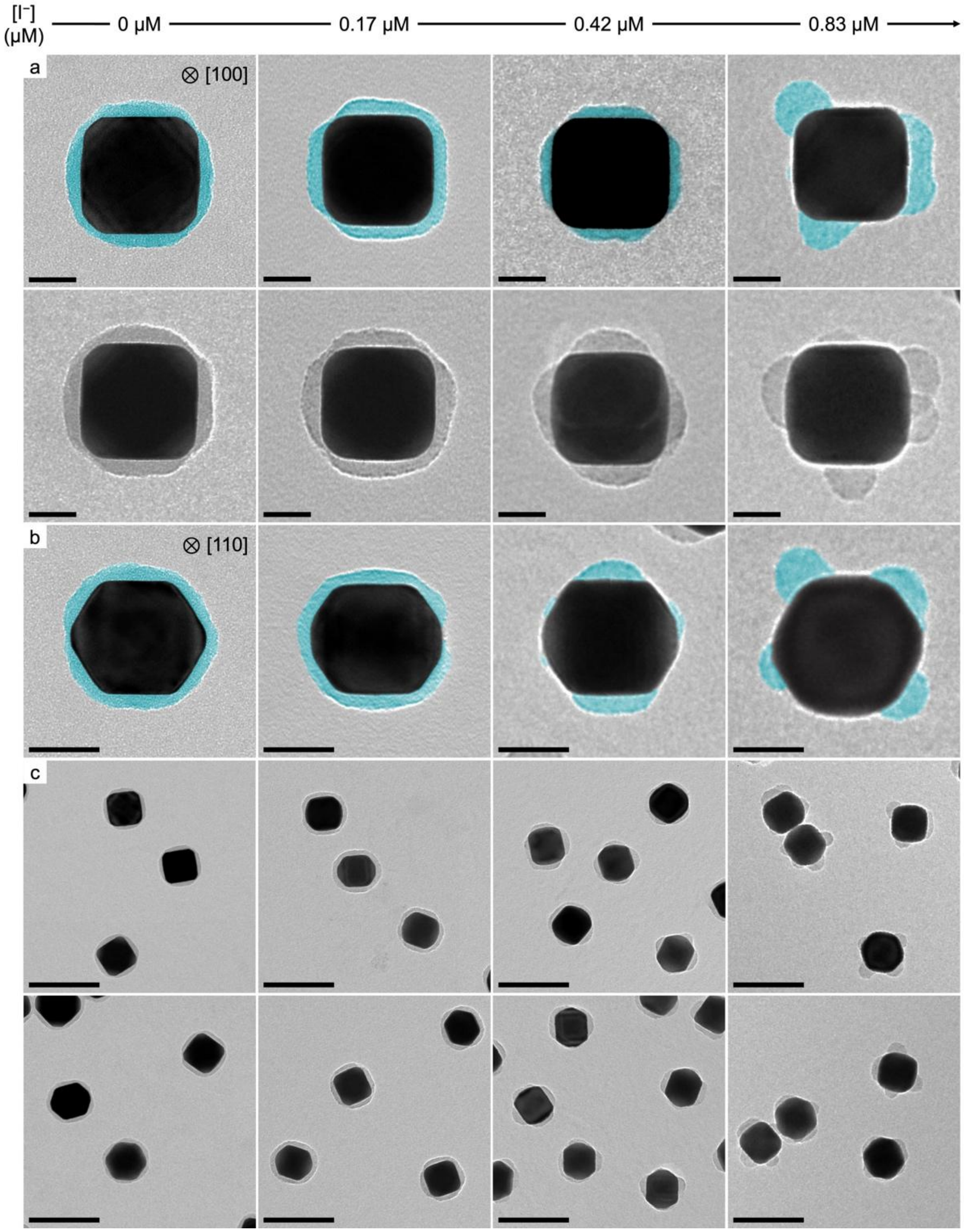

Supplementary Fig. 8. Iodide concentration effect on patch patterns on cuboctahedra at intermediate 2-NAT concentration. (**a**,**b**) Representative TEM images of patchy cuboctahedra synthesized with varying [I^-] in the masking step, viewed along different orientations: [100] (**a**) and [110] (**b**). (**c**) Low-magnification TEM images of corresponding patchy cuboctahedra. As [I^-] increases, fully coated cuboctahedra transition into face-patched cuboctahedra at 0.42 μM of [I^-]. However, a further increase of [I^-] to 0.83 μM leads to randomly positioned patches with both face and vertex patches on a single NP. [2-NAT] is fixed at 11.3 μM. For detailed synthesis conditions, see **Supplementary Note 2.4** and **Supplementary Table 5**. Scale bars: (**a**,**b**) 20 nm and (**c**) 100 nm.

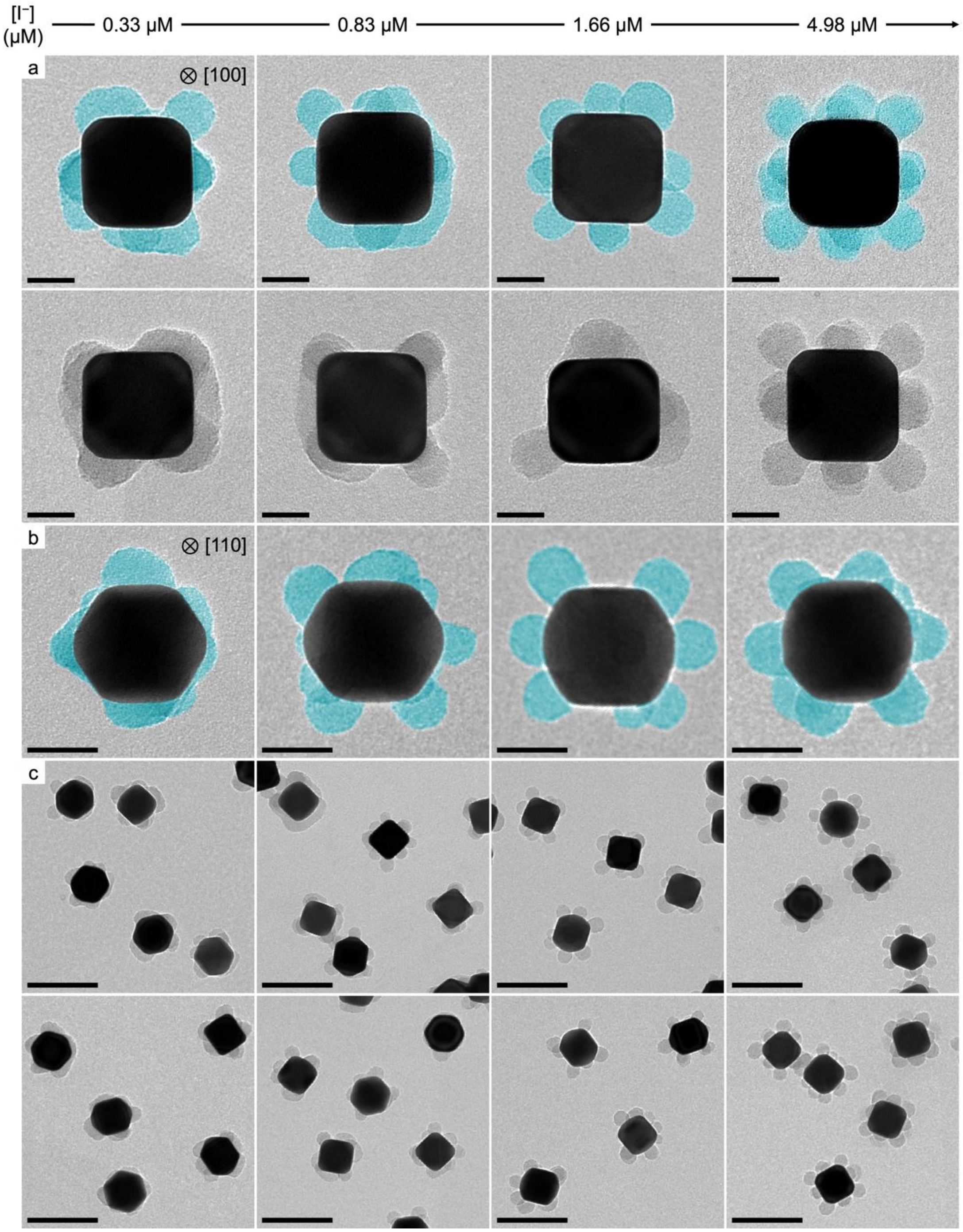

Supplementary Fig. 9. Iodide concentration effect on patch patterns on cuboctahedra at low 2-NAT concentration. (**a**,**b**) Representative TEM images of patchy cuboctahedra synthesized with varying [I^-] in the masking step, viewed along different orientations: [100] (**a**) and [110] (**b**). (**c**) Low-magnification TEM images of corresponding patchy cuboctahedra. At low [I^-], patches are randomly positioned on NP surface. At 4.98 μM of [I^-], monodisperse vertex-patched cuboctahedra are synthesized. [2-NAT] is fixed at 0.85 μM. For detailed synthesis conditions, see **Supplementary Note 2.4** and **Supplementary Table 5**. Scale bars: (**a**,**b**) 20 nm and (**c**) 100 nm.

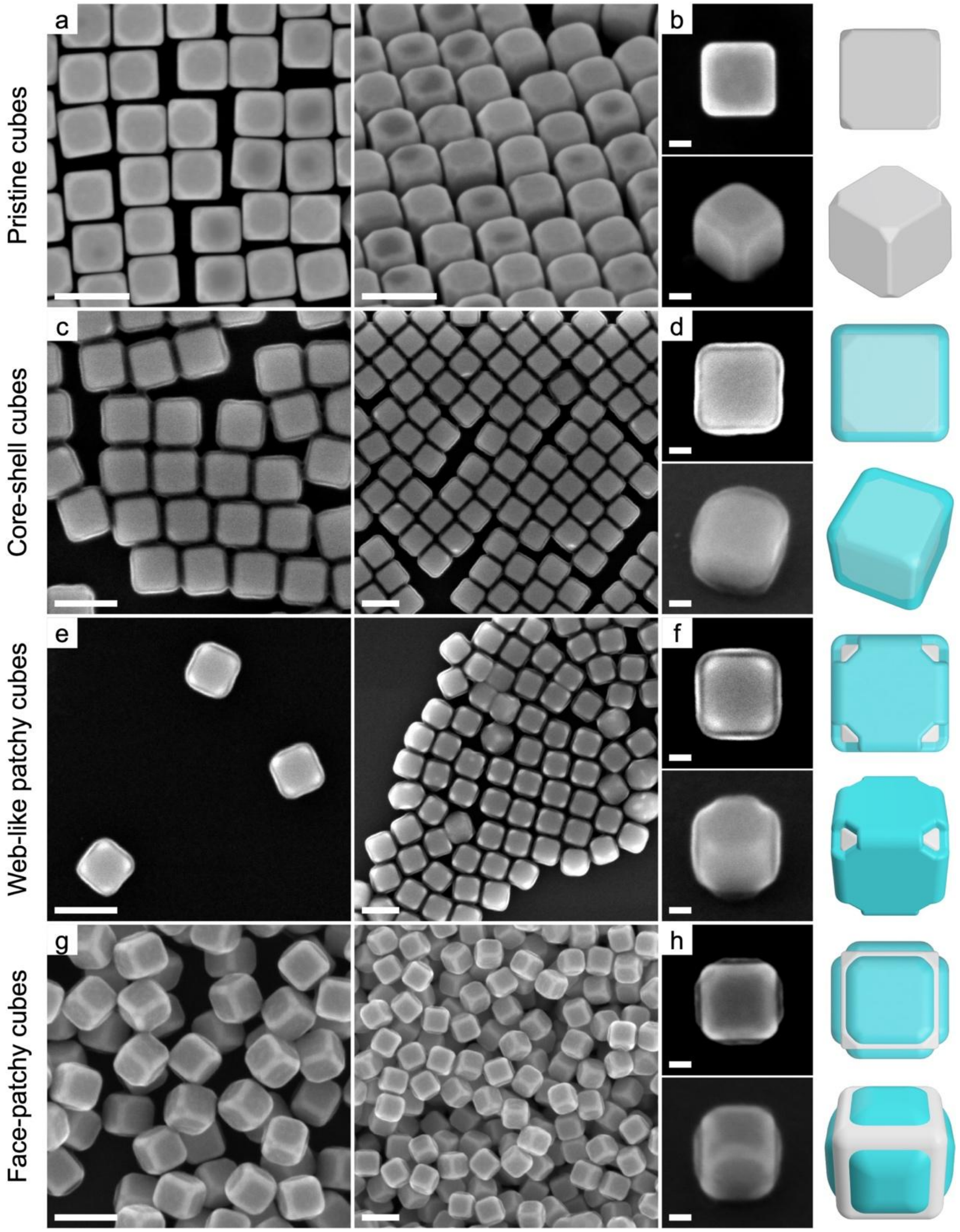

Supplementary Fig. 10. Synthesis of patchy cubes. (**a**,**b**) Representative SEM images of pristine cubes at low (**a**) and high (**b**) magnifications from different viewing angles, along with corresponding 3D models. (**c**−**h**) Representative SEM images of cubes after polymer grafting showing various patch patterns, along with corresponding 3D models: fully coated (**c**,**d**), web-like patchy (**e**,**f**), face-patched (**g**,**h**) cubes. As shown in (**a,b**), the vertices of cubes are truncated rather than rounded, which is consistent with previous literature.[2,30] For detailed synthesis conditions, see **Supplementary Note 2.5** and **Supplementary Table 6**. Scale bars: (**a**,**c**,**e**,**g**) 100 nm and (**b**,**d**,**f**,**h**) 20 nm.

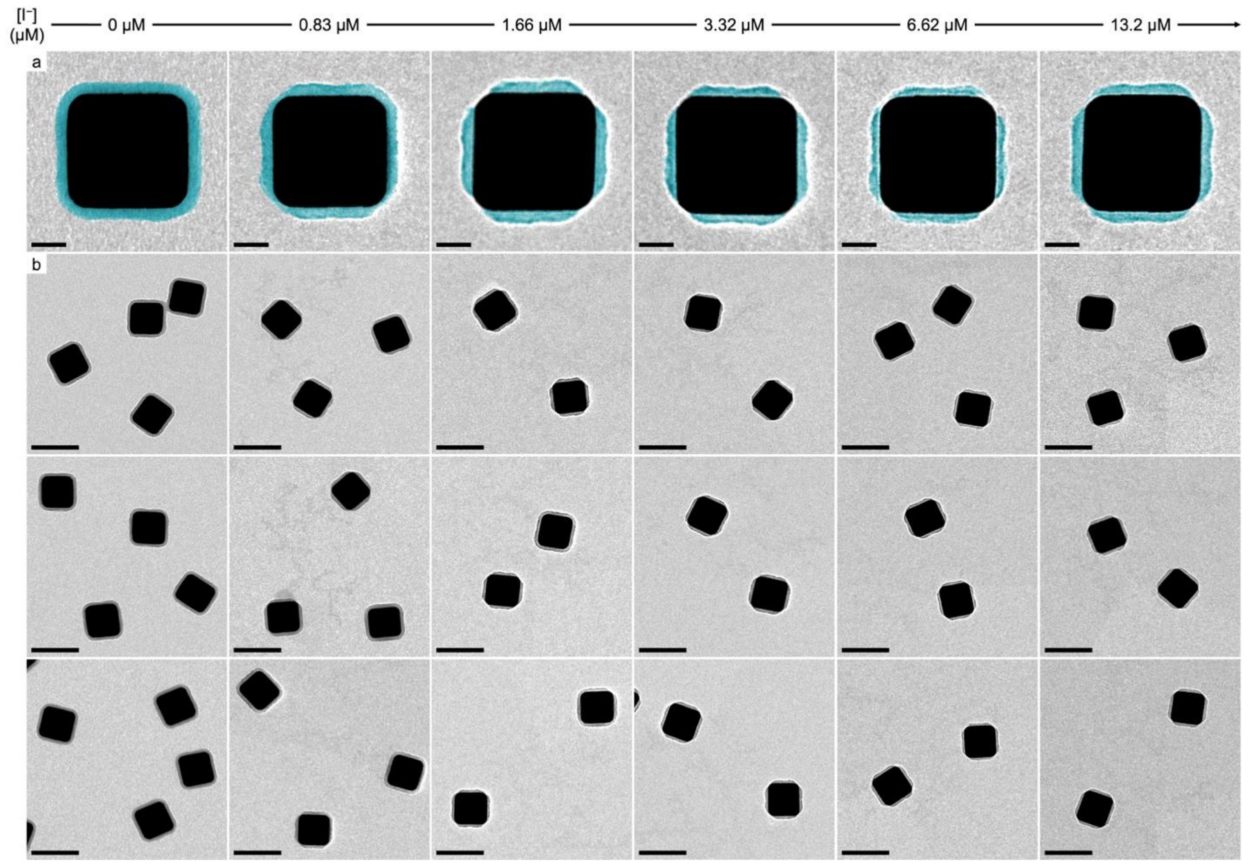

Supplementary Fig. 11. Iodide concentration effect on patch patterns on cubes. (**a**) Representative TEM images of patchy cubes synthesized with varying [I⁻] in the masking step. (**b**) Low-magnification TEM images of corresponding patchy cubes. As [I⁻] increases from 0 to 13.2 μM, fully coated cubes transition into face-patched structures. [2-NAT] is fixed at 22.7 μM. For detailed synthesis conditions, see **Supplementary Note 2.5** and **Supplementary Table 6**. Scale bars: (**a**) 20 nm and (**b**) 100 nm.

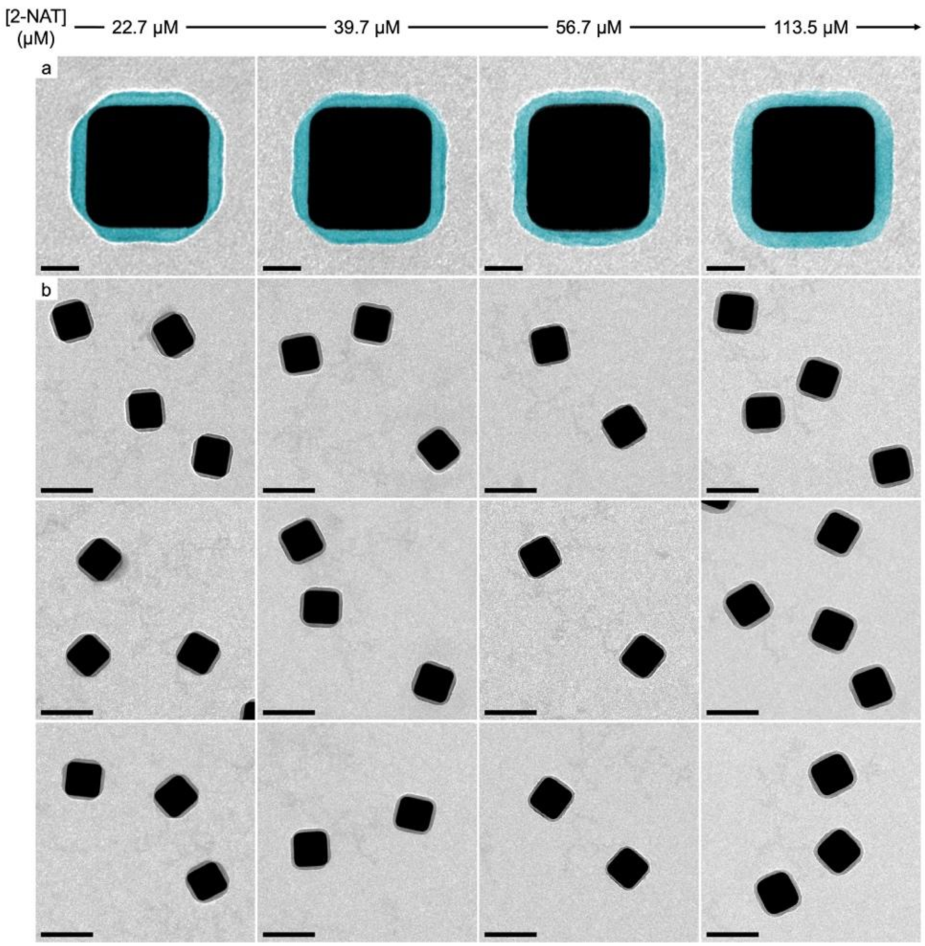

Supplementary Fig. 12. Ligand concentration effect on patch patterns on cubes. (**a**) Representative TEM images of patchy cubes synthesized with increasing [2-NAT]. (**b**) Low-magnification TEM images of corresponding patchy cubes. As [2-NAT] increases, patch thickness and coverage on cubes are increased. [I^-] is fixed at 0.83 μM. For detailed synthesis conditions, see **Supplementary Note 2.5** and **Supplementary Table 6**. Scale bars: (**a**) 20 nm and (**b**) 100 nm.

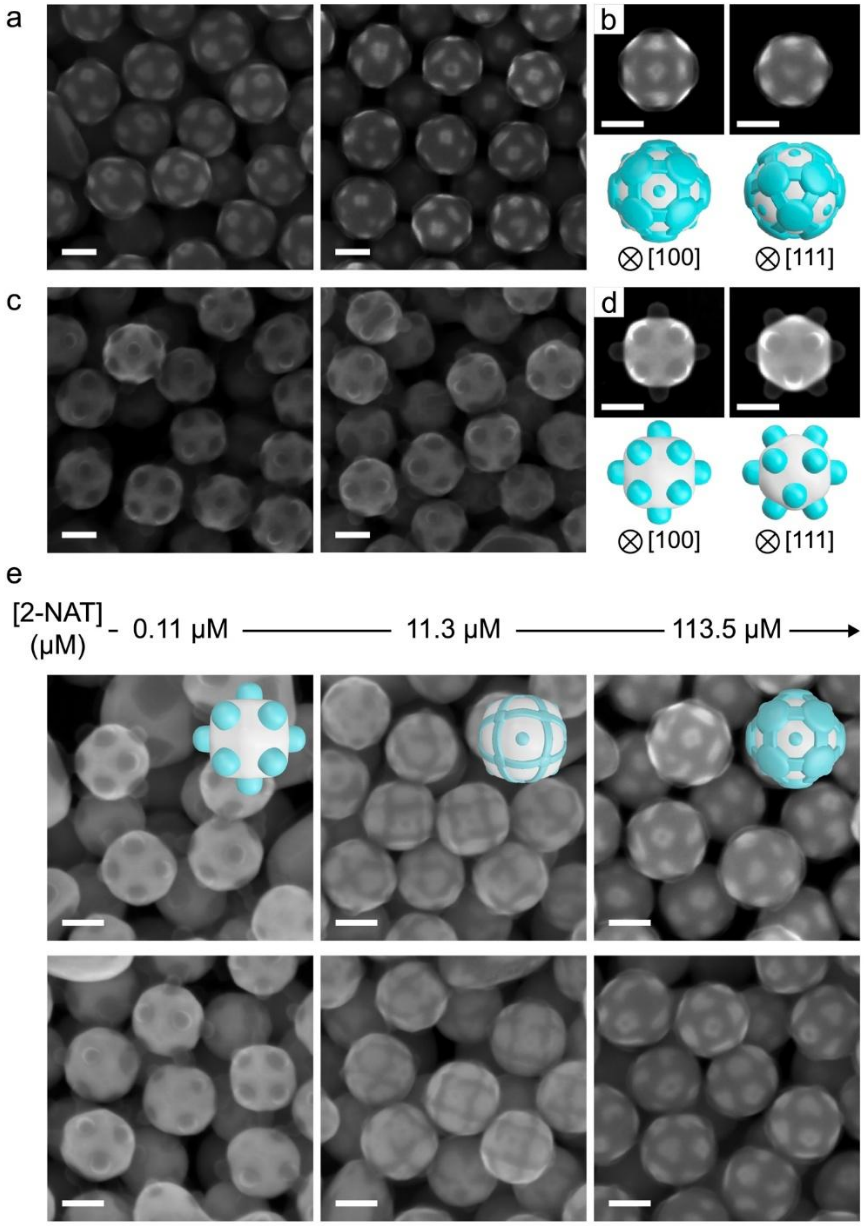

Supplementary Fig. 13. SEM images of patchy large rhombic dodecahedra. (**a**−**d**) SEM images and corresponding 3D models of large rhombic dodecahedra with web-like (**a**,**b**) and discrete patches on {110} faces (**c**,**d**). [I⁻] and [2-NAT] are 8.26 μM and 113.47 μM; and 0.50 μM and 1.13 μM for web-like and discrete patches, respectively. Note that the small patches in (**a**,**b**) are formed on six {100} vertices. (**e**) Representative SEM images of patchy large rhombic dodecahedra synthesized using varying [2-NAT] at a [I⁻] fixed of 8.33 μM (inset: corresponding schematic of patchy NPs). As [2-NAT] increases, the twelve face patches expand their size, merge together, and web-like patch pattern is formed. For synthesis conditions, see **Supplementary Note 2.6** and **Supplementary Table 7.** Scale bars: 50 nm.

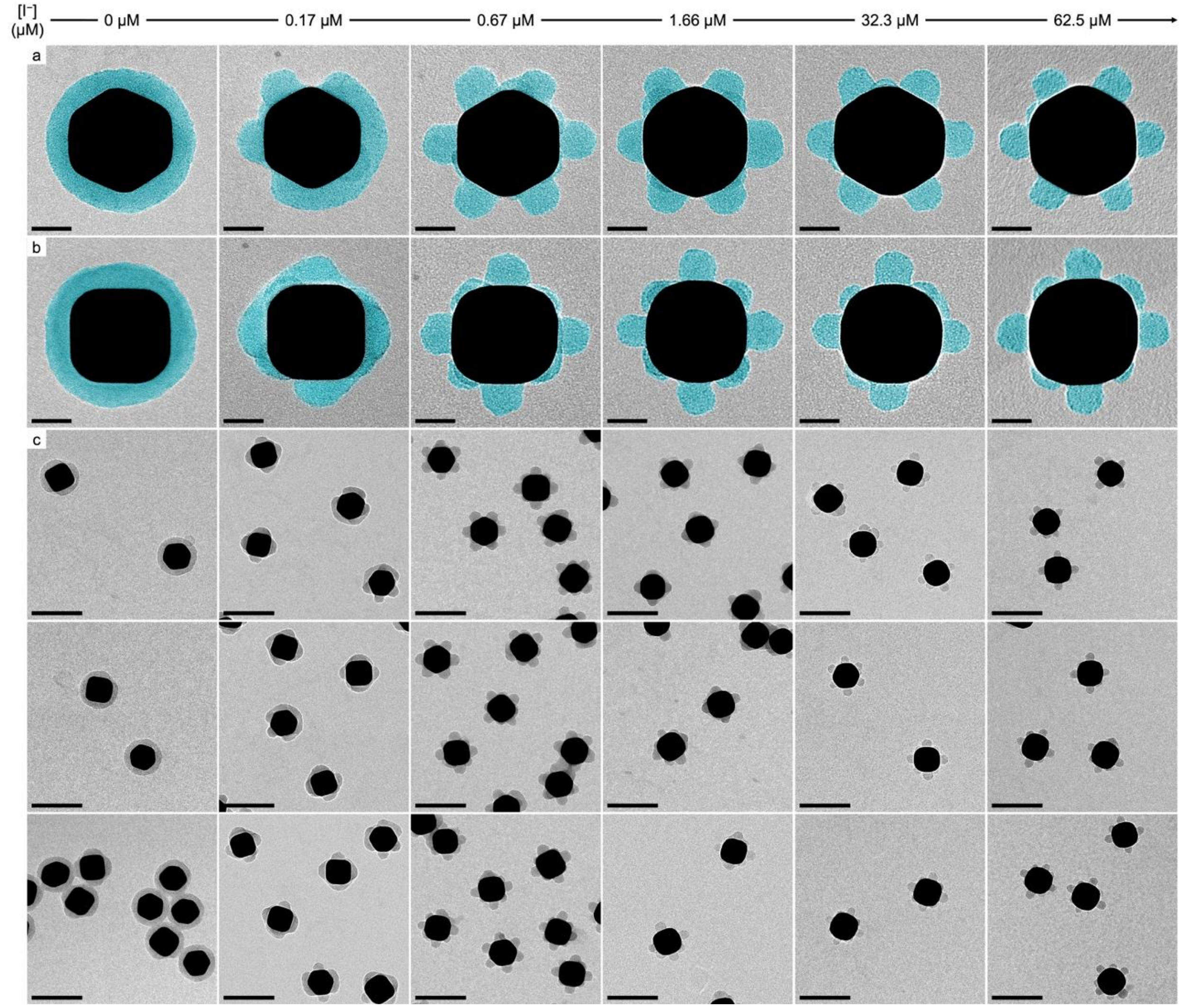

Supplementary Fig. 14. Iodide concentration effect on patch patterns on small rhombic dodecahedra. (**a**,**b**) Representative TEM images of patchy rhombic dodecahedra synthesized with increasing [I⁻] for masking, viewed along different orientations: [100] (**a**) and [110] (**b**). (**c**) Low-magnification TEM images of corresponding patchy rhombic dodecahedra. As [I⁻] increases from 0 to 0.67 μM, fully coated rhombic dodecahedra transition into face-patched structures. Further increase of [I⁻] to 62.5 μM makes the patches smaller while keeping the face patch location on NPs. [2-NAT] is fixed at 56.7 nM. For detailed synthesis conditions, see **Supplementary Note 2.7** and **Supplementary Table 8**. Scale bars: (**a,b**) 20 nm and (**c**) 100 nm.

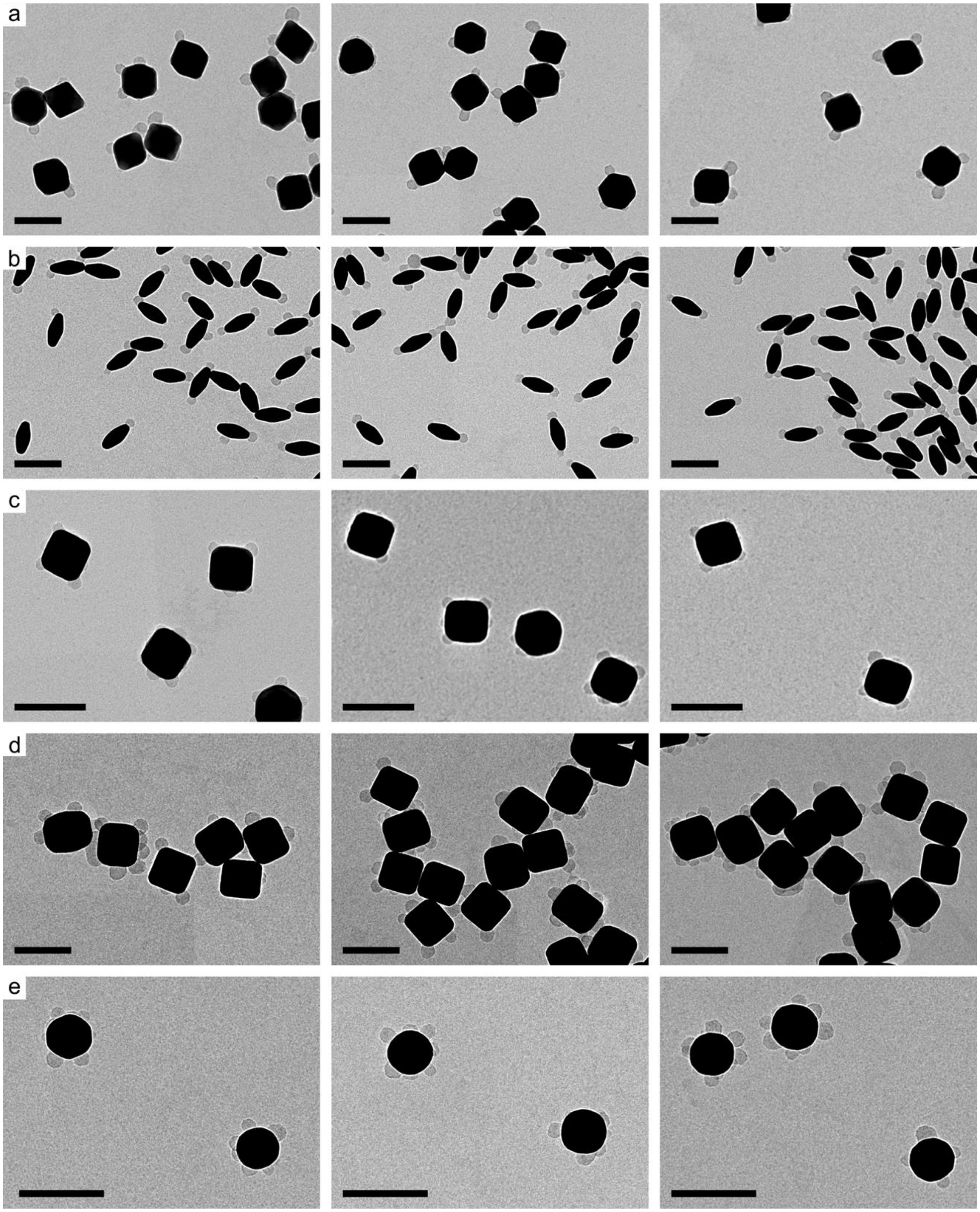

Supplementary Fig. 15. Symmetry-broken patchy NPs. (**a**−**e**) Representative TEM images of symmetry-broken patchy octahedra (**a**), bipyramids (**b**), cuboctahedra (vertex-patched) (**c**), cubes (**d**), and rhombic dodecahedra (**e**). For detailed synthesis conditions, see **Supplementary Notes 2.1**,**2.3**,**2.4**,**2.5**,**2.7** and **Supplementary Tables 2**,**4**,**5**,**6**,**8**. Scale bars: 100 nm.

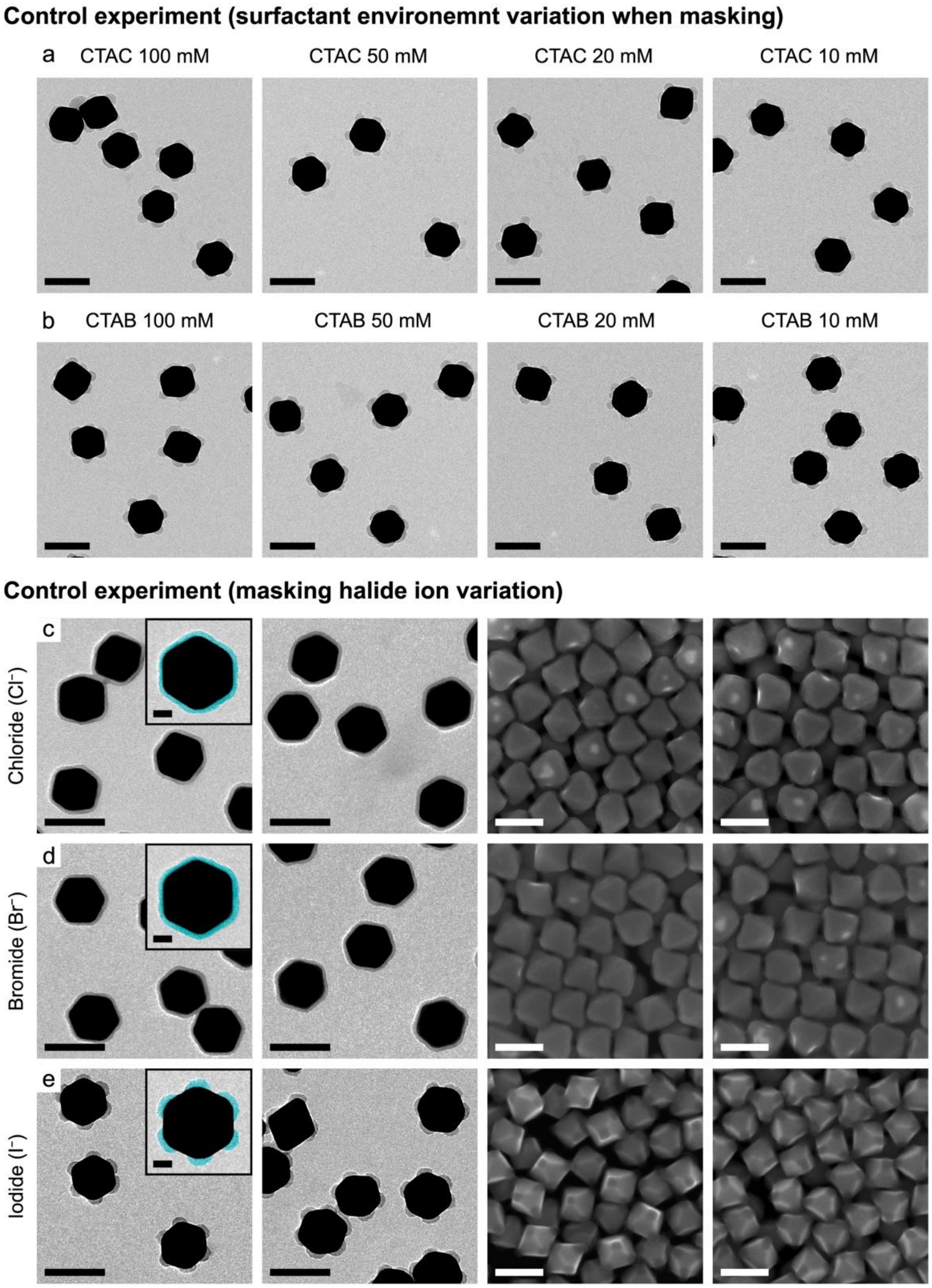

Supplementary Fig. 16. Control studies on surfactant conditions and halide ion specificity in masking. (**a**,**b**) Representative TEM images of patchy octahedra with iodide masking performed in different surfactant solutions: CTAC (**a**) and CTAB (**b**) at varying concentrations. The same vertex-patched octahedra are synthesized regardless of the surfactant type or concentration during iodide masking. [I^-] and [2-NAT] are fixed at 6.62 μM and 17.0 μM, respectively. (**c**–**e**) Representative TEM and SEM images of polymer-coated octahedra synthesized after incubation with different halide ions: chloride (**c**), bromide (**d**), and iodide (**e**). Vertex-patched octahedra are synthesized only when incubated with iodide. The concentrations of the halide ions and 2-NAT are fixed at 0.25 μM and 17.0 μM, respectively. Scale bars: 100 nm (inset: 20 nm).

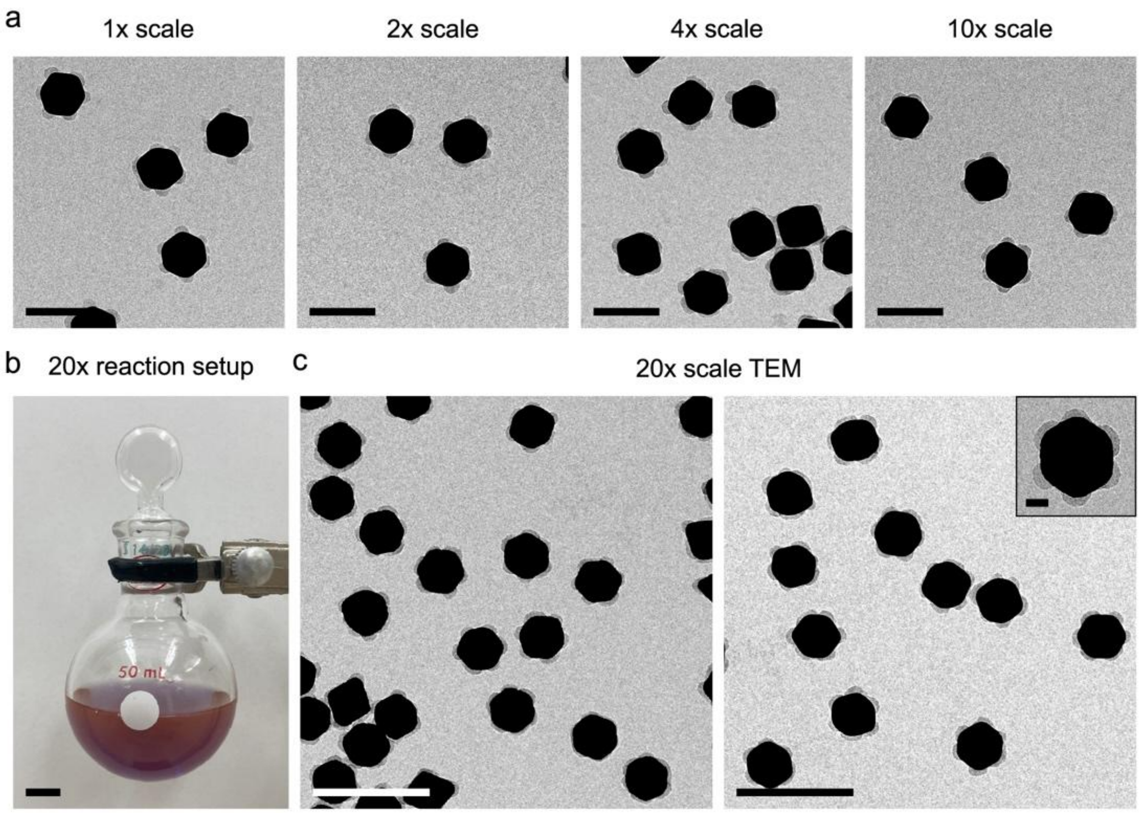

Supplementary Fig. 17. Scale-up synthesis of patchy gold octahedra. (**a**) TEM images of patchy octahedra synthesized at different reaction volumes, scaled up from the standard procedure (detailed process described in **Supplementary Note 2.9**). (**b**) Photograph of the reaction setup for a 20-fold scale-up reaction of patchy octahedra synthesis. (**c**) Representative TEM images of patchy octahedra synthesized at the 20-fold scale (inset: high-magnification TEM image). Vertex-patched octahedra are obtained consistently, showing that our atomic stenciling method is scalable. Scale bars: (**a**) 100 nm, (**b**) 1 cm, and (**c**) 200 nm (inset in **c**: 20 nm).

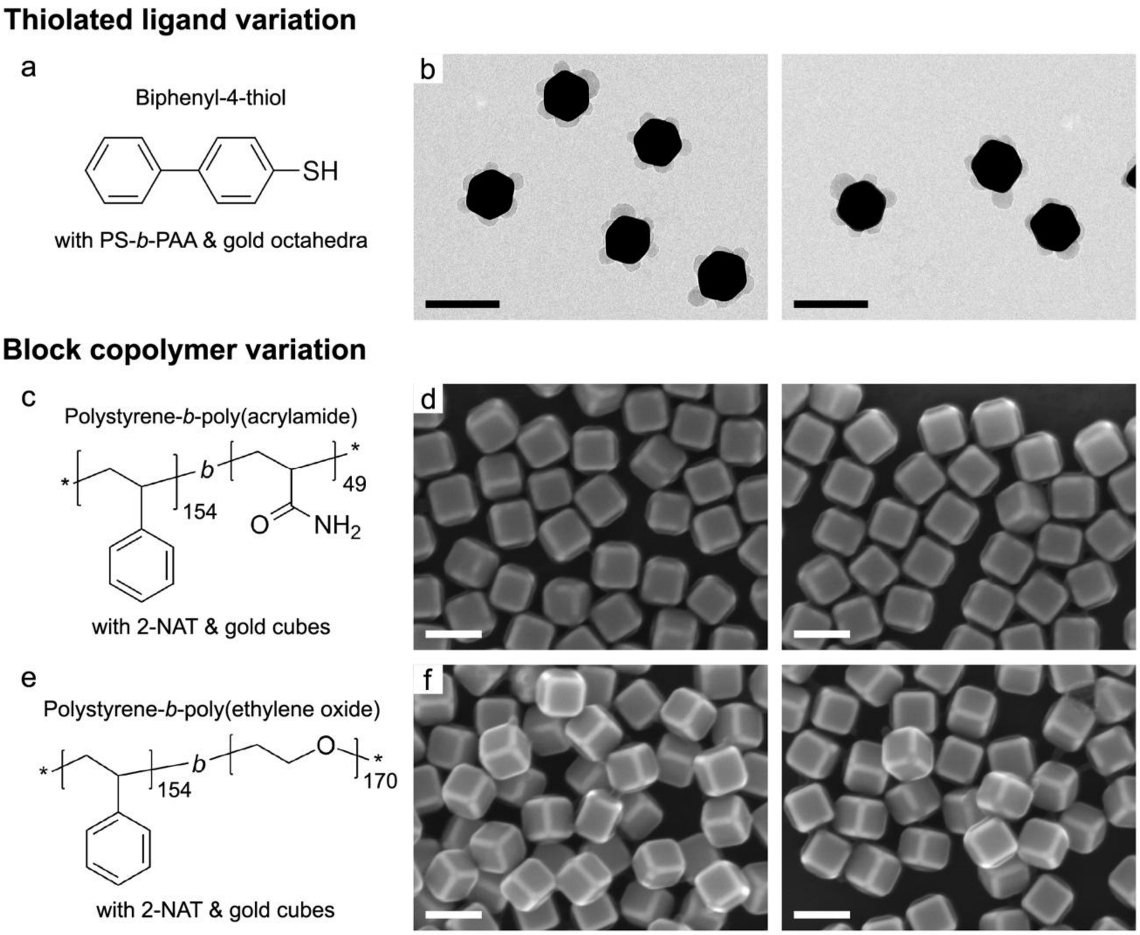

Supplementary Fig. 18. Synthesis of patchy NPs with different ligands and block copolymers. (**a**,**b**) Chemical structure of biphenyl-4-thiol (**a**) and TEM images of patchy octahedra synthesized using biphenyl-4-thiol together with PS-*b*-PAA (**b**). The NPs exhibit the same vertex patches as those synthesized at the same condition (but using 2-NAT as the thiol ligands instead), which is listed in **Supplementary Table 2**. (**c**,**d**) Chemical structure of PS-*b*-poly(acrylamide) (**c**) and SEM images of patchy cubes synthesized using PS-*b*-poly(acrylamide) together with 2-NAT (**d**). (**e**,**f**) Chemical structure of PS-*b*-poly(ethylene oxide) (**e**) and SEM images of patchy cubes synthesized using PS-*b*-poly(ethylene oxide) together with 2-NAT (**f**). These NPs using different block copolymers exhibit the same face patches as those synthesized using the same reaction conditions (but using PS-*b*-PAA instead), which are listed in **Supplementary Table 6**. Scale bars: 100 nm.

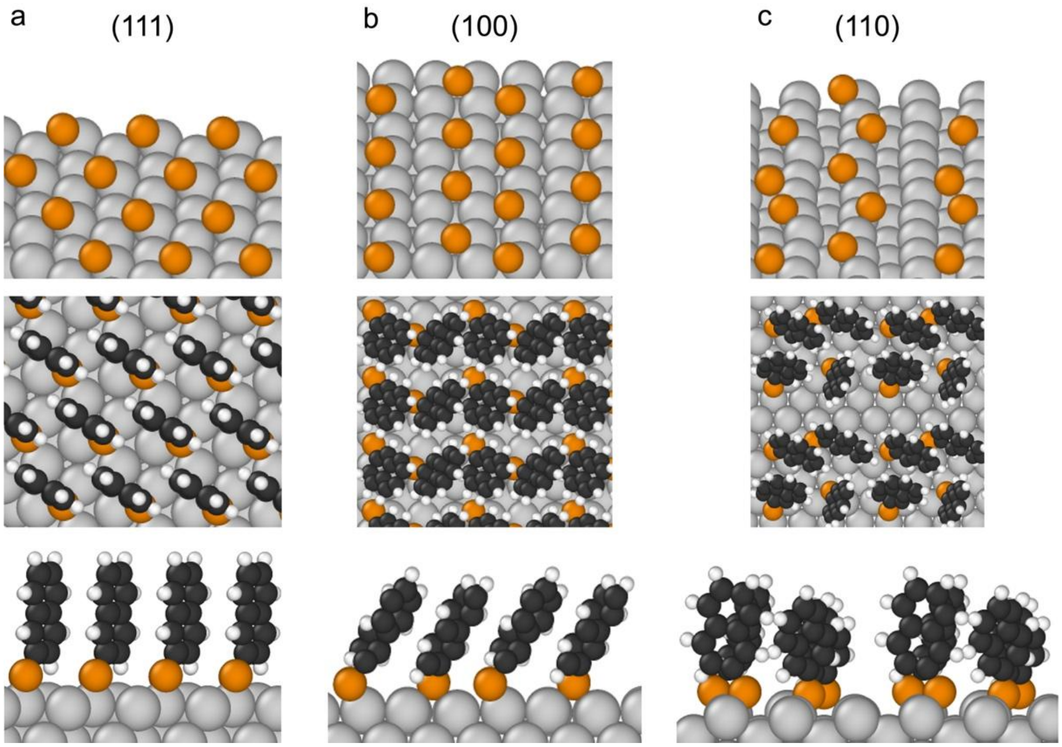

Supplementary Fig. 19. Optimized adsorption configuration at a 1/3 ML surface coverage of 2-NAT without iodide on gold surface, predicted by DFT. (**a**−**c**) The top row shows the locations of adsorbed sulfur in 2-NAT on Au (**a**) (111), (**b**) (100), and (**c**) (110) facets. For visualization, the orientation of the gold surface view is tilted, and the carbon and hydrogen atoms in 2-NAT are omitted to provide clearer views of the 2-NAT locations. The middle and bottom rows show 2-NAT molecules in their optimal configurations from top and side views, respectively. Silver: Au surface atom, black: C atom, white: H atom, orange: chemisorbed S atom. Note that the top row presents the extended data shown in **Fig. 2a** (top).

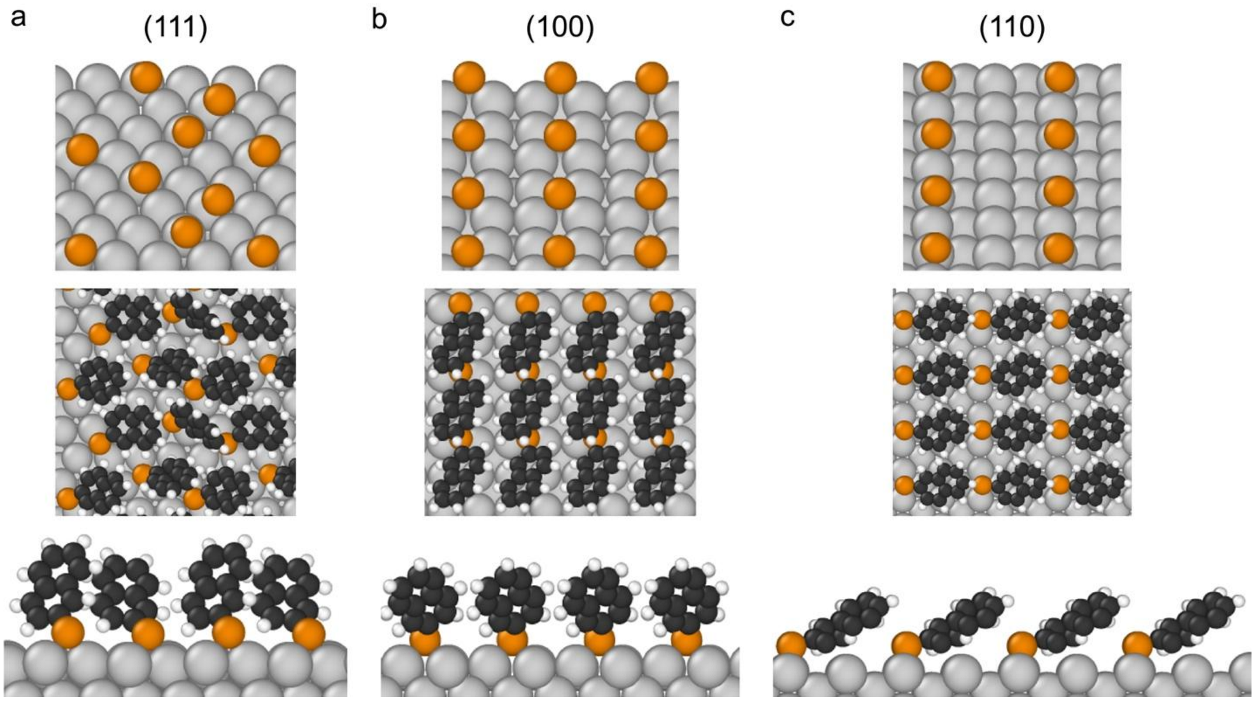

Supplementary Fig. 20. Optimized adsorption configuration at a 1/4 ML surface coverage of 2-NAT without iodide on gold surface, predicted by DFT. (**a**−**c**) The top row shows the locations of adsorbed sulfur in 2-NAT on Au (**a**) (111), (**b**) (100), and (**c**) (110) facets. For the visualization, the orientation of the gold surface view is tilted, and the carbon and hydrogen atoms in 2-NAT are omitted to provide clearer view of the 2-NAT locations. The middle and bottom rows show 2-NAT molecules in their optimal configurations from top and side views, respectively. Silver: Au surface atom, black: C atom, white: H atom, orange: chemisorbed S atom.

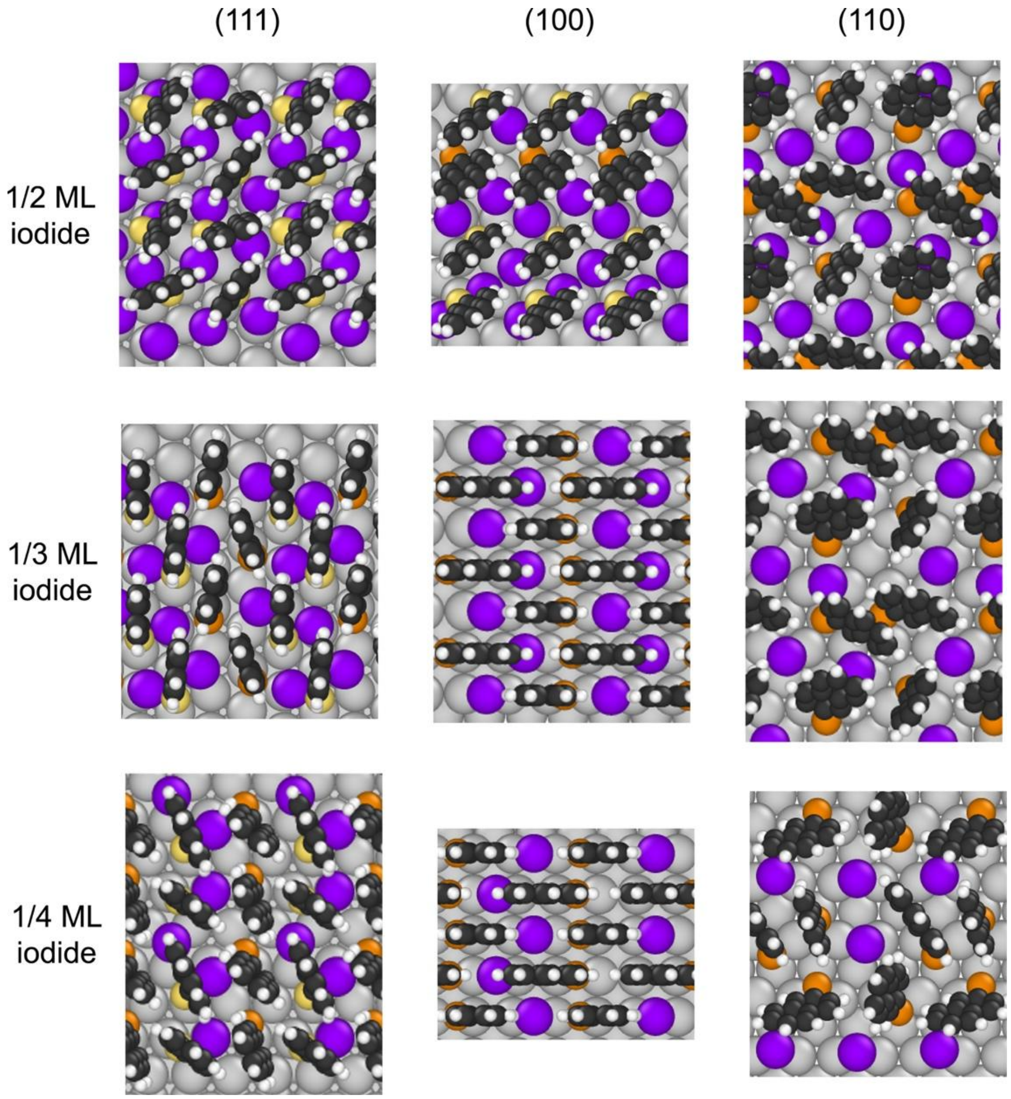

Supplementary Fig. 21. Optimized adsorption configuration at a 1/3 ML surface coverage of 2-NAT with iodide at various surface coverages on gold surface, predicted by DFT. DFT studies show the locations of co-adsorbed iodide and 2-NAT on Au (111) (left), (100) (middle), and (110) (right) facets at varying iodide surface coverage and a fixed 2-NAT surface coverage of 1/3 ML. Silver: Au surface atom, black: C atom, white: H atom, orange: chemisorbed S atom, purple: I atom. Note that the side views of 1/2 ML of iodide surface coverage condition are shown in **Fig. 2a** (bottom). The side views of 1/3 ML and 1/4 ML iodide surface coverage conditions are shown in **Extended Data Fig. 1**.

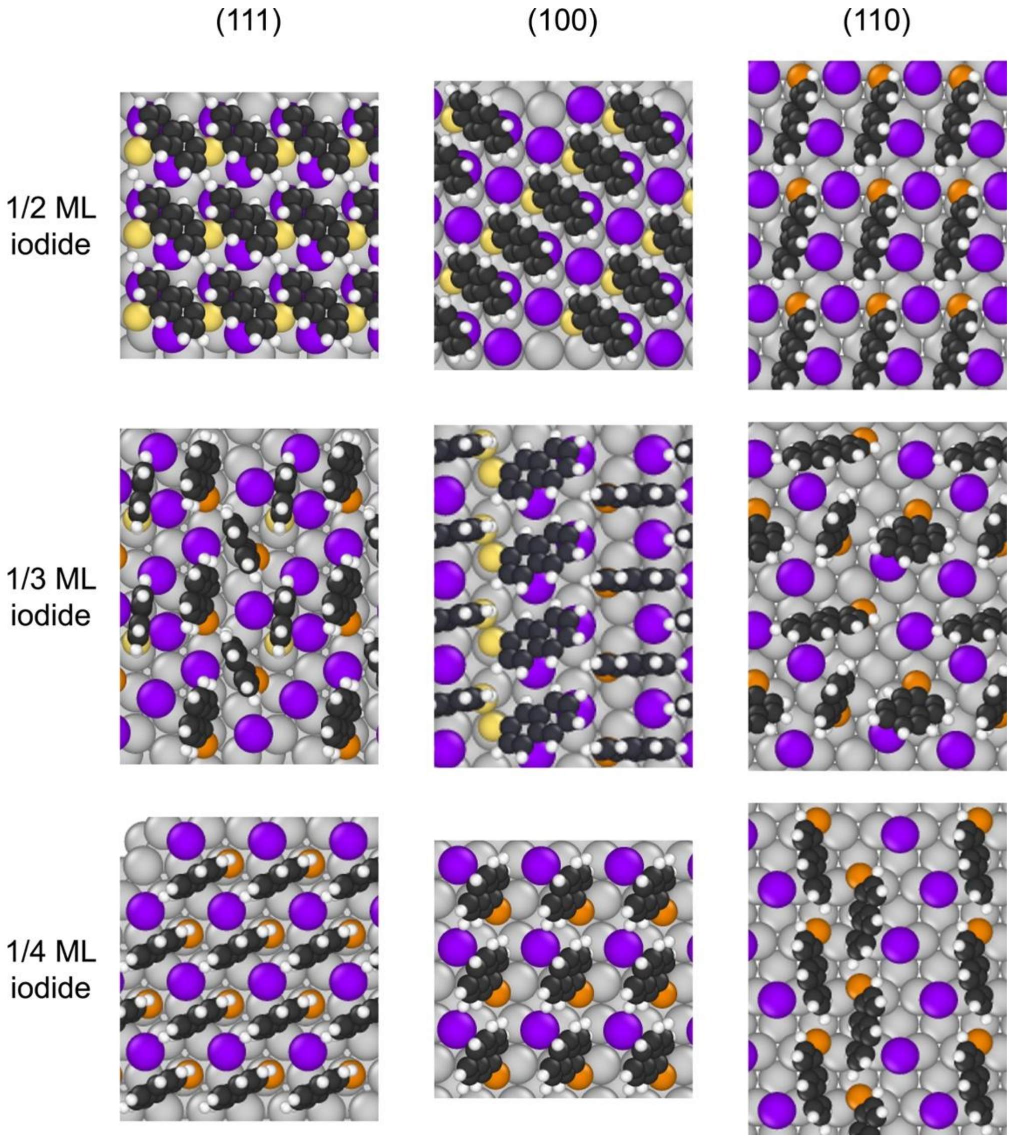

Supplementary Fig. 22. Optimized adsorption configuration at a 1/4 ML surface coverage of 2-NAT with iodide at various surface coverages on gold surface, predicted by DFT. DFT studies show the locations of co-adsorbed iodide and 2-NAT on Au (111) (left), (100) (middle), and (110) (right) facets at varying iodide surface coverage and a fixed 2-NAT surface coverage of 1/4 ML. Silver: Au surface atom, black: C atom, white: H atom, orange: chemisorbed S atom, and purple: I atom. Note that the side views are shown in **Extended Data Fig. 1**.

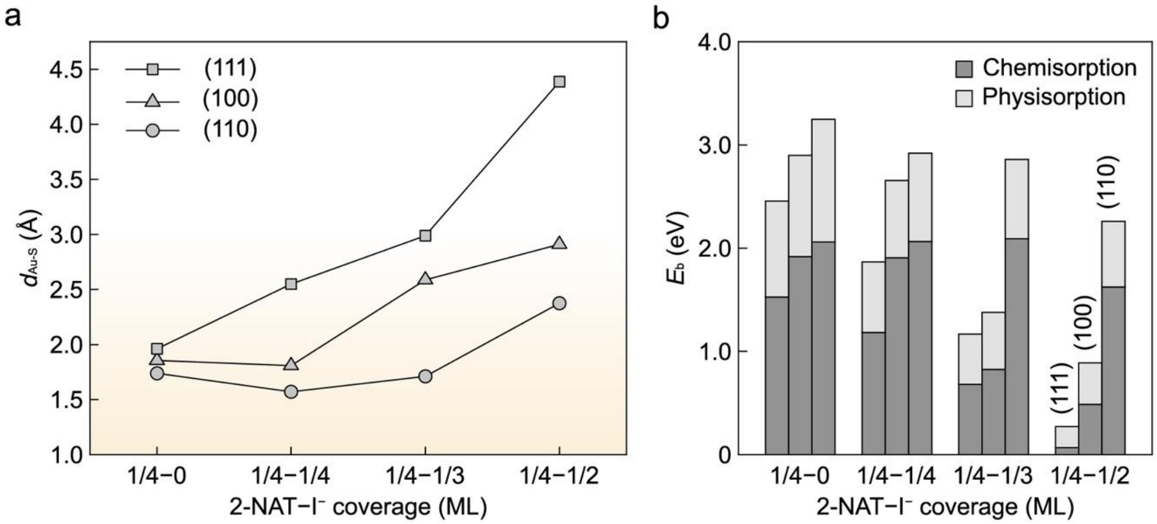

Supplementary Fig. 23. Effect of iodide surface coverage at a 1/4 ML of 2-NAT surface coverage, predicted by DFT. (**a**) The average of all Au–S distance ($d_{\mathrm{Au-S}}$) and (**b**) contributions to binding energy (E_{b}) of 2-NAT to each Au facet from chemisorption and physisorption for a fixed 2-NAT coverage of 1/4 ML and for increasing iodide coverages from left to right. Note that the trends of $d_{\mathrm{Au-S}}$ and E_{b} change upon increasing iodide coverage are consistent with those at a 1/3ML of 2-NAT shown in **Fig. 2b**,**c**.

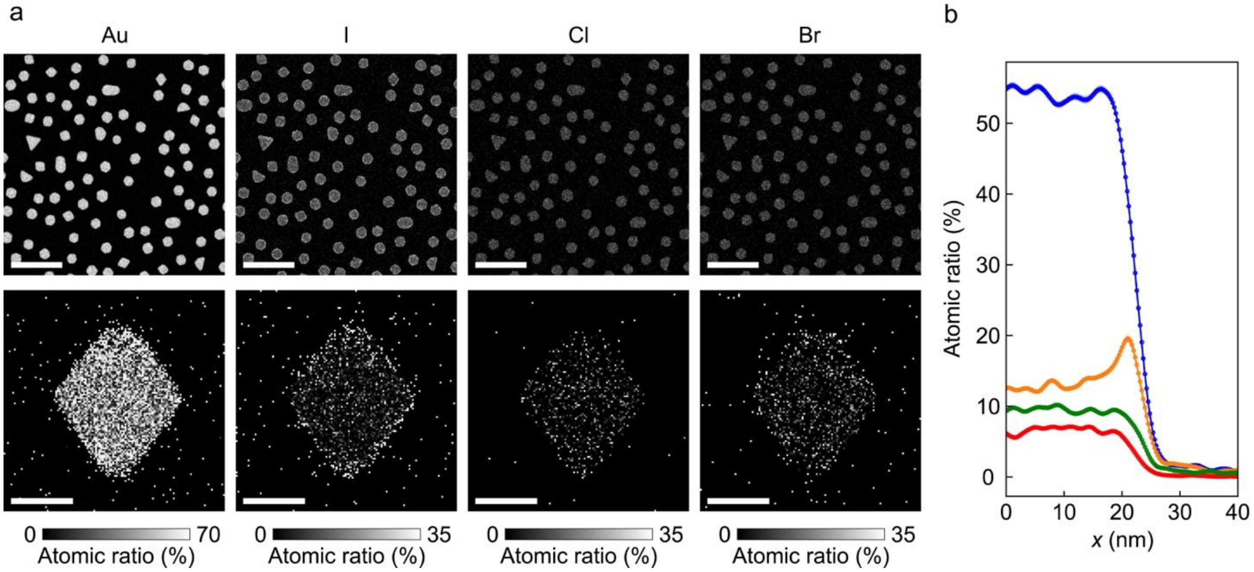

Supplementary Fig. 24. Scanning transmission electron microscopy-energy-dispersive X-ray spectroscopy (STEM-EDX) analysis for iodide-masked octahedra. (**a**) Low-magnification (top) and high-magnification (bottom) STEM-EDX mapping of gold, iodine, chlorine, and bromine. (**b**) The corresponding averaged atomic ratio line profiles (a total of 400 lines) of gold (blue), iodine (orange), chlorine (green), and bromine (red). The corresponding shades indicate the standard error of the mean. STEM-EDX analysis demonstrates a robust surface adsorption of iodide on gold NP, as captured by localized projection on the NP surfaces. In comparison, chloride and bromide are not necessarily adsorbed on the NP surfaces and are potentially removed during extensive washing, even though they present during the synthesis procedures. The masking process is performed at [I^-] of 16.4 μM. The analysis procedures and summarized data are depicted in **Extended Data Fig. 2**. Scale bars: 500 nm (top) and 50 nm (bottom).

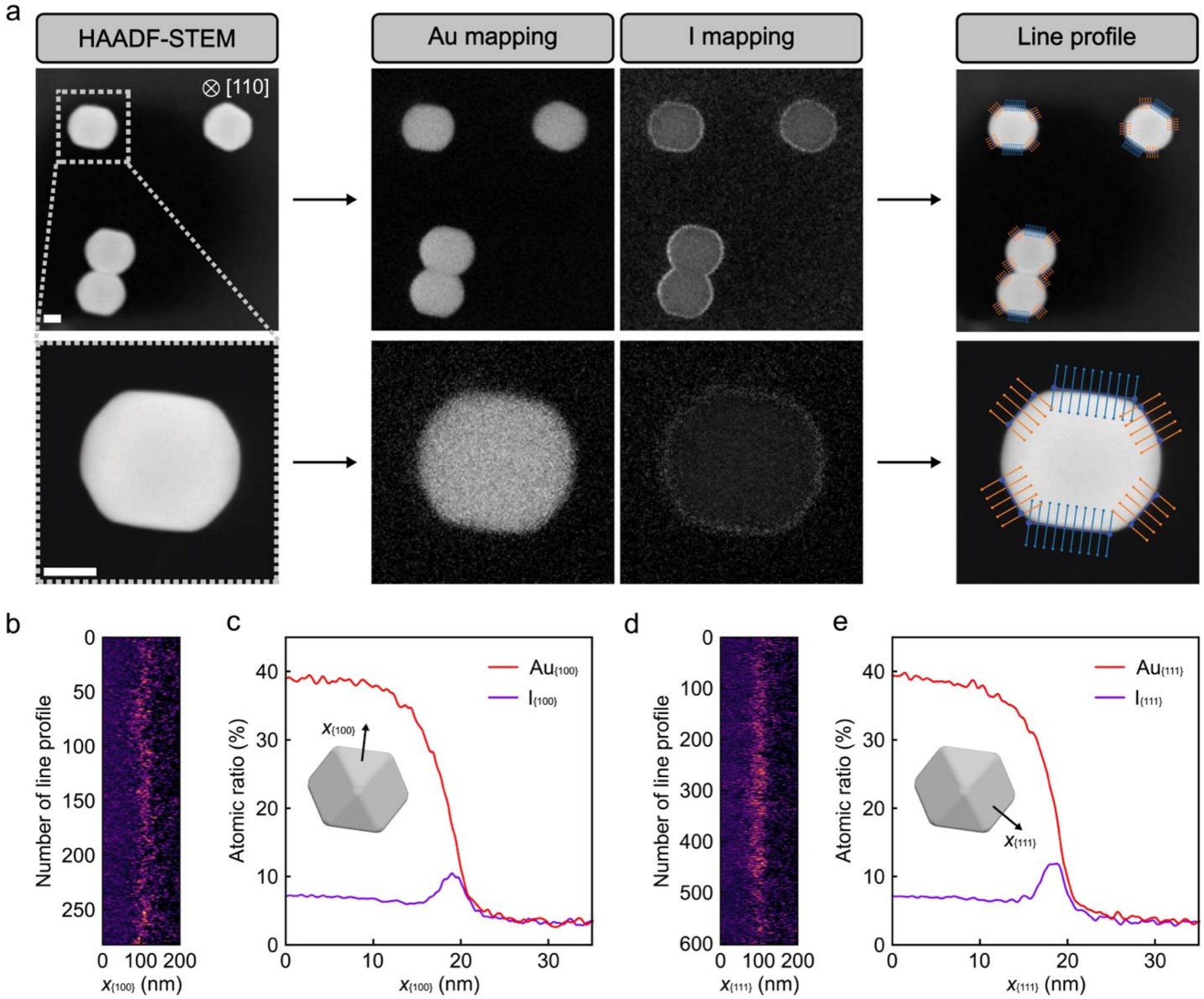

Supplementary Fig. 25. STEM-EDX analysis of iodide-masked cuboctahedra. (**a**) The workflow of iodide-masked cuboctahedra STEM-EDX analysis. (**b**,**c**) Stacked line profile of iodine based on the EDX mapping (**b**) and averaged atomic ratio profiles (lines) and standard error of the mean (shades) of gold and iodine (**c**) along the [100] direction. (**d**,**e**) Stacked line profile of iodine based on the EDX mapping (**d**) and averaged atomic ratio profiles (lines) and standard error of the mean (shades) of gold and iodine (**e**) along the [111] direction. Stacked line profiles in [100] and [111] directions are obtained along the contours of the projected cuboctahedra, as illustrated by the line profile step colored blue and orange, respectively in (**a**). For detailed experimental procedures, see **Methods**. Additional STEM-EDX mappings on iodide-masked cuboctahedra are shown in **Extended Data Fig. 3**. Scale bars: 20 nm.

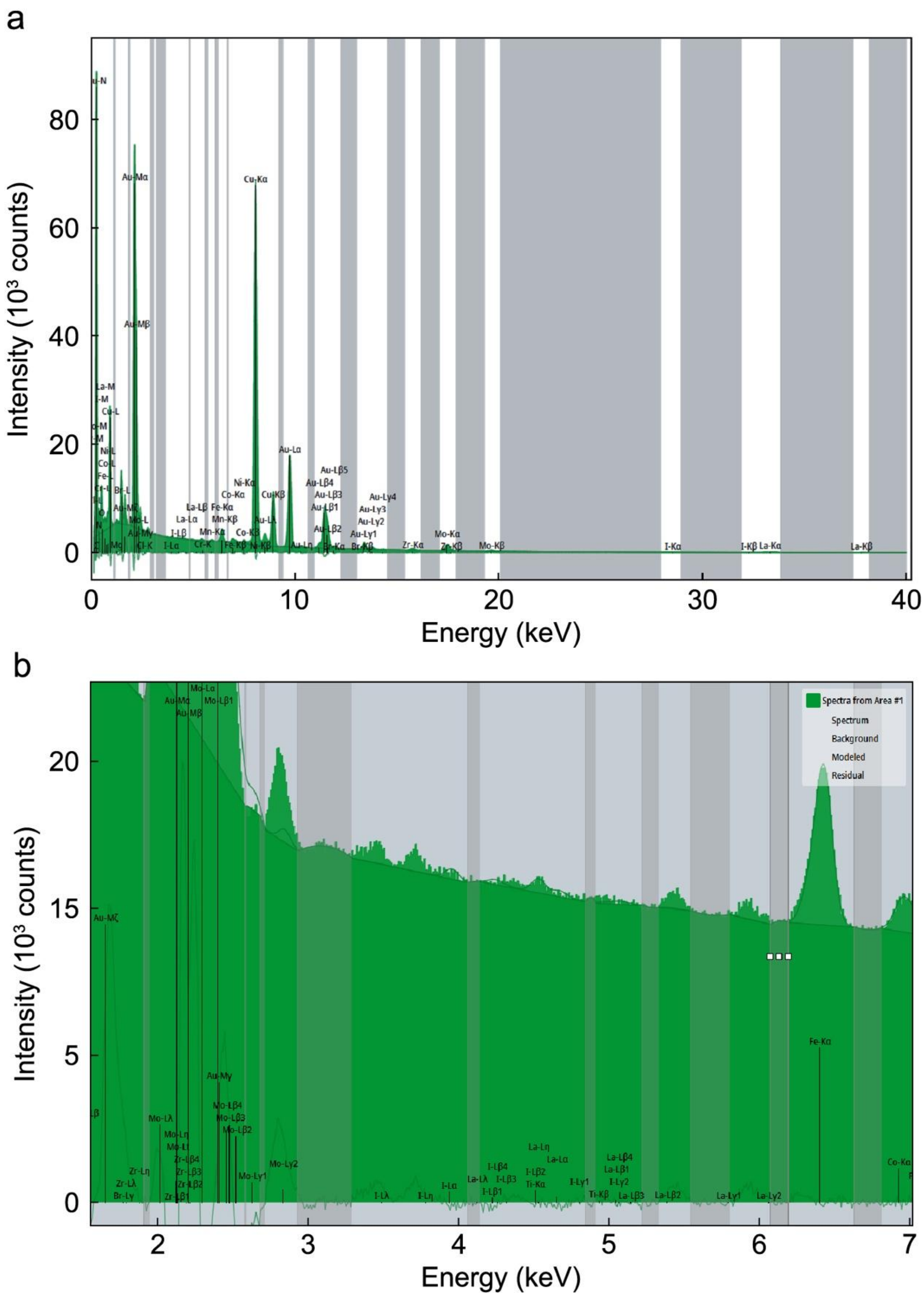

Supplementary Fig. 26. STEM-EDX characterization of iodide-masked NPs. (continued on the next page)

c

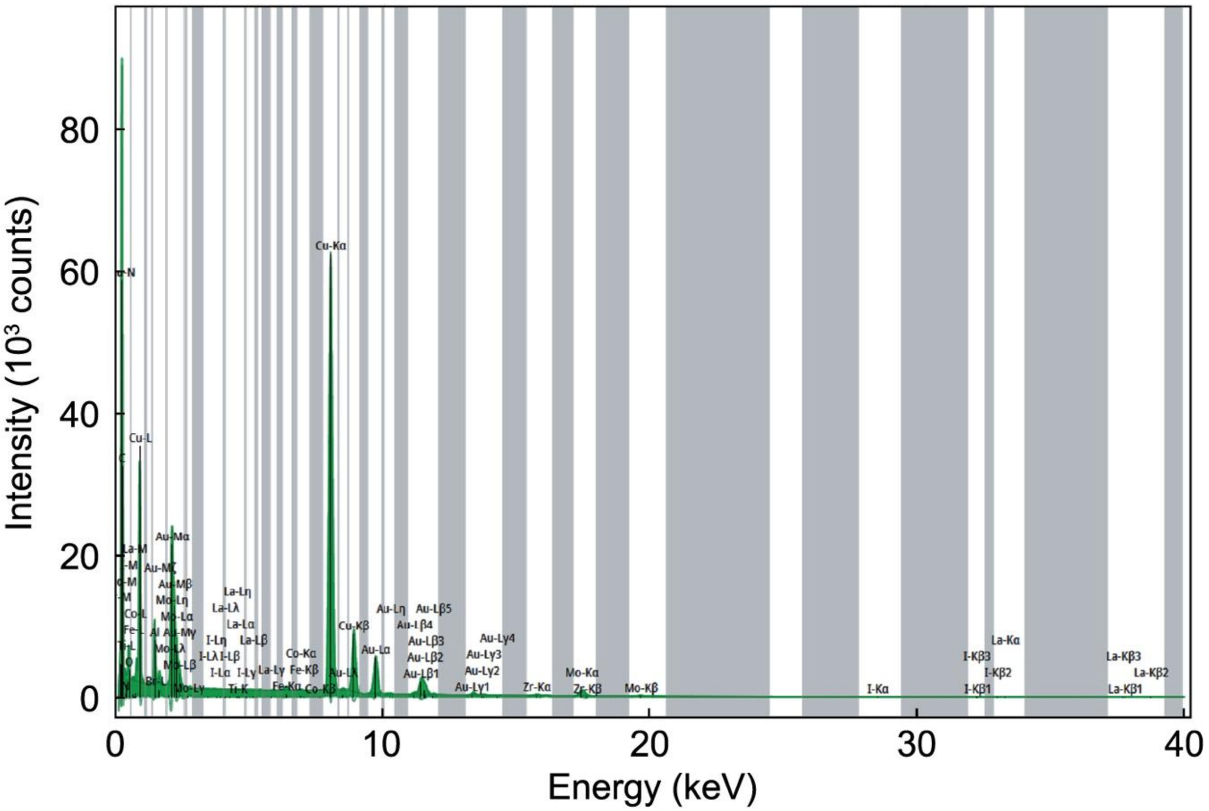

d

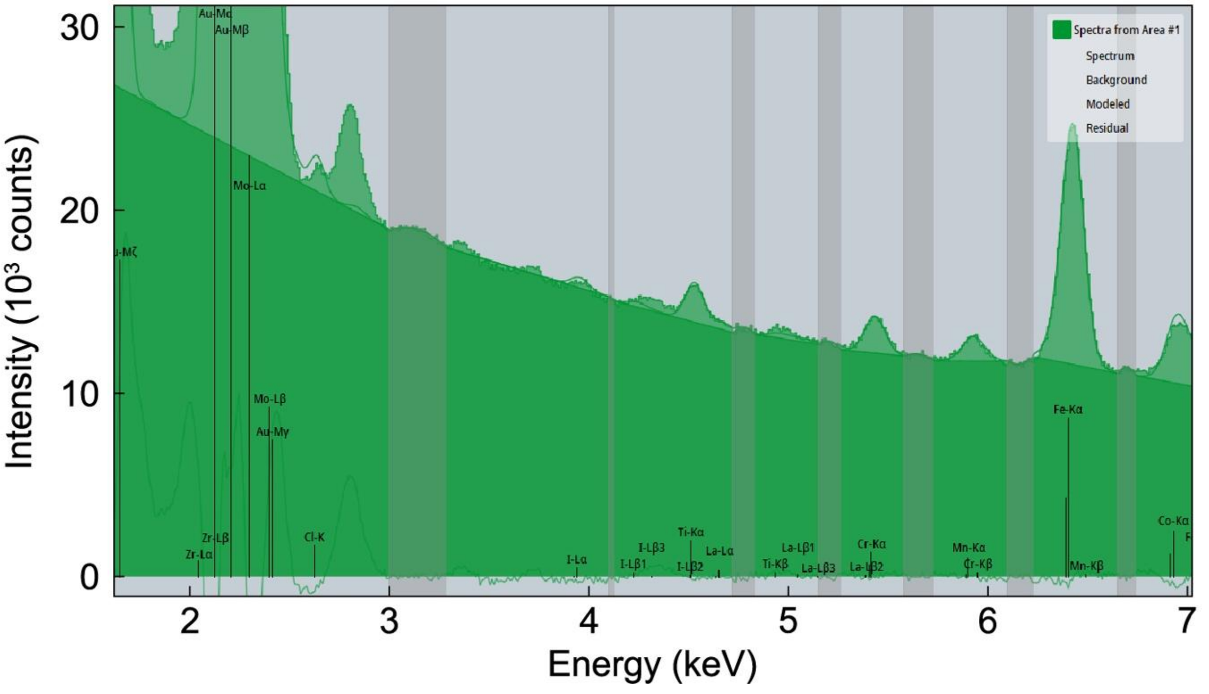

Supplementary Fig. 26. STEM-EDX characterization of iodide-masked NPs (continued). (**a−d**) EDX spectra of iodide-masked octahedra (**a,b**) and cuboctahedra (**c,d**), along with zoomed-in spectra of the iodine peaks. The masking process is performed at [I⁻] of 16.4 μM and 1.19 μM for octahedra and cuboctahedra, respectively. For detailed experimental procedures, see **Methods**.

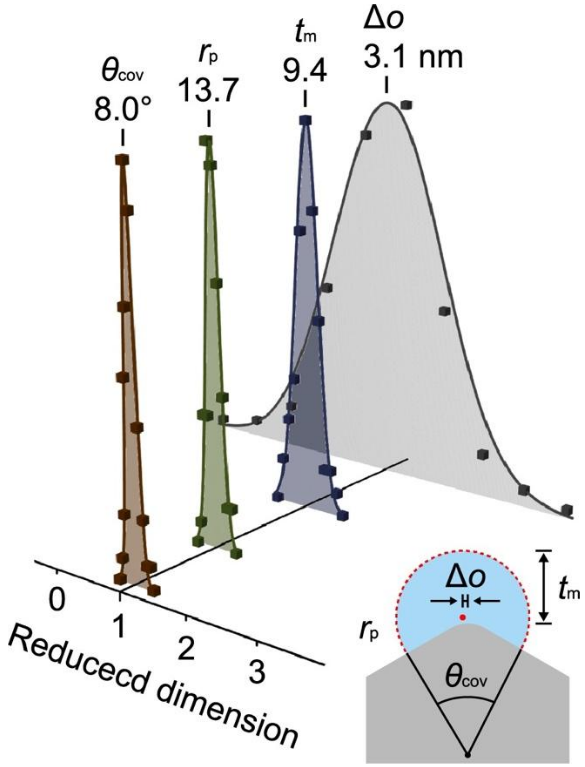

Supplementary Fig. 27. Patch shape analysis of representative patchy octahedra using machine-learning based TEM image analysis. Histograms of the patch shape parameters normalized by the average value of each parameter: coverage angle θ_{cov} of the patch projection on the NP contour (8.0 ± 0.4 °), fitted inscribed circle (red dotted circle) of the patch contour r_p (13.7 ± 0.8 nm), maximum patch thickness t_m (9.4 ± 1.0 nm), and offset of the patch center Δo (3.10 ± 1.6 nm) from the line connecting the octahedron center and the tip center, where the patch center is noted as the red dot, defined as the centroid of the fitted inscribed circle of the patch contour. The averaged values are noted on top of the histogram. A total of 71 patchy NPs is analyzed from the same sample as shown in **Fig. 3c** and **Supplementary Fig. 3**.

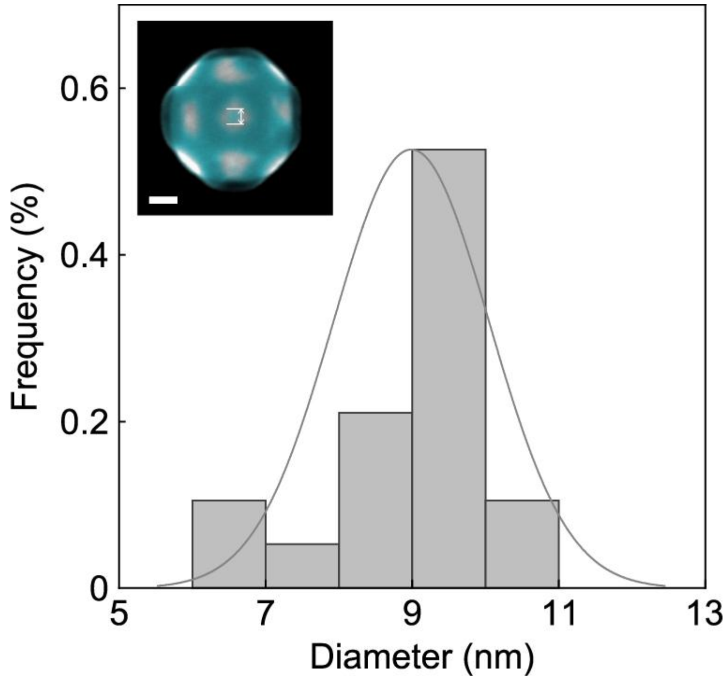

Supplementary Fig. 28. Histogram of the dot patch diameter on web-like patchy large rhombic dodecahedra. All twelve patches on the {110} faces are merged on the edges, and small dot patches are on the six {100} vertices, while the eight {111} truncated vertices remain patch-free. The diameter of the dot patches on the {100} vertices is 9.0 ± 1.0 nm. The patchy large rhombic dodecahedra are synthesized with 8.26 μM of [I^-] and 113.47 μM of [2-NAT]. See **Supplementary Note 2.6** and **Supplementary Table 7** for the detailed synthesis procedure and conditions. A total of 19 patches is measured. Scale bar: 20 nm.

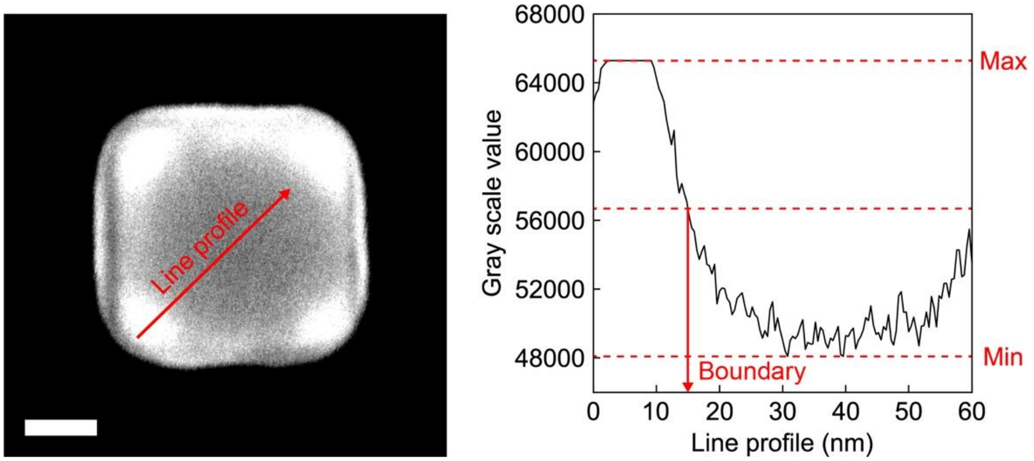

Supplementary Fig. 29. Patch patterning resolution analysis. Representative SEM image of a web-like patchy cube used for patch patterning resolution analysis (left). An example of how the boundary of the patch is defined as the position showing the half gray scale value between the maximum and minimum of the line profile (right). The average distance from a fully exposed gold region on a truncated vertex to the patch boundary is 5.7 ± 0.9 nm. A total of 23 NPs in SEM images is analyzed. Scale bar: 20 nm.

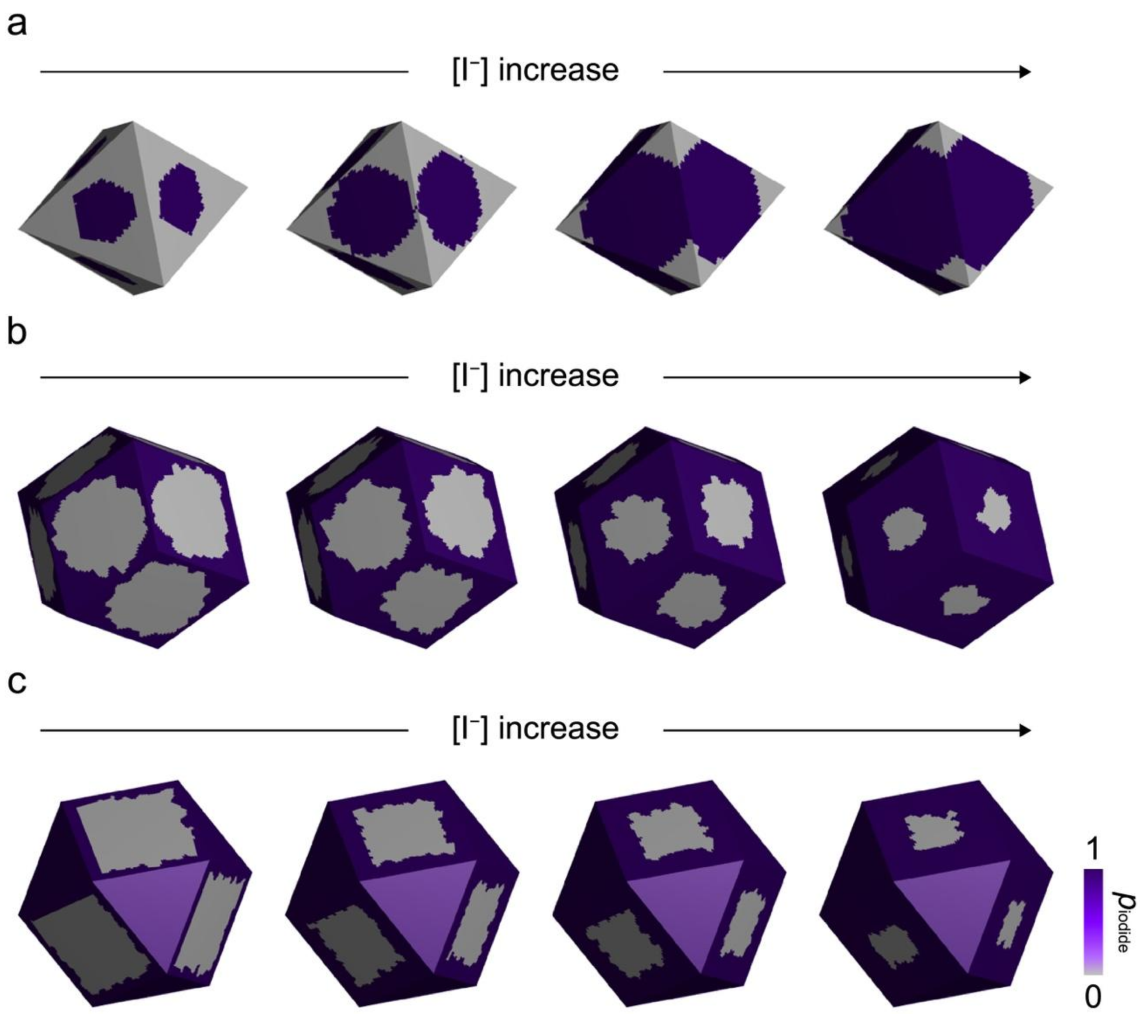

Supplementary Fig. 30. Theoretical prediction of iodide-masking on NPs. (**a**−**c**) Theoretical models of iodide-dominated probability p_{iodide} show the gradual expansion of iodide-masking dominated region on octahedra (**a**), rhombic dodecahedra (**b**), and cuboctahedra (**c**). In cuboctahedra, the iodide-masked region is initially formed on {111} faces and gradually expands to {100} faces.

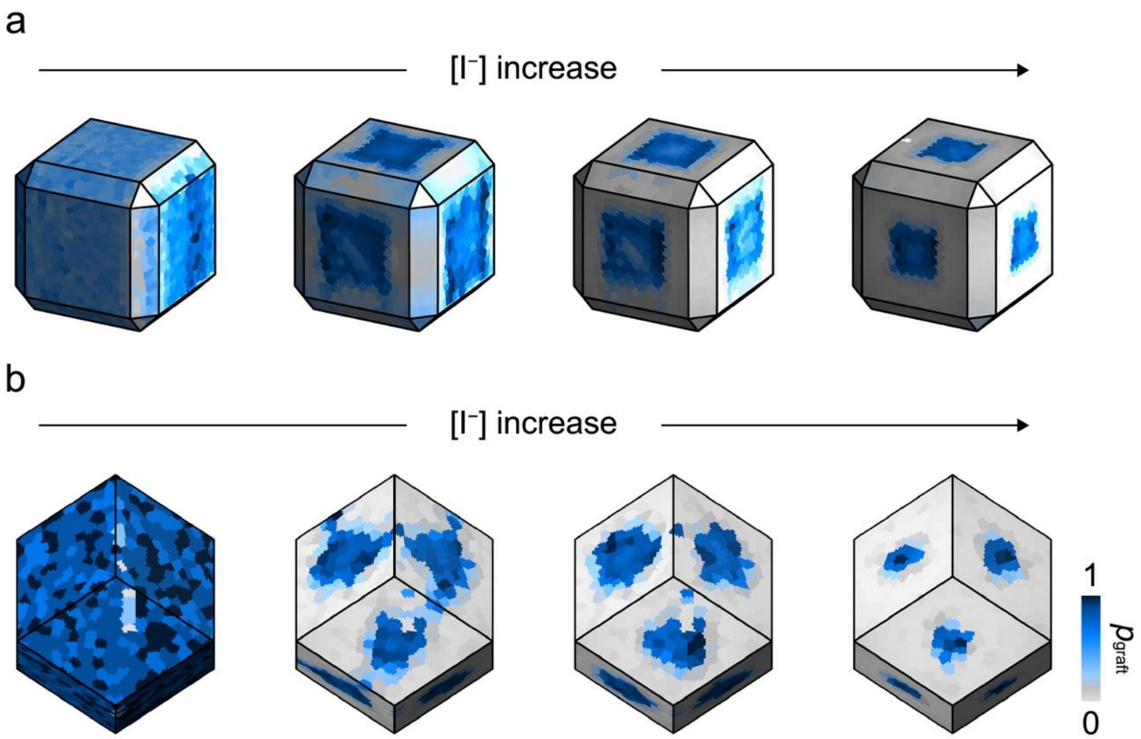

Supplementary Fig. 31. Theoretical prediction of polymer grafting on NPs with increasing iodide concentration. (**a,b**) Theoretical models of polymer chain grafting probability p_{graft} demonstrate the gradual patchy pattern transition from fully coated NPs to discrete patches NPs via web-like structures in cube (**a**) and rhombic dodecahedron (**b**) systems.

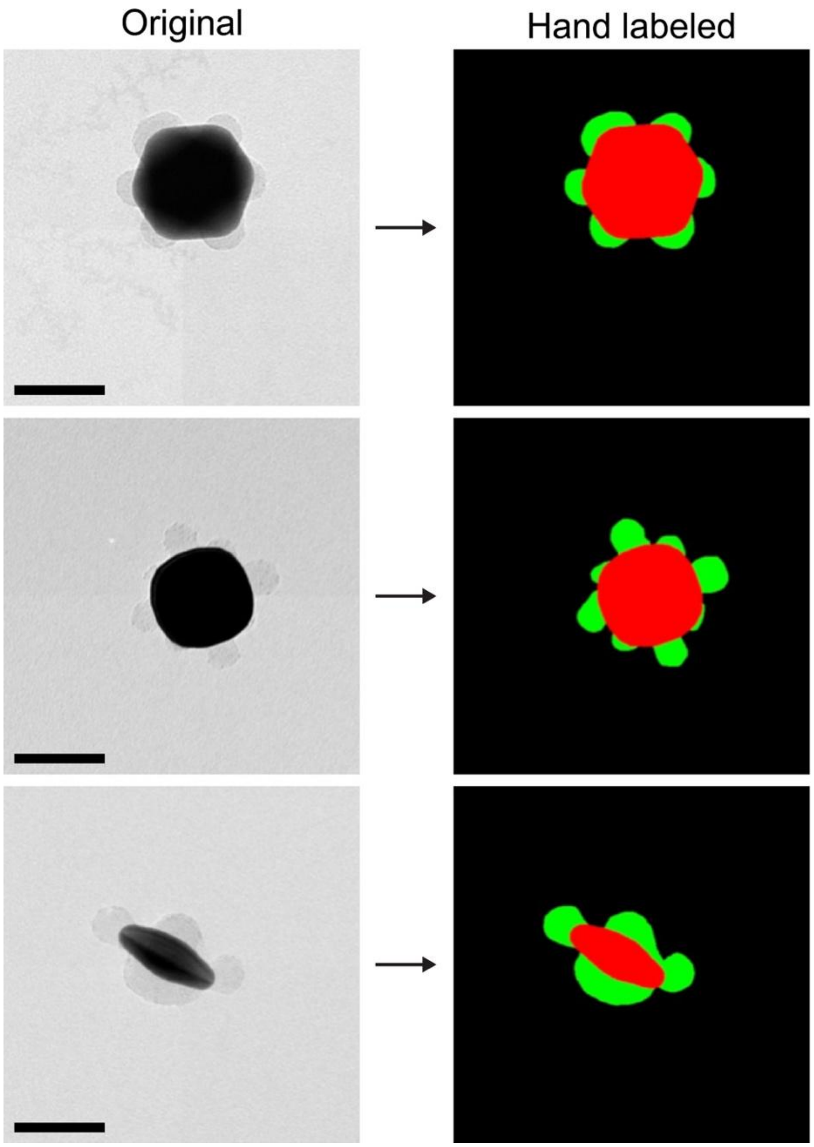

Supplementary Fig. 32. Training data for the ML-based TEM image segmentation. Representative examples of hand-labeled ground truth for octahedron (top), rhombic dodecahedron (middle), and bipyramid (bottom). The images are segmented into three regions: patch (green), NP (red), and background (black). For detailed procedures, see **Supplementary Note 3.3**. Scale bars: 50 nm.

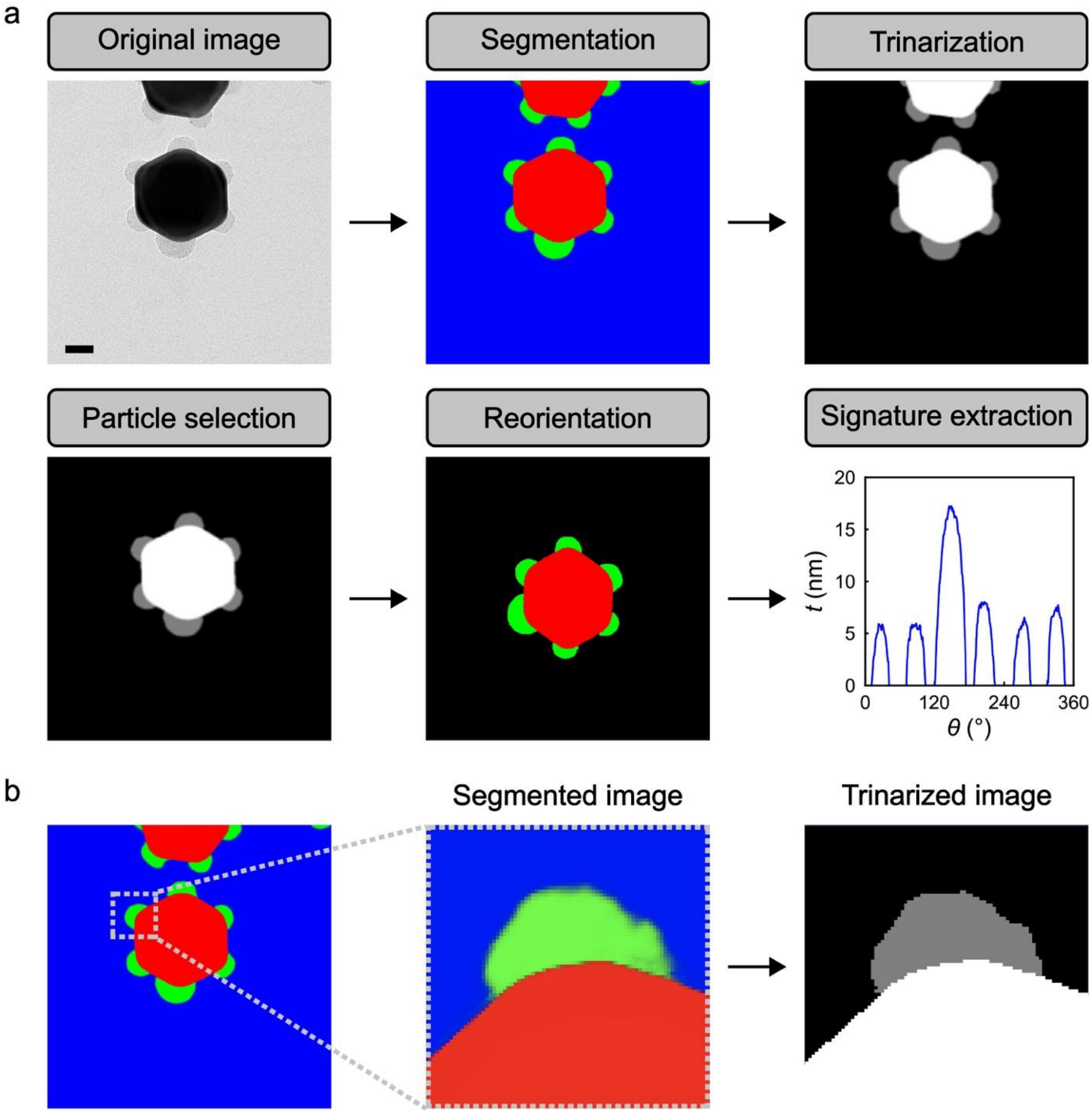

Supplementary Fig. 33. Machine learning-based segmentation and shape fingerprint analysis. (**a**) The workflow for machine learning-based segmentation of patchy NP from TEM images and shape fingerprint extraction with an example of a patchy octahedron. (**b**) The detailed example of image trinarization for enhancing edge definition from a segmented image. For detailed procedures, see **Supplementary Note 3.4**. The definition of shape fingerprint t is denoted in **Supplementary Figs. 34–36** for patchy octahedron, rhombic dodecahedron, and bipyramid systems, respectively. Scale bar: 20 nm.

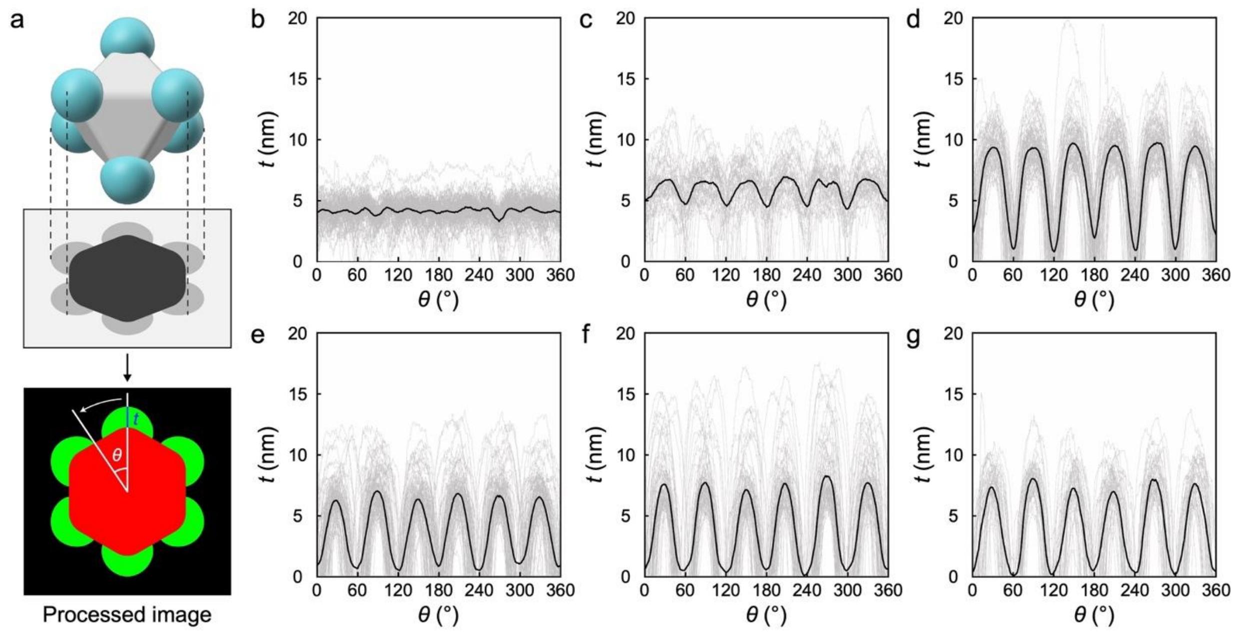

Supplementary Fig. 34. Shape fingerprint analysis of patchy octahedra. (**a**) Schematic of shape fingerprint analysis for a patchy octahedron. (**b–g**) The shape fingerprint t at each angle θ analyzed from the shape fingerprint of patchy octahedra synthesized with 0 (**b**), 0.17 (**c**), 0.42 (**d**), 0.83 (**e**), 16.4 (**f**), and 117.6 μM (**g**) of [I⁻]. See **Supplementary Table 2** for the synthesis conditions.

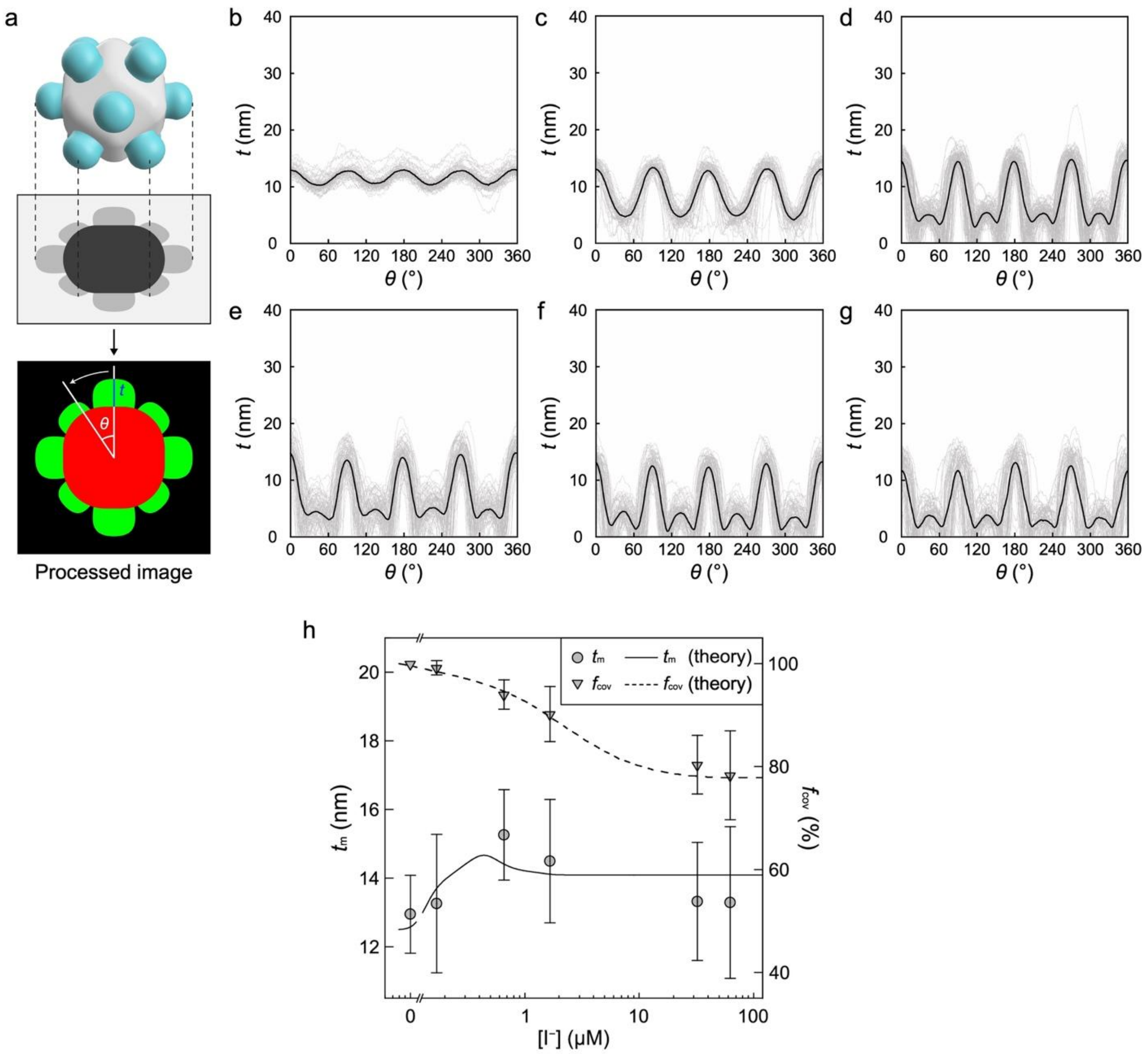

Supplementary Fig. 35. Shape fingerprint analysis of patchy small rhombic dodecahedra. (**a**) Schematic of shape fingerprint analysis for patchy rhombic dodecahedra. (**b–g**) The shape fingerprint t at each angle θ analyzed from the shape fingerprint of patchy rhombic dodecahedra synthesized with 0 (**b**), 0.17 (**c**), 0.67 (**d**), 1.66 (**e**), 32.3 (**f**), and 62.5 μM (**g**) of [I⁻]. See **Supplementary Table 8** for the synthesis conditions. (**h**) Maximum patch thickness t_{m} and patch coverage fraction f_{cov} (defined as the fraction of NP surface covered by patches) from theory (solid and dashed lines) and experiment (mean values and standard deviation shown as symbols plotted with error bars). From low to high [I⁻], a total of 48, 57, 98, 79, 74, and 58 NPs is analyzed for each sample, respectively.

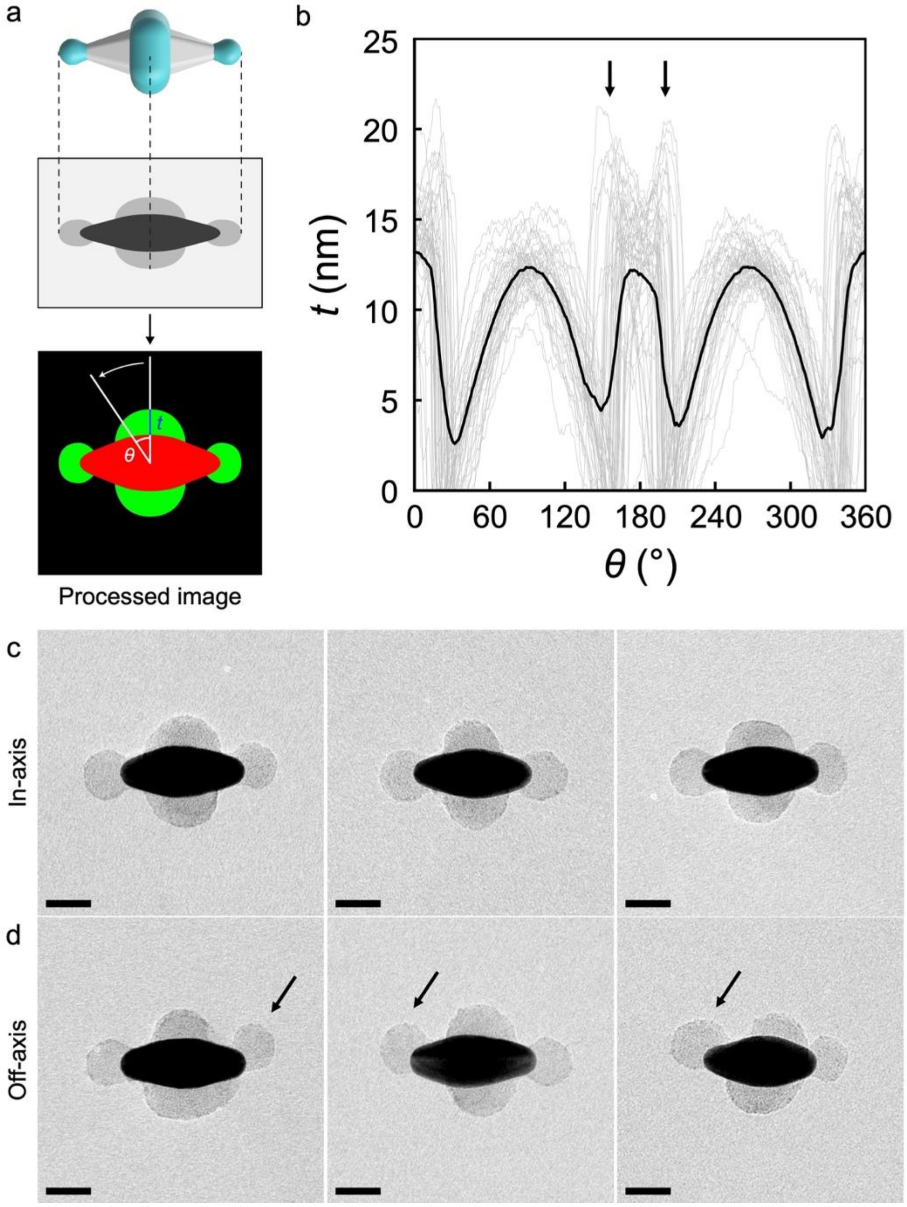

Supplementary Fig. 36. Shape fingerprint analysis of patchy bipyramids. (**a**) Schematic of shape fingerprint analysis for a patchy bipyramid. (**b**) The shape fingerprint t at each angle θ analyzed from the shape fingerprint of patchy bipyramids synthesized with 32.3 μM of [I^-]. A total of 47 images is analyzed. (**c**,**d**) Representative TEM images of patchy bipyramids with in-axis (**c**) and off-axis (**d**) tip-patched structures. For a detailed analysis description, see **Supplementary Note 3.4**. Multiple peaks, marked with arrows in (**b**), appear around θ = 180° in the shape fingerprint because the patches on the tips are occasionally positioned off-axis, presumably due to the sharp tips of bipyramids, as marked with arrows in (**d**). Scale bars: 20 nm.

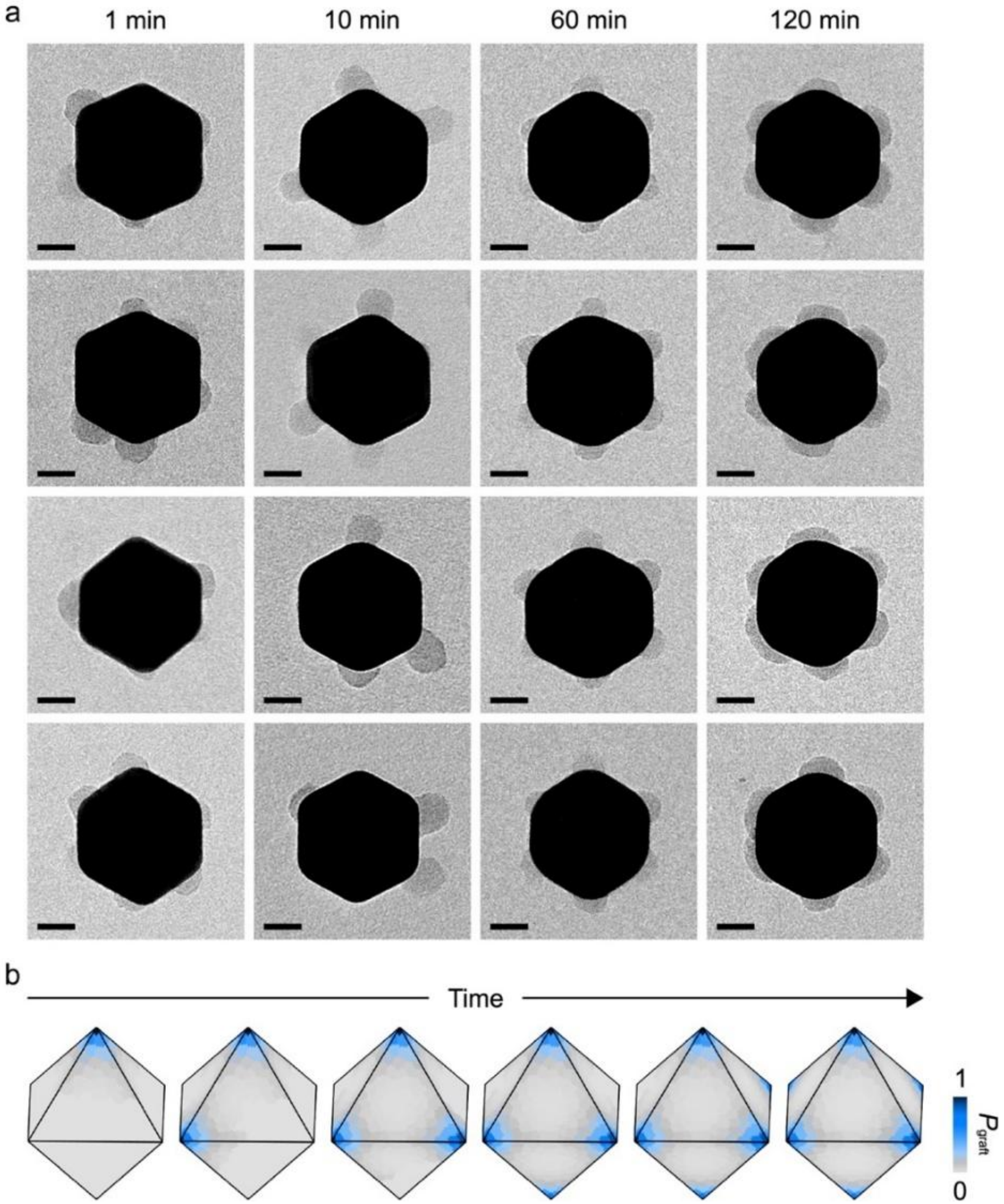

Supplementary Fig. 37. Temporal evolution of the patch formation on gold octahedra. (**a**) Representative TEM images of patchy octahedra at different time points after the polymer grafting starts. First, incomplete, symmetry-broken vertex patches are formed (1 and 10 min). Then small patches at all six vertices are observed at around 60 min. Subsequently, the patches gradually grow larger, and large vertex patches are observed at around 120 min. [I⁻] and [2-NAT] are fixed at 6.62 μM and 56.7 μM, respectively. (**b**) The evolution of grafting probability p_{graft} during patchy octahedra formation, obtained from the MC simulation of polymer chain grafting. Scale bars: 20 nm.

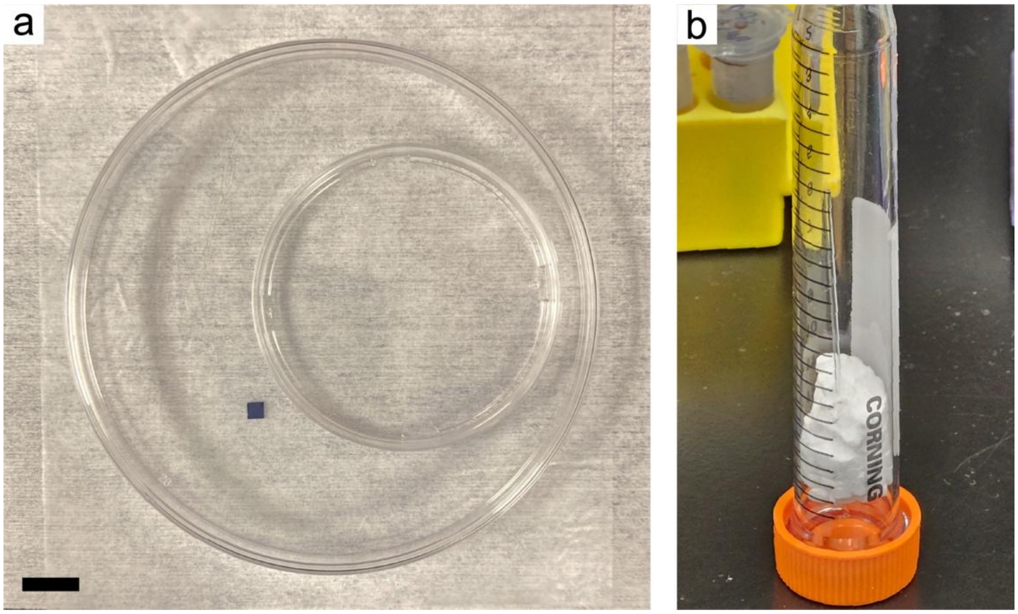

Supplementary Fig. 38. Photos of patchy NP self-assembly experiment setups. (**a**,**b**) The self-assembly experiments for patchy NPs are carried out using coffee-ring effect-driven (**a**) and capillary force-driven (**b**) methods. For detailed procedures, see **Methods**. Scale bar: 1 cm.

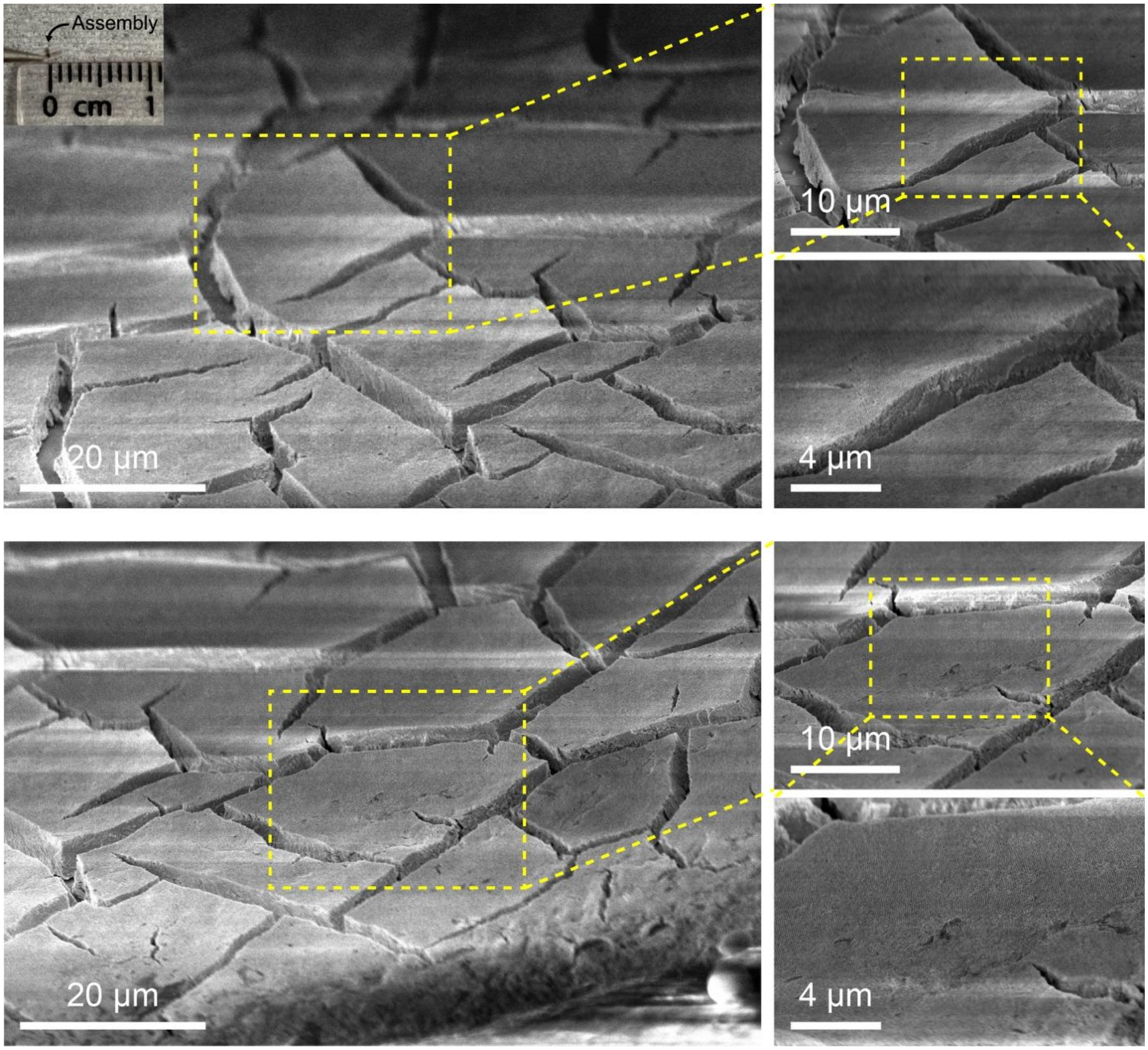

Supplementary Fig. 39. Large-scale self-assembly of patchy small rhombic dodecahedra in a glass tube, prepared using capillary force-driven method. Low magnification and zoomed-in SEM images of patchy rhombic dodecahedra self-assemblies. For the detailed description of self-assembly procedure, see **Methods**. The inset of the top left panel shows the cylindrical glass tube containing large-scale self-assembly structure. The zoomed-in views of NPs prior to assembly are shown in **Extended Data Fig. 7h–k**, and detailed synthesis condition for the presented sample is summarized in **Supplementary Table 8**.

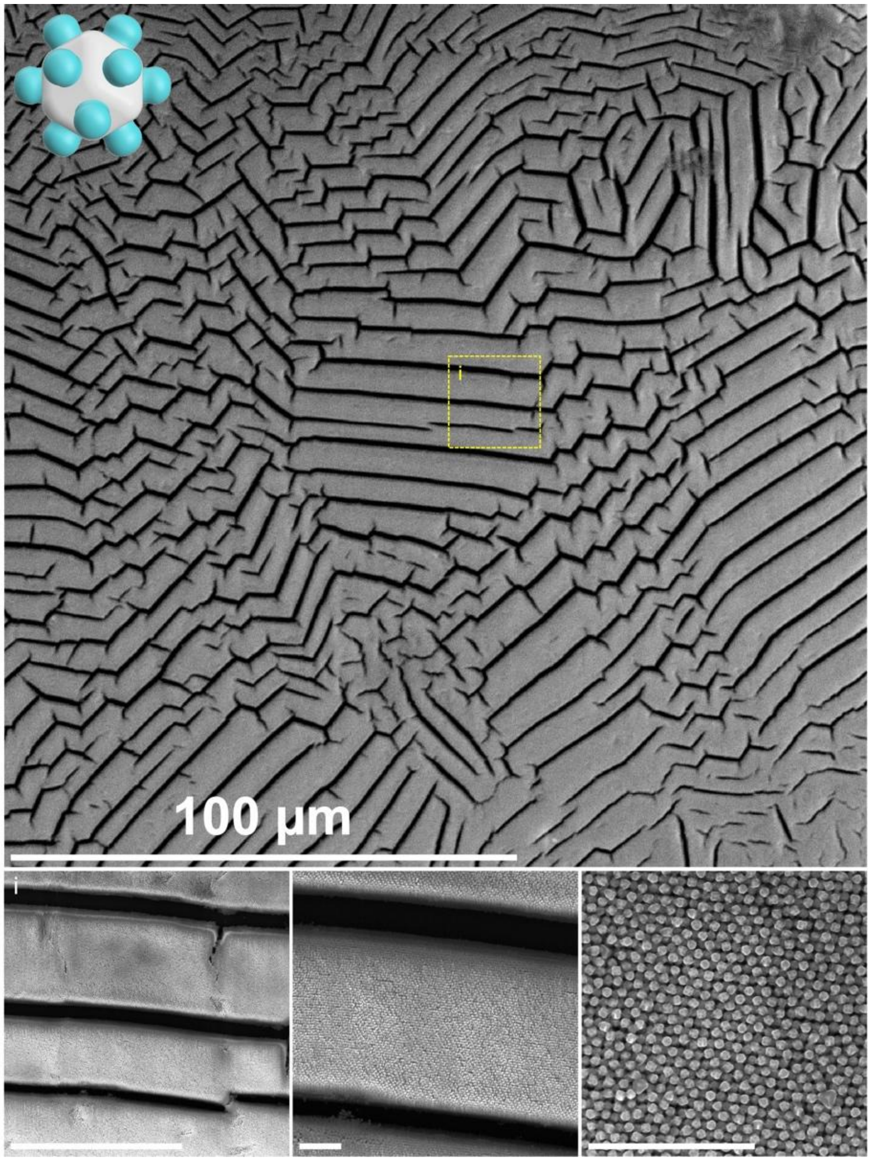

Supplementary Fig. 40. SEM images of large-scale patchy small rhombic dodecahedra self-assembly from coffee ring effect-driven process. Low magnification (top row) and zoomed-in view (bottom row) of patchy rhombic dodecahedra self-assembly. The bottom rows display increasingly magnified areas from left to right. The images of patchy NPs prior to assembly are shown in **Extended Data Fig. 7h–k**, and their detailed synthesis conditions are summarized in **Supplementary Table 8**. Scale bars for the bottom row: 10 μm (left) and 1 μm (middle and right).

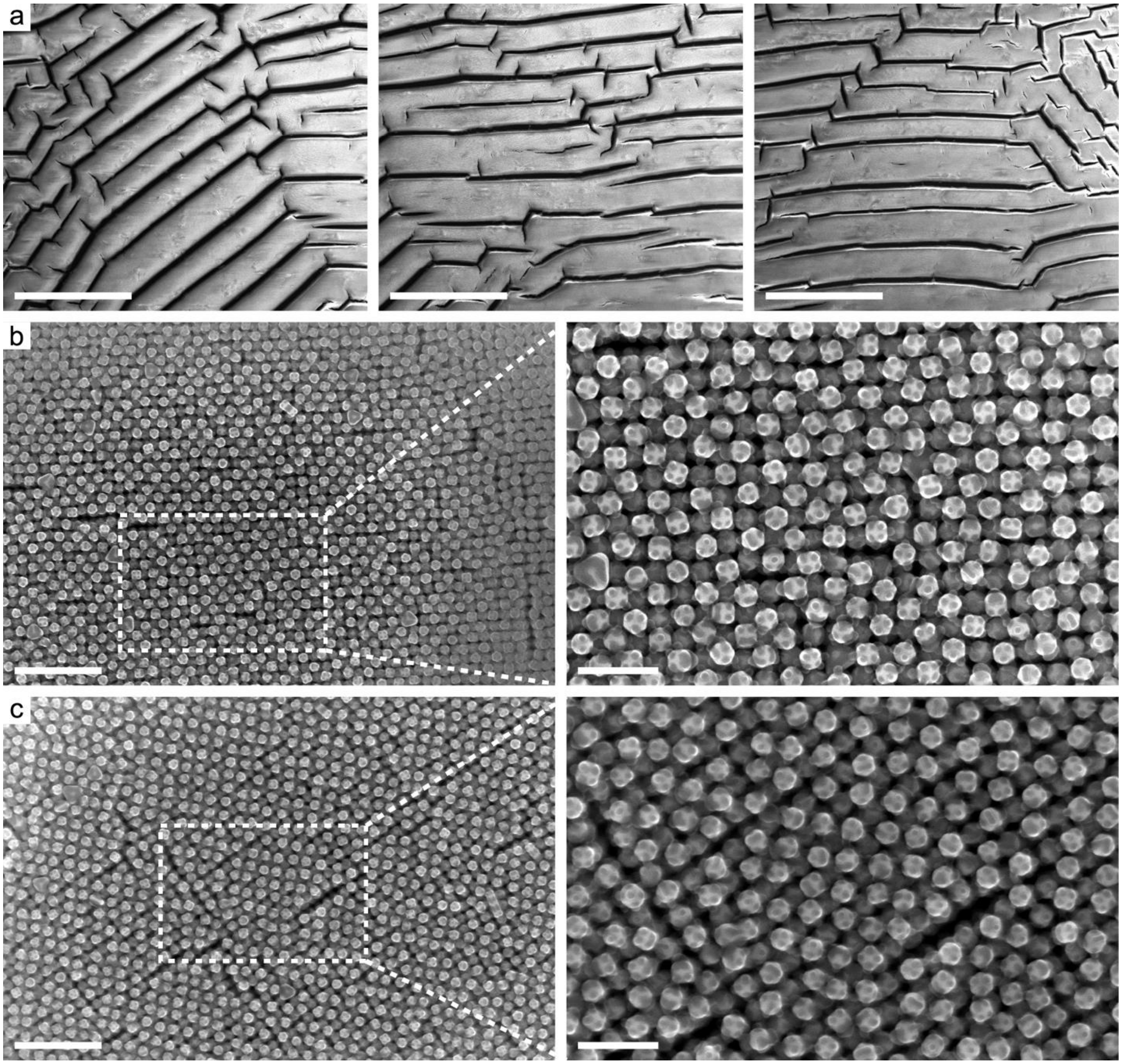

Supplementary Fig. 41. Detailed view of patchy small rhombic dodecahedra self-assembly. (**a**−**c**) Additional SEM images of the patchy rhombic dodecahedra self-assembly captured in low magnifications (**a**) and high magnifications (**b**,**c**). Note that (**b**) captures approximately the same region as in **Fig. 5e**, with slight reorientation. The images of patchy NPs prior to assembly are shown in **Extended Data Fig. 7h–k**, and their detailed synthesis conditions are summarized in **Supplementary Table 8**. Scale bars: (**a**) 20 μm, (**b**,**c**) 500 nm (left), and 200 nm (right).

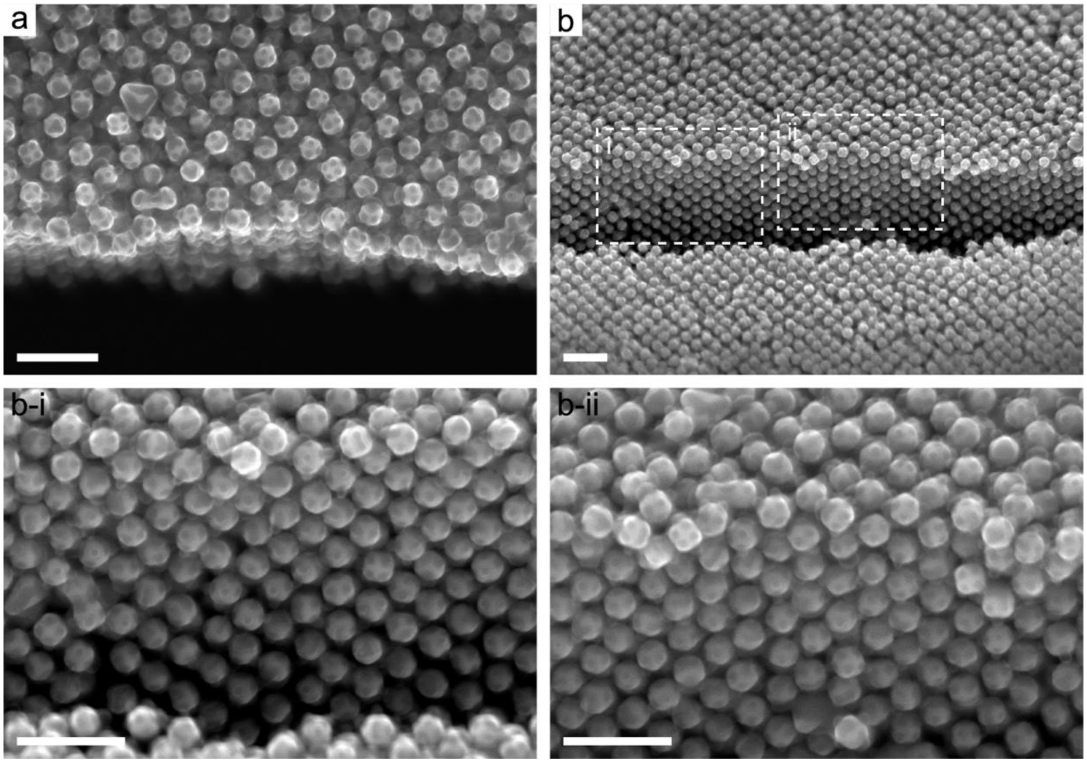

Supplementary Fig. 42. Side view of patchy small rhombic dodecahedra assembly. (**a**,**b**) SEM images of patchy small rhombic dodecahedra self-assembly from different viewing angles: top (**a**) and 45°-tilted (**b**) views of the same region as in the top view. The bottom row presents two zoomed-in areas from (**b**). Note that a portion of the **b-i** image is shown in **Fig. 5c**. Scale bars: 200 nm.

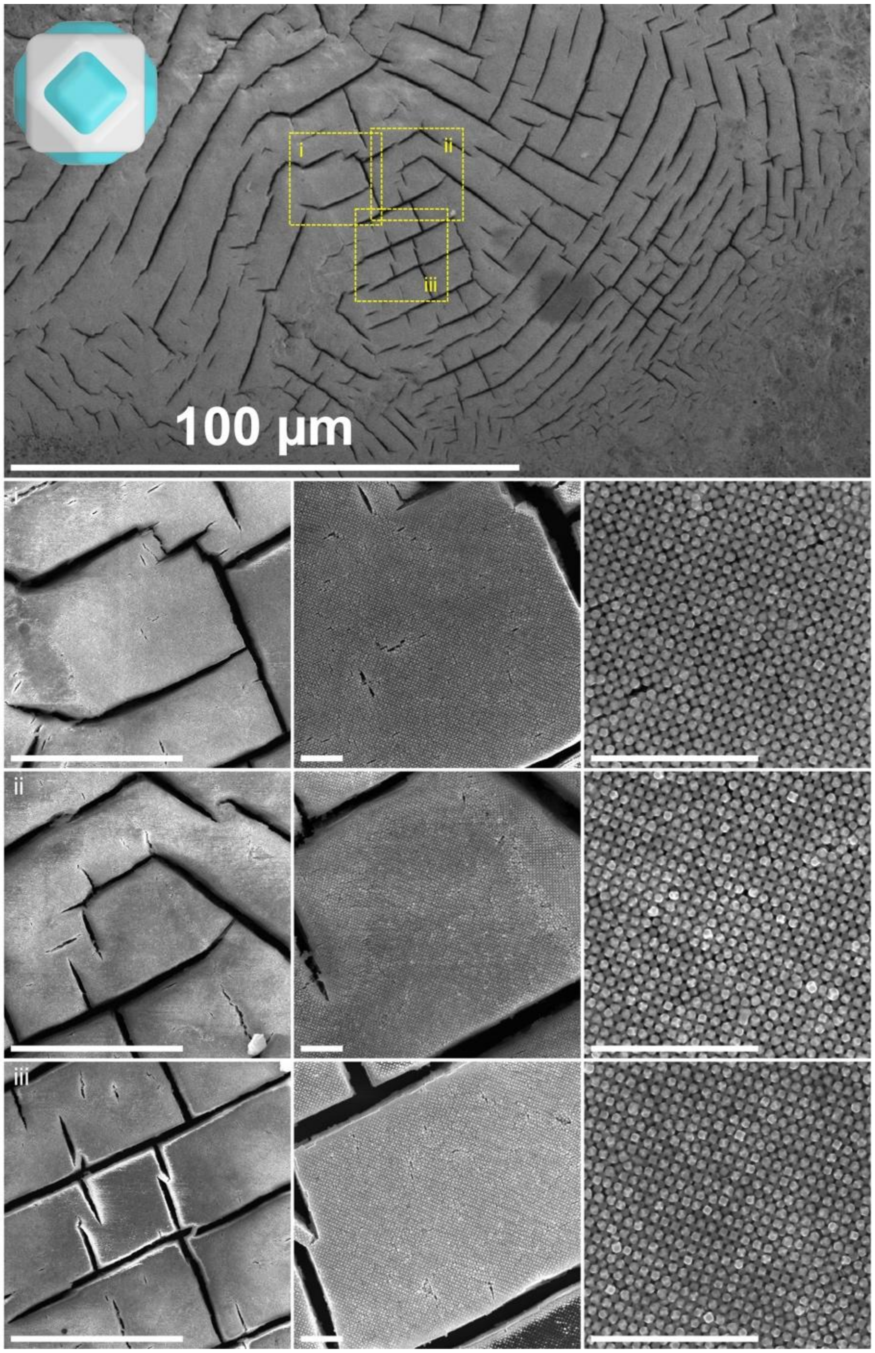

Supplementary Fig. 43. SEM images of large-scale face-patched cuboctahedra self-assembly. SEM images in low magnification (top row) and high magnification (bottom three rows) of face-patched cuboctahedra self-assembly. The bottom three rows show increasingly magnified areas from left to right. The zoomed-in views of face-patched cuboctahedra prior to assembly are shown in **Fig. 3i**, and their detailed synthesis conditions are summarized in **Supplementary Table 5**. Scale bars for the images other than the top one: 10 μm (left) and 1 μm (middle and right).

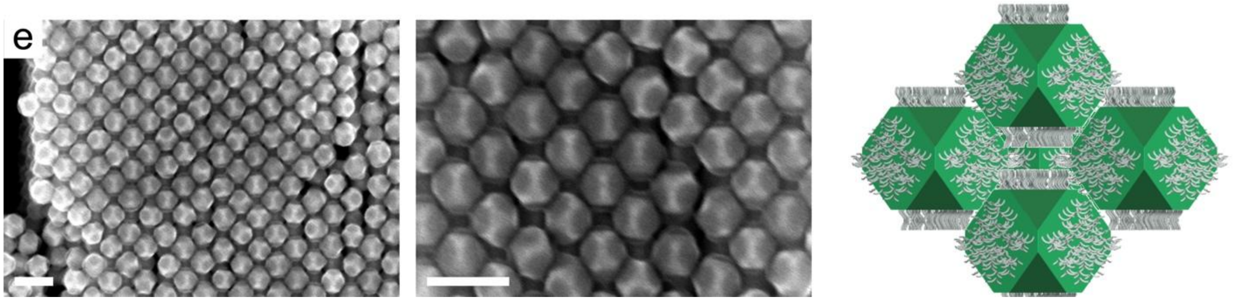

Supplementary Fig. 44. Detailed view of patchy cuboctahedra assembly. (**a–d**) Representative SEM (**a–c**) and TEM (**d**) images of self-assembly of patchy cuboctahedra with the (100) plane of the lattice exposed. (**e**) SEM image of the patchy cuboctahedra BCC assembly with the (110) plane of the lattice exposed, and the corresponding 3D model. The zoomed-in views of face-patched cuboctahedra prior to assembly are shown in **Fig. 3i**, and their detailed synthesis conditions are summarized in **Supplementary Table 5**. Scale bars: 100 nm.

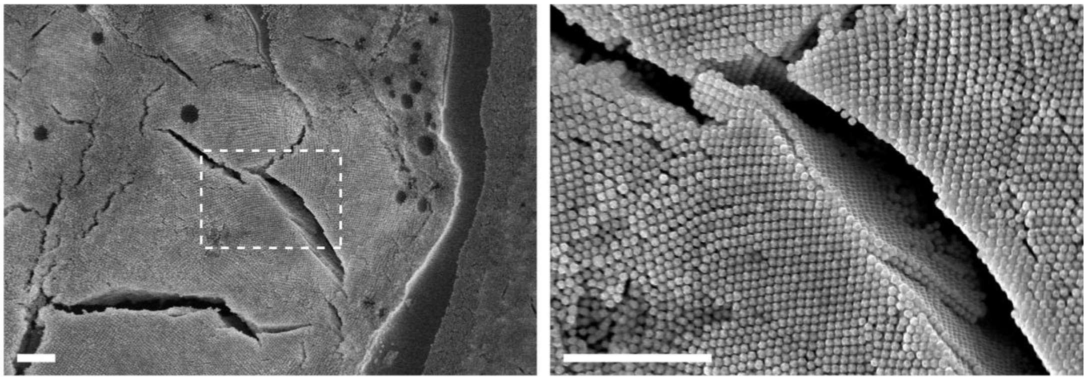

Supplementary Fig. 45. Assembly structure of patchy cuboctahedra at a crack showing multilayers. The zoomed-in views of face-patched cuboctahedra prior to assembly are shown in **Fig. 3i**, and their detailed synthesis conditions are summarized in **Supplementary Table 5**. Scale bars: 1 μm.

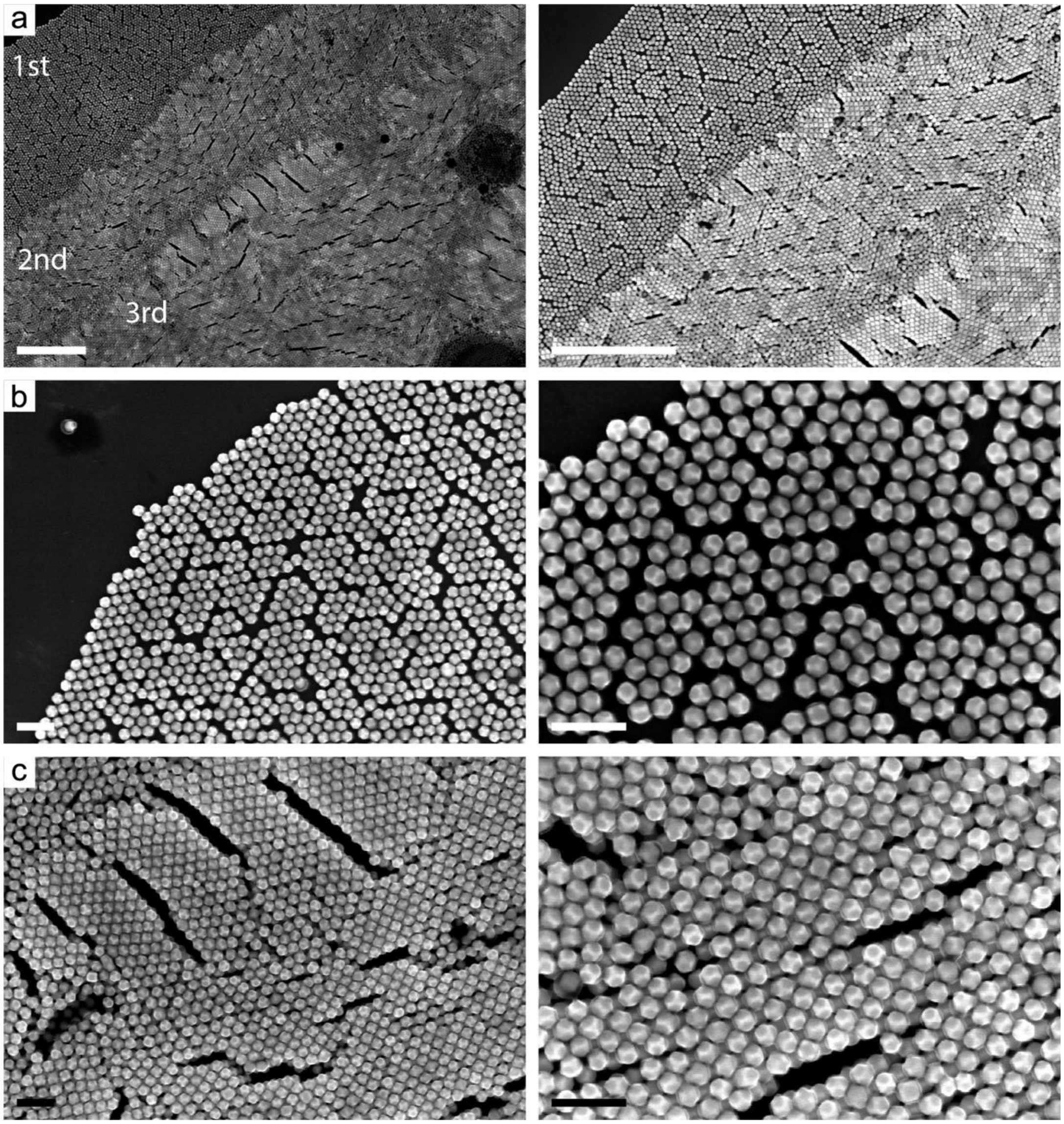

Supplementary Fig. 46. Effect of number of layers on patchy cuboctahedra assembly structure. (**a**) SEM images of a few-layered self-assembly of the patchy cuboctahedra. (**b**,**c**) Zoomed-in views of the first (**b**) and third (**c**) layers in the assembly. NPs form a pseudo close-packed structure in a single-layer assembly. In presence of additional layers, a BCC-like lattice structure appears with the (100) plane of the lattice exposed, consistent with what is shown in **Supplementary Fig. 44**. The zoomed-in views of face-patched cuboctahedra prior to assembly are shown in **Fig. 3i**, and their detailed synthesis conditions are summarized in **Supplementary Table 5**. Scale bars: (**a**) 1 μm and (**b**,**c**) 200 nm.

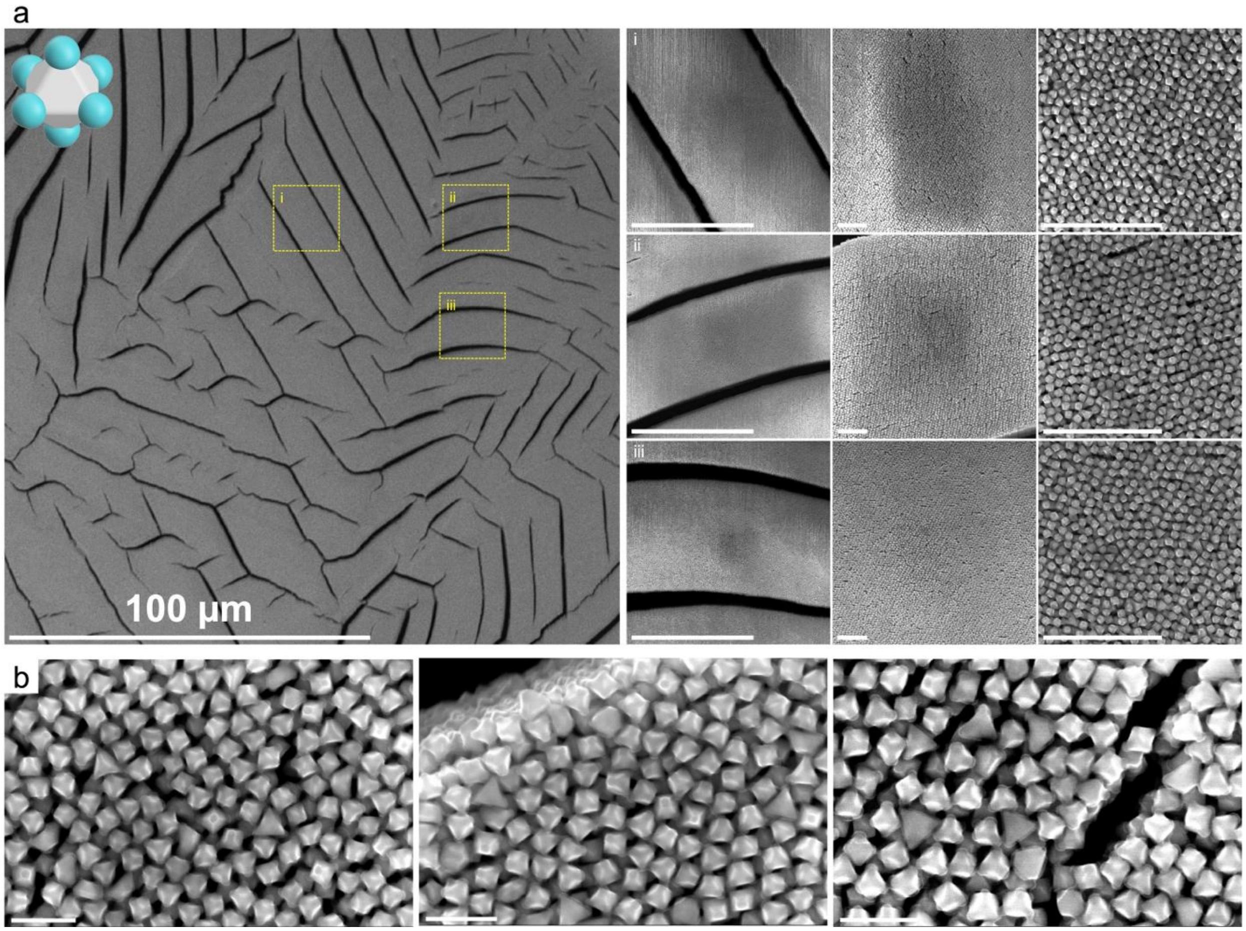

Supplementary Fig. 47. SEM images of large-scale patchy octahedra self-assembly. (**a**) SEM images at low magnification (left) and high magnifications (three right columns) of patchy octahedra self-assembly. The right columns show increasingly magnified areas from left to right. (**b**) High-magnification SEM images of the assembly structures, showing detailed orientations of each patchy NP. The zoomed-in views of patchy octahedra prior to assembly are shown in **Fig. 3c**, and their detailed synthesis conditions are summarized in **Supplementary Table 2**. Scale bars: (**a**) 10 µm (left), 1 µm (middle and right), and (**b**) 200 nm.

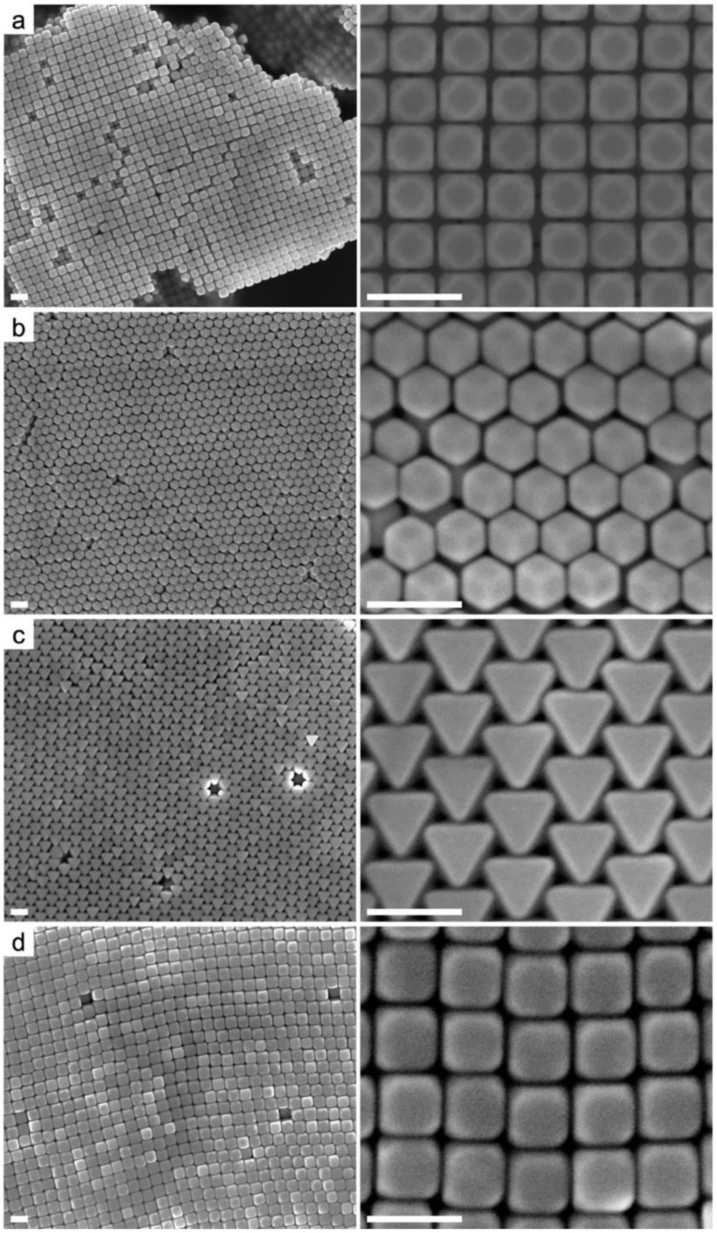

Supplementary Fig. 48. Self-assembly of pristine gold NPs with different shapes. (**a**–**d**) Low-magnification (left) and zoomed-in (right) SEM images of pristine cuboctahedra (**a**), rhombic dodecahedra (**b**), octahedra (**c**), and cubes (**d**). Scale bars: 100 nm.

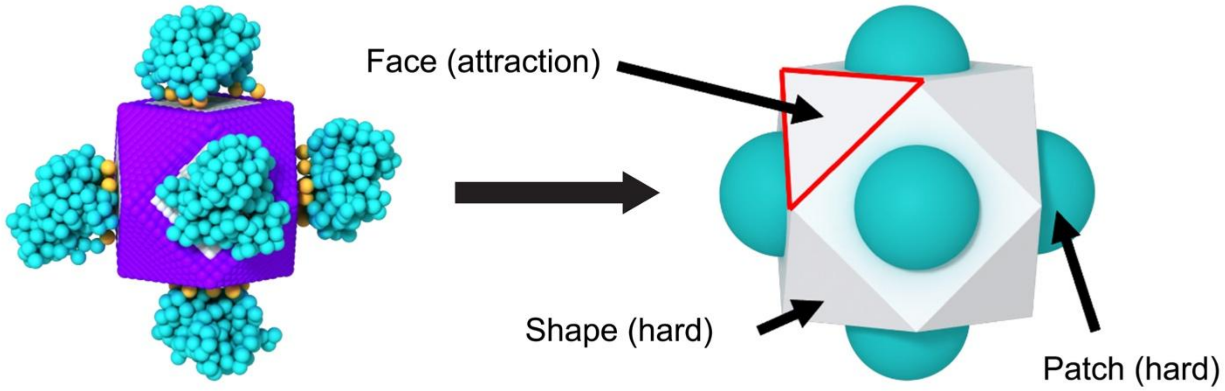

Supplementary Fig. 49. A single patchy NP model for MC simulations of self-assembly. The PAA−PAA steric repulsion is modeled by placing a hard hemisphere at each patch location. A directional and short-range interaction on each face not covered by patch is used to model the Au−Au van der Waals attraction between exposed sites. For the detailed procedure, see **Supplementary Note 9**.

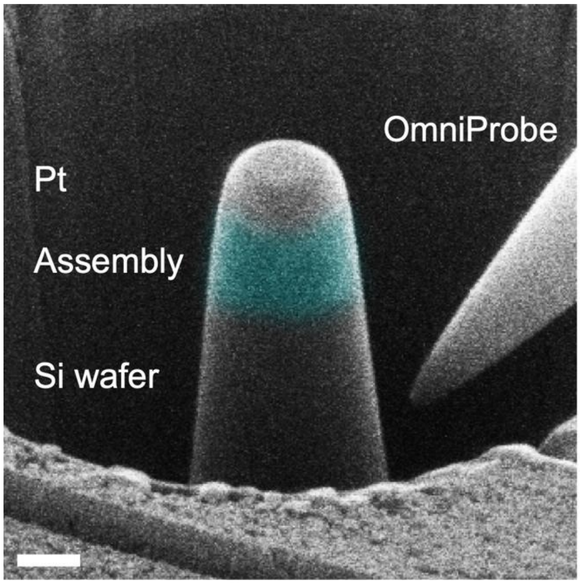

Supplementary Fig. 50. Sample preparation for X-ray tomography using focused ion beam (FIB). SEM image of the patchy rhombic dodecahedra self-assembly after FIB milling. The assembly is shaped into a cylinder with a diameter of ~2.5 μm for X-ray tomography. The assembly (false-colored in cyan) is prepared on a Si wafer, and Pt deposition is performed at the beginning of FIB to protect the assembly structure. The shaped cylinder is lifted with an OmniProbe and mounted onto a tungsten needle tip. Scale bar: 1 μm.

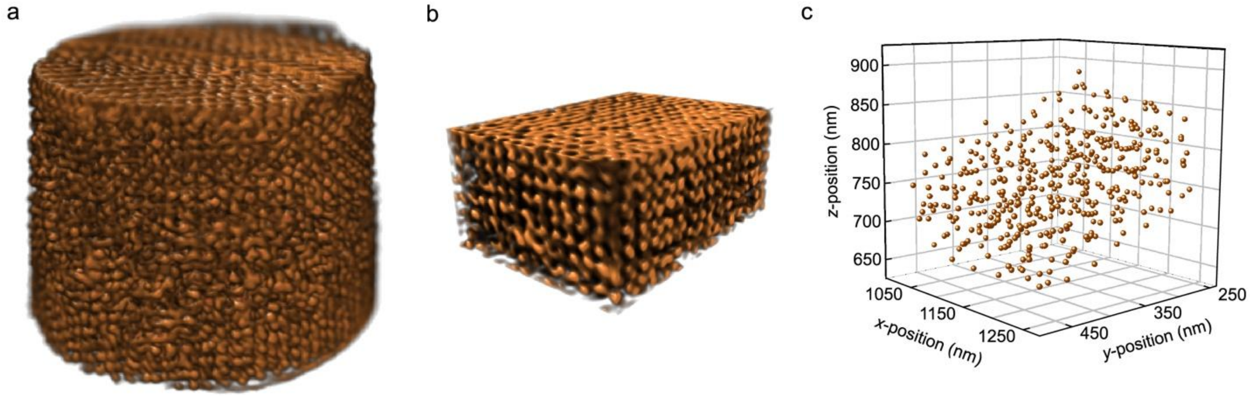

Supplementary Fig. 51. X-ray tomography of patchy small rhombic dodecahedra assembly. (**a**−**c**) The 3D reconstructed tomography of the entire cylindrical shape (**a**), shown in **Supplementary Fig. 50**, a small subsection consisting of 432 NPs (**b**), and the positions of individual particles (**c**) within the subsection shown in (**b**).

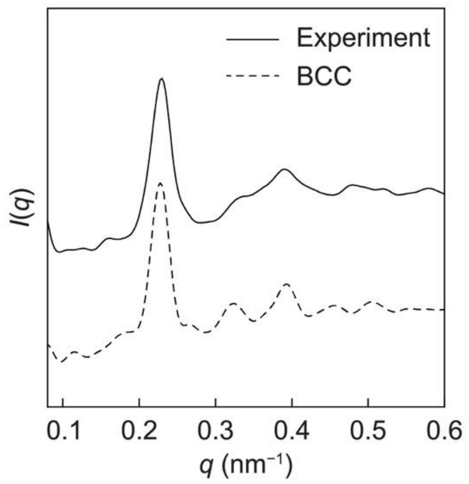

Supplementary Fig. 52. Calculated scattering data from X-ray tomography of patchy small rhombic dodecahedra assembly. The scattering signal calculated from **Supplementary Fig. 51b,c** (solid line) matches a perfect BCC scattering signal from the simulation (dotted lines), confirming that the self-assembly of patchy rhombic dodecahedra forms a BCC lattice. Simulations are performed following a previous work.[31]

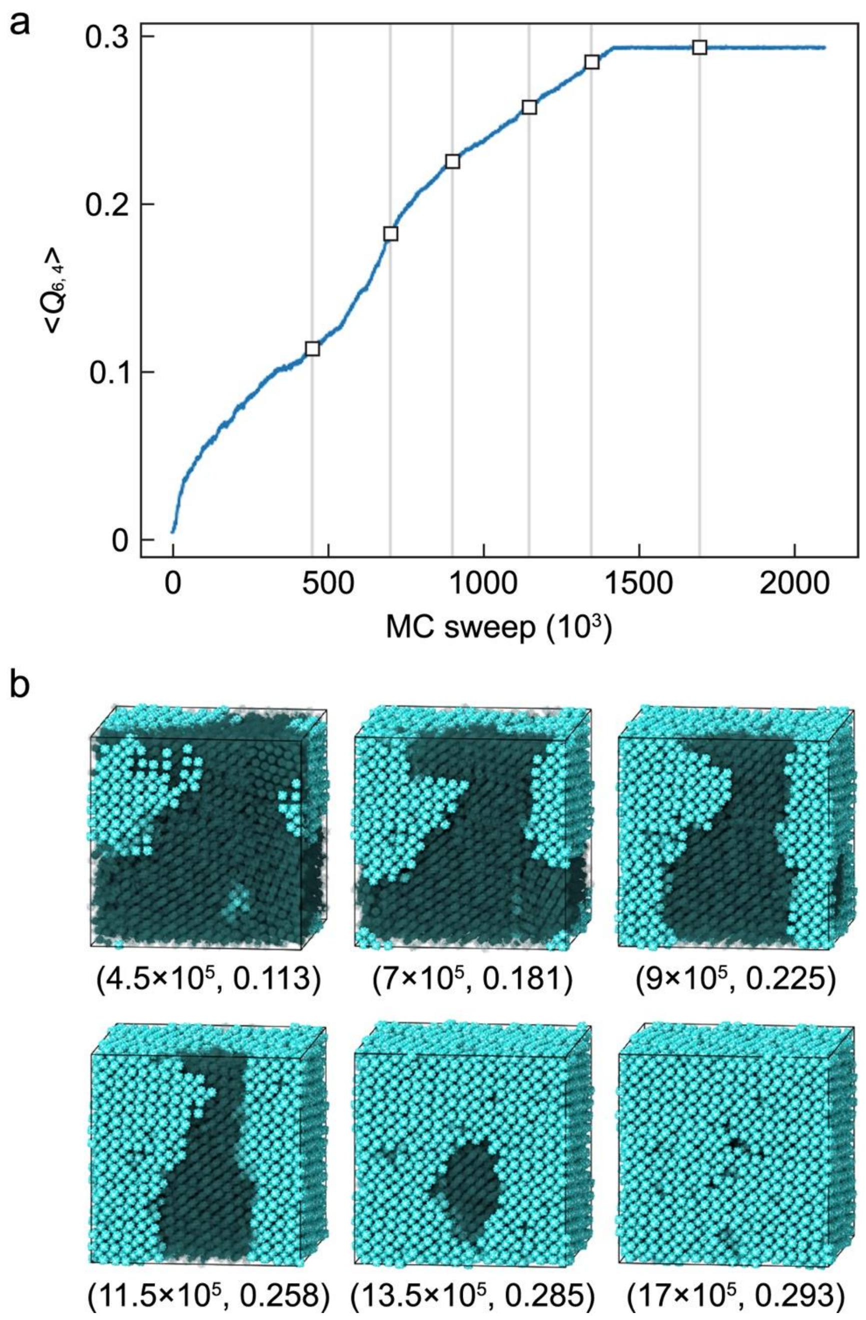

Supplementary Fig. 53. Self-assembly pathway of the patchy rhombic dodecahedra. (**a**) The system-averaged Steinhardt order parameter <$Q_{6,4}$> versus simulation step is plotted. (**b**) The simulated assembly structure corresponding to the marked data in (**a**) is depicted. The system undergoes a steady transition from many grains of differently oriented BCC (dark transparent region) into a single BCC crystal grain (bright cyan region). The caption in (**b**) is (MC sweep number, <$Q_{6,4}$>).

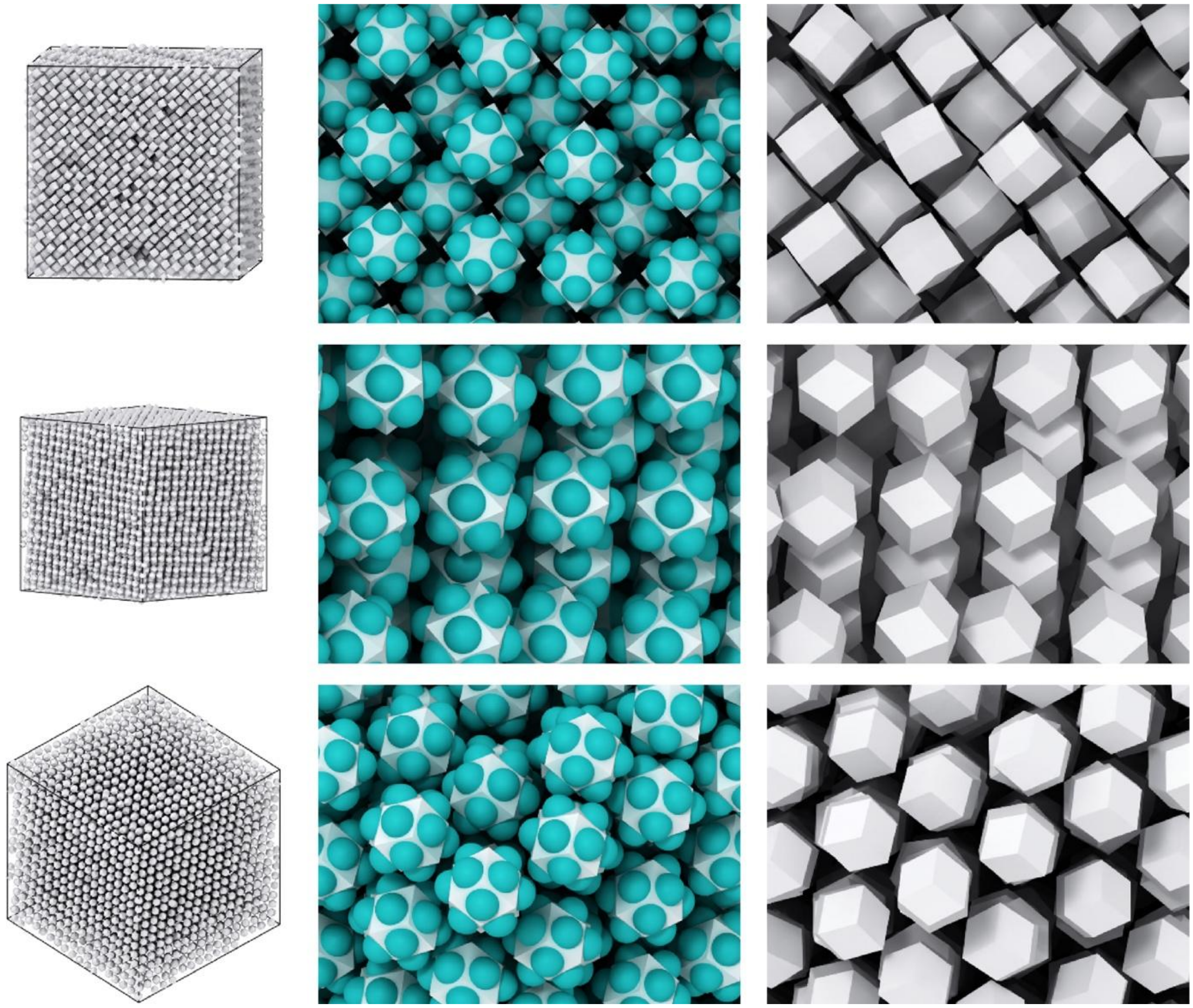

Supplementary Fig. 54. MC simulation for self-assembly of patchy rhombic dodecahedra. Snapshots of the MC simulation for the self-assembly of patchy rhombic dodecahedra, showing the complete (left) and zoomed-in (middle and right) views at different angles. In the right and left columns, patches are omitted for clearer visualization of the lattice structure.

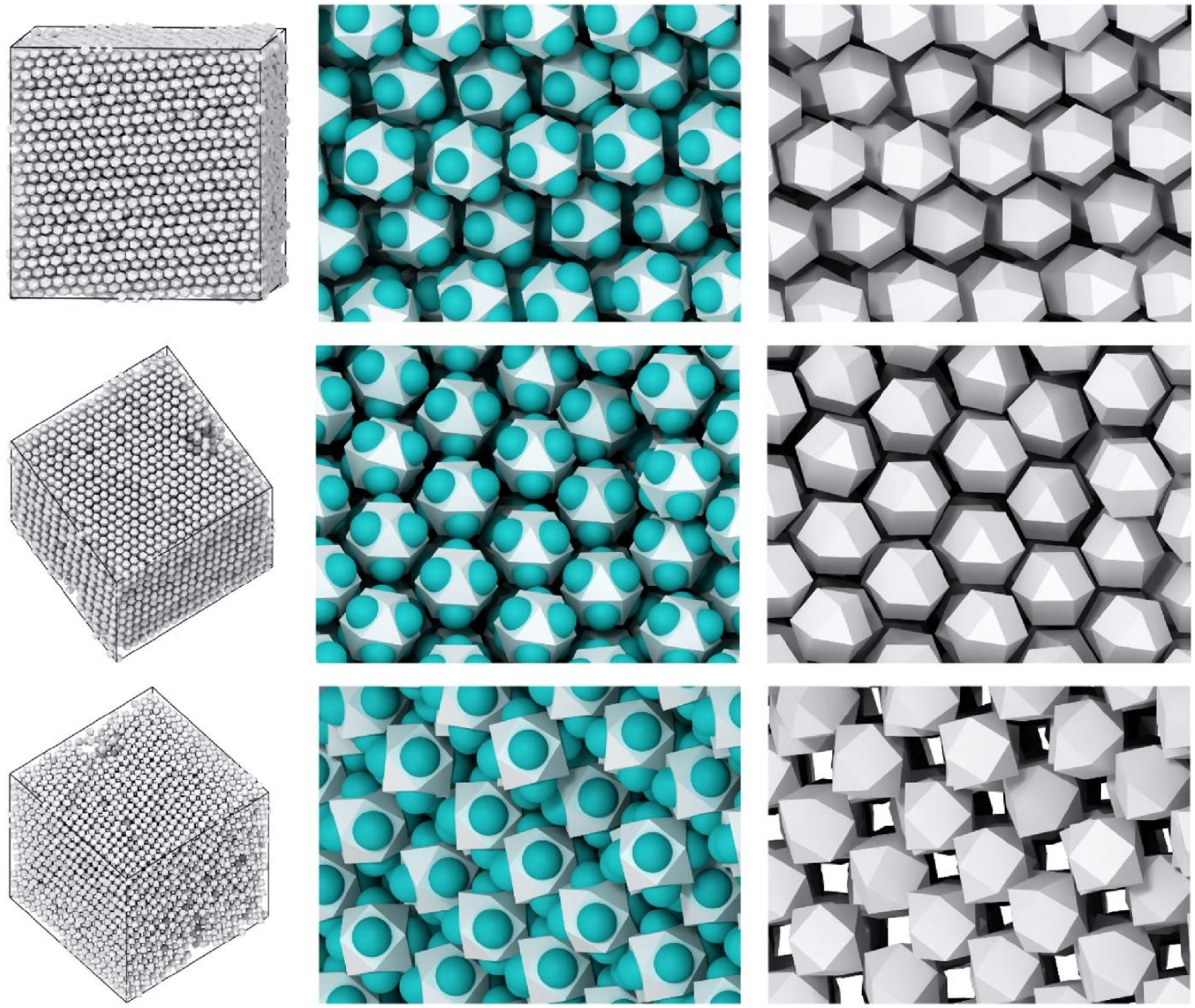

Supplementary Fig. 55. MC simulation for self-assembly of patchy cuboctahedra. Snapshots of the MC simulation for the self-assembly of patchy cuboctahedra, showing the complete (left) and zoomed-in (middle and right) views at different angles. In the right and left columns, patches are omitted for clearer visualization of the lattice structure.

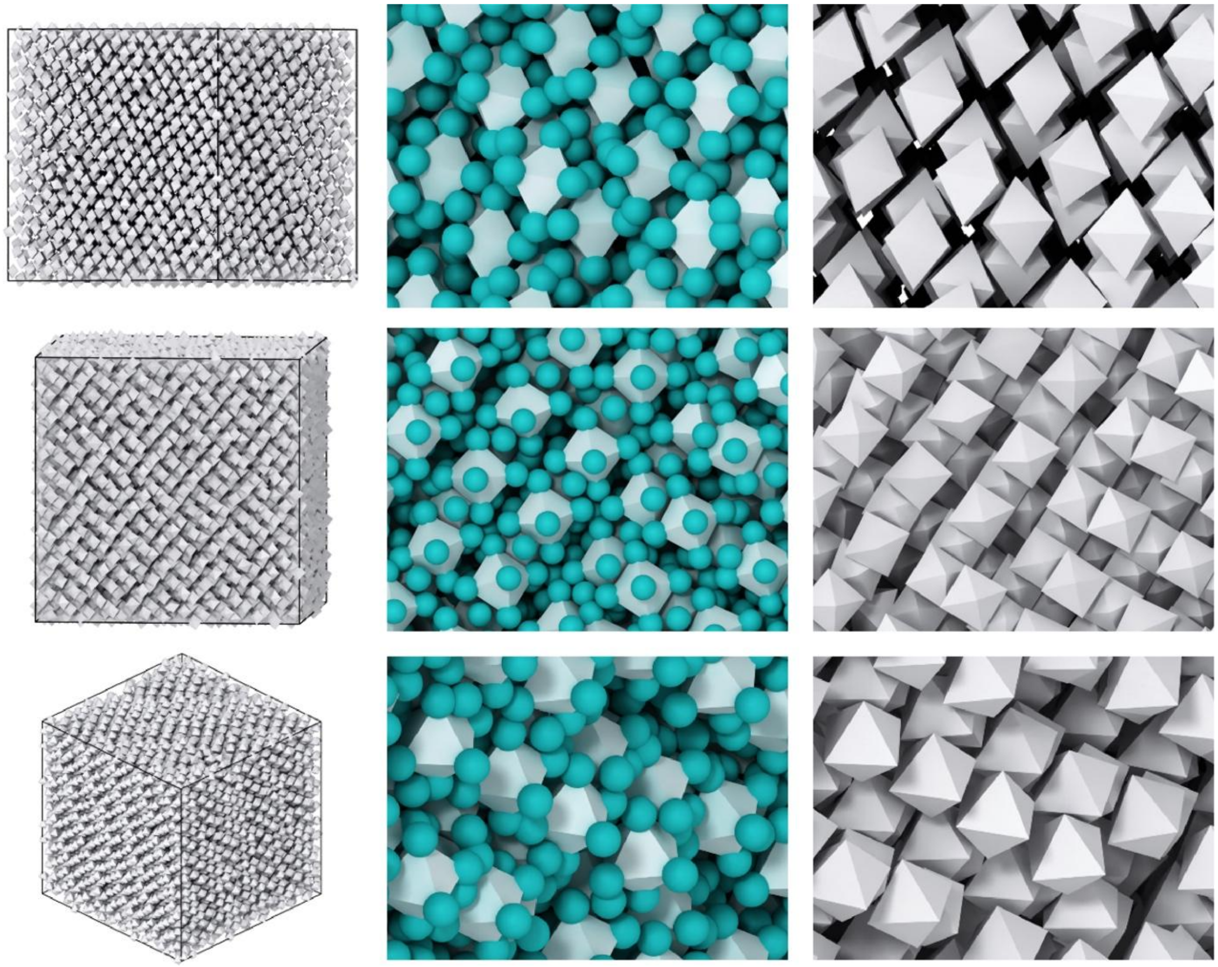

Supplementary Fig. 56. MC simulation for self-assembly of patchy octahedra. Snapshots of the MC simulation for self-assembly of patchy octahedra, showing the complete (left) and zoomed-in (middle and right) views at different angles. In the right and left columns, patches are omitted for clearer visualization of the lattice structure.

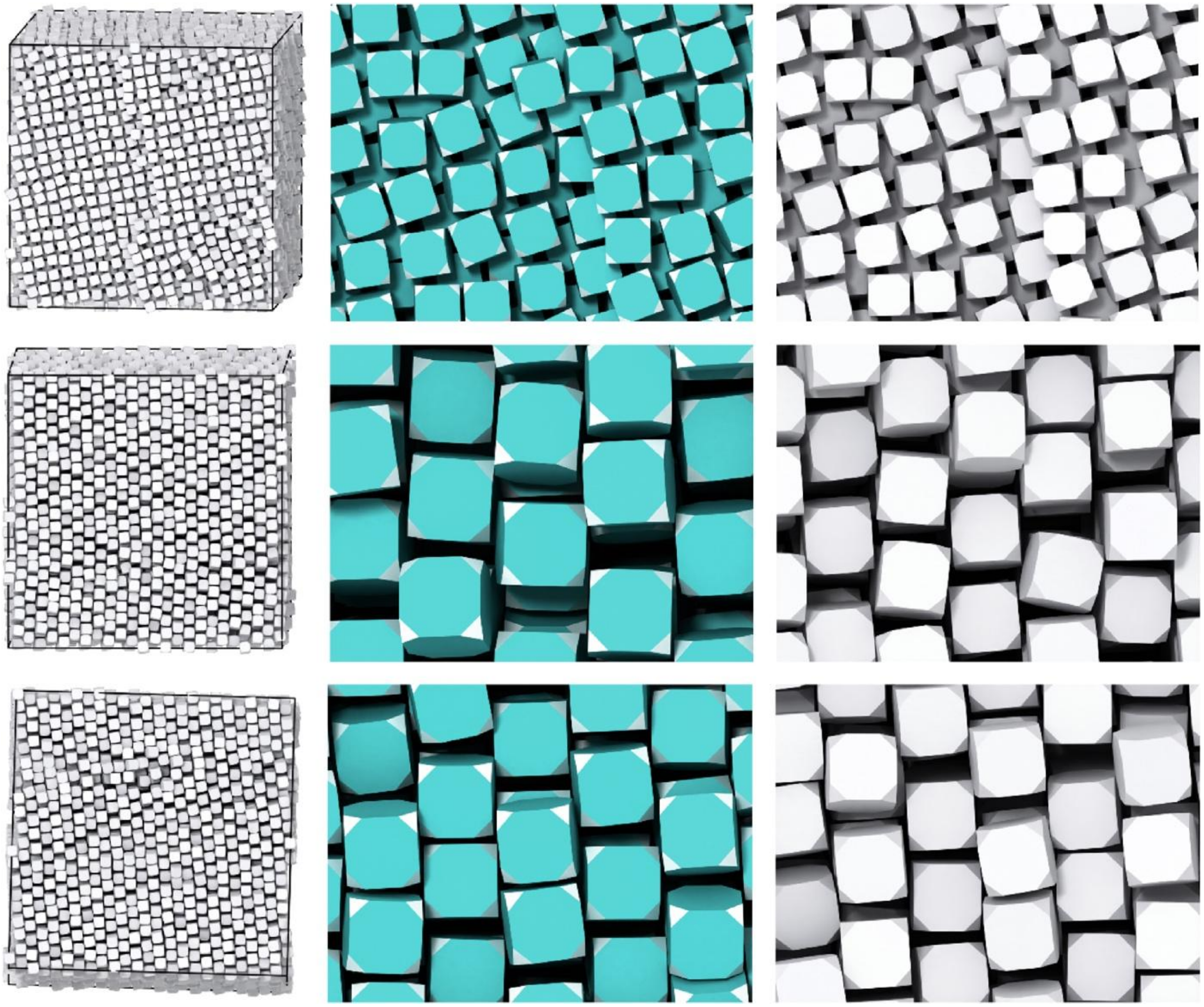

Supplementary Fig. 57. MC simulation for self-assembly of patchy cubes. Snapshots of the MC simulation for the self-assembly of patchy cubes, showing the complete (left) and zoomed-in (middle and right) views at different angles. In the right and left columns, patches are omitted for clearer visualization of the lattice structure.

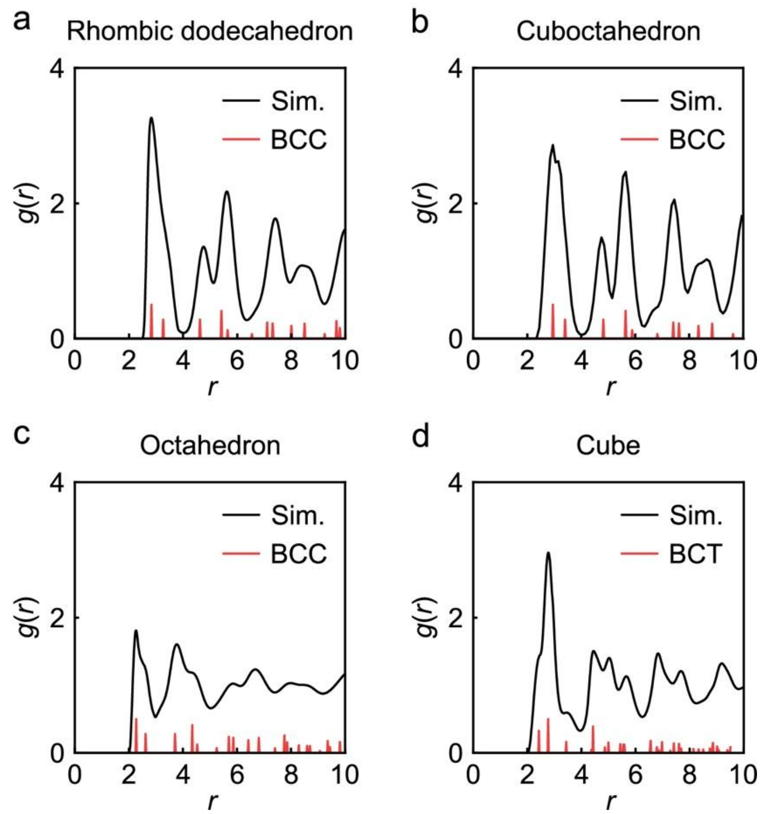

Supplementary Fig. 58. Radial distribution function of simulated patchy NP assemblies. (**a**−**d**) 3D *g(r)*−*r* obtained from the simulated assemblies of patchy rhombic dodecahedra (**a**), cuboctahedra (**b**), octahedra (**c**), and cubes (**d**), showing either BCC or BCT lattices (black), overlaid with those theoretically obtained (red) from perfect BCC or BCT lattices.

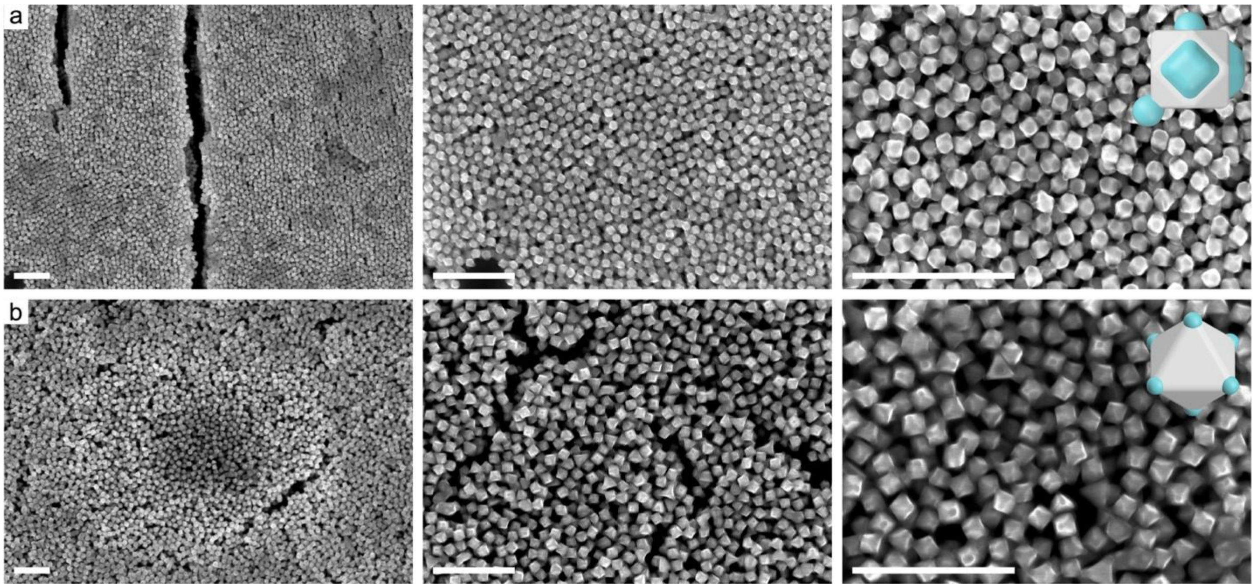

Supplementary Fig. 59. Patch symmetry and size effect on the patchy NP self-assembly. (**a**) SEM images of self-assemblies from asymmetrically patched cuboctahedra featuring hybrid face and vertex patches on the same particle (particles prior to assembly: **Supplementary Fig. 8,** [I^-] = 0.83 μM). Local order is disrupted, likely due to asymmetric patching and reduced electrostatic repulsion from larger exposed gold surfaces. (**b**) SEM images of self-assemblies from octahedra with symmetrically arranged but smaller vertex patches (particles prior to assembly: **Supplementary Fig. 3**, [I^-] = 117.6 μM), which fail to assemble into ordered structures, possibly due to insufficient polymer coverage typically required for long-range ordering (inset: representative schematics of (**a**) symmetry-broken patchy cuboctahedra and (**b**) patchy octahedra with small patches). For detailed synthesis conditions of these patchy NPs, see **Supplementary Tables 2,5**. Scale bars: 500 nm.

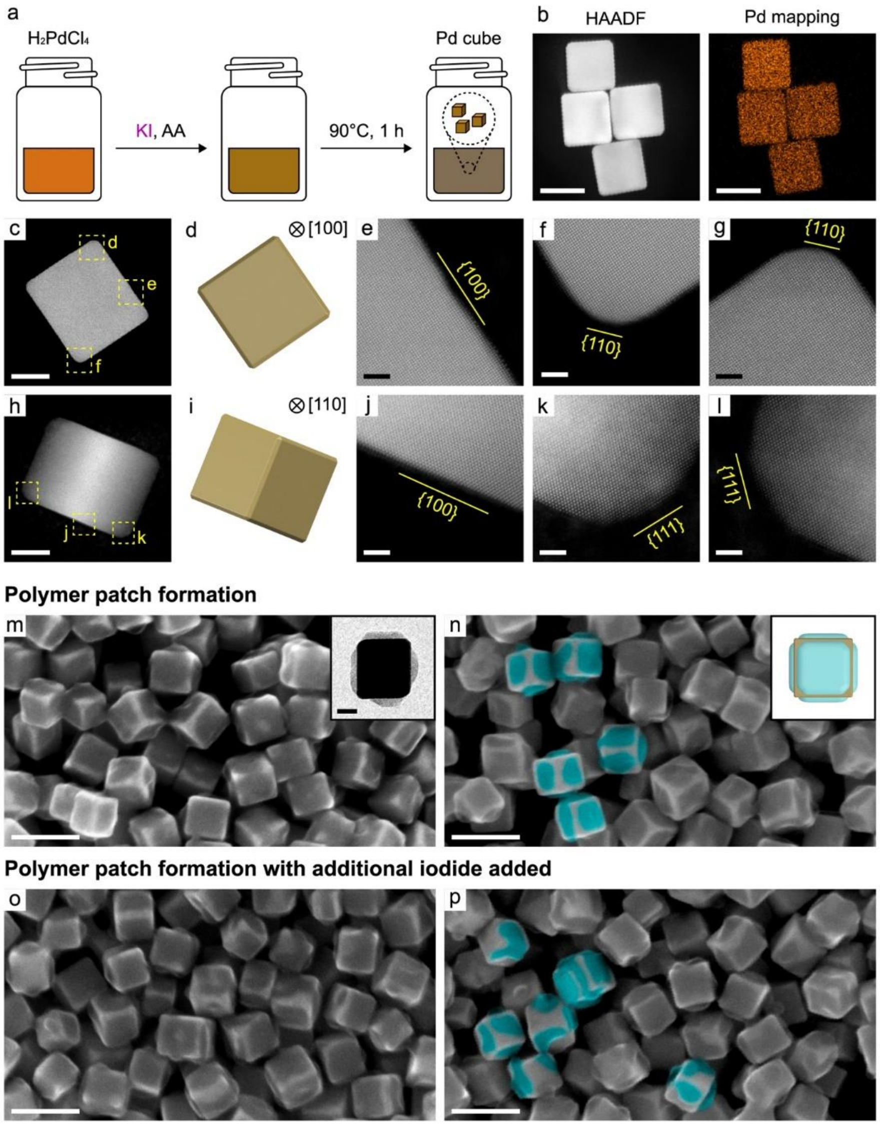

Supplementary Fig. 60. Synthesis of patchy palladium nanocubes. (**a**) Schematic illustrating the synthesis of palladium cubes. Iodides are introduced during the palladium nanocube synthesis, intrinsically enabling selective facet masking for polymer grafting. (**b**) EDX mapping of the palladium cubes showing composition uniformity. (**c–l**) High-angle annular dark field (HAADF)-STEM images of as-synthesized palladium cubes and corresponding 3D schematic. (**d**,**i**) show the viewing direction of the corresponding STEM images. (**e–g**) and (**j–l**) are zoomed-in views of the NP regions boxed in (**c**) and (**h**), showing that the vertices, edges, and faces exhibit {111}, {110}, and {100} facets, respectively, as noted by the yellow lines. (**m**,**n**) Representative SEM images of patchy palladium cubes. Inset: TEM image (**m**) and corresponding 3D schematic (**n**) of a patchy cube with cyan colored region as polymer patches. For these samples, iodides exist as an additive during the palladium cube synthesis process as shown in (**a**). The as-synthesized NPs are used directly for polymer grafting without additional iodide masking step. (**o**,**p**) Representative SEM image of patchy palladium cubes synthesized with the additional iodide masking step performed on the as-synthesized palladium cubes (**o**) and SEM image with selected particles false-colored to highlight the patches (cyan). As more iodide is added, the patch sizes decrease, consistent with the general trend of increased masking at higher [I^-] during stenciling (as seen from **m**,**n** to **o**,**p**). See **Supplementary Note 2.8** for synthesis details. Scale bars: (**b**) 50 nm, (**c**,**h**) 20 nm, (**e–g**, **j–l**) 2 nm, and (**m–p**) 100 nm (inset: 20 nm).

Legends for Supplementary Videos

Supplementary Video 1. 3D reconstruction of patchy polyhedron. Animated 3D rendering and slices of patchy octahedron (left), cube (middle), and bipyramid (right) from TEM tomography.

Supplementary Video 2. 3D reconstruction of patchy cuboctahedron. Animated 3D rendering and slices of representative face-patched (left) and vertex-patched cuboctahedra (right) from TEM tomography.

Supplementary Video 3. 3D reconstruction of patchy rhombic dodecahedra. Animated 3D rendering and slices of patchy rhombic dodecahedra synthesized with 0 (top left), 0.17 (top right), 0.66 (bottom left), and 62.5 μM (bottom right) of [I^-] from TEM tomography.

Supplementary Video 4. Polymer patching dynamics on iodide-masked NPs. MD simulation of polymer grafts on iodide-masking dominated surfaces forming face-patched rhombic dodecahedron (left) and cuboctahedron (right).

Supplementary Video 5. Dynamics of patchy NP self-assemblies. MC simulation of patchy rhombic dodecahedra (top left), cuboctahedra (top right), octahedra (bottom left), and cubes (bottom right) self-assembling into long-range BCC or BCT lattices with distinct interparticle spacings. The core gold NPs and polymer patches are colored white and cyan, respectively.

References

1 Nikoobakht, B. & El-Sayed, M. A. Preparation and growth mechanism of gold nanorods (NRs) using seed-mediated growth method. *Chem. Mater.* **15**, 1957–1962 (2003).

2 O'Brien, M. N., Jones, M. R., Brown, K. A. & Mirkin, C. A. Universal noble metal nanoparticle seeds realized through iterative reductive growth and oxidative dissolution reactions. *J. Am. Chem. Soc.* **136**, 7603–7606 (2014).

3 Rodríguez-Fernández, J., Pérez-Juste, J., Mulvaney, P. & Liz-Marzán, L. M. Spatially-directed oxidation of gold nanoparticles by Au(III)−CTAB complexes. *J. Phys. Chem. B* **109**, 14257–14261 (2005).

4 Lee, J.-H., Gibson, K. J., Chen, G. & Weizmann, Y. Bipyramid-templated synthesis of monodisperse anisotropic gold nanocrystals. *Nat. Commun.* **6**, 7571 (2015).

5 Chen, L. *et al.* High-yield seedless synthesis of triangular gold nanoplates through oxidative etching *Nano Lett.* **14**, 7201–7206 (2014).

6 Chen, L. *et al.* Imaging the kinetics of anisotropic dissolution of bimetallic core–shell nanocubes using graphene liquid cells. *Nat. Commun.* **11**, 3041 (2020).

7 Kim, J. *et al.* Reconfigurable polymer shells on shape-anisotropic gold nanoparticle cores. *Macromol. Rapid Commun.* **39**, 1800101 (2018).

8 Kim, A. *et al.* Tip-patched nanoprisms from formation of ligand islands. *J. Am. Chem. Soc.* **141**, 11796–11800 (2019).

9 Yao, L. *et al.* Seeking regularity from irregularity: unveiling the synthesis–nanomorphology relationships of heterogeneous nanomaterials using unsupervised machine learning. *Nanoscale* **14**, 16479–16489 (2022).

10 Yao, L., Ou, Z., Luo, B., Xu, C. & Chen, Q. Machine learning to reveal nanoparticle dynamics from liquid-phase TEM videos. *ACS Cent. Sci.* **6**, 1421–1430 (2020).

11 Mill, L. *et al.* Synthetic image rendering solves annotation problem in deep learning nanoparticle segmentation. *Small Methods* **5**, 2100223 (2021).

12 Kresse, G. & Hafner, J. Ab initio molecular dynamics for liquid metals. *Phys. Rev. B* **47**, 558–561 (1993).

13 Kresse, G. & Furthmüller, J. Efficient iterative schemes for ab initio total-energy calculations using a plane-wave basis set. *Phys. Rev. B* **54**, 11169–11186 (1996).

14 Kresse, G. & Hafner, J. Ab initio molecular-dynamics simulation of the liquid-metal–amorphous-semiconductor transition in germanium. *Phys. Rev. B* **49**, 14251–14269 (1994).
15 Blöchl, P. E. Projector augmented-wave method. *Phys. Rev. B* **50**, 17953–17979 (1994).
16 Perdew, J. P., Burke, K. & Ernzerhof, M. Generalized gradient approximation made simple. *Phys. Rev. Lett.* **77**, 3865–3868 (1996).
17 Monkhorst, H. J. & Pack, J. D. Special points for Brillouin-zone integrations. *Phys. Rev. B* **13**, 5188–5192 (1976).
18 Grimme, S., Ehrlich, S. & Goerigk, L. Effect of the damping function in dispersion corrected density functional theory. *J. Comput. Chem.* **32**, 1456–1465 (2011).
19 Singh-Miller, N. E. & Marzari, N. Surface energies, work functions, and surface relaxations of low-index metallic surfaces from first principles. *Phys. Rev. B* **80**, 235407 (2009).
20 Pašti, I. A. & Mentus, S. V. Halogen adsorption on crystallographic (1 1 1) planes of Pt, Pd, Cu and Au, and on Pd-monolayer catalyst surfaces: first-principles study. *Electrochim. Acta* **55**, 1995–2003 (2010).
21 Reckien, W., Janetzko, F., Peintinger, M. F. & Bredow, T. Implementation of empirical dispersion corrections to density functional theory for periodic systems. *J. Comupt. Chem.* **33**, 2023–2031 (2012).
22 Pereira, A. O. & Miranda, C. R. Atomic scale insights into ethanol oxidation on Pt, Pd and Au metallic nanofilms: a DFT with van der Waals interactions. *Appl. Surf. Sci.* **288**, 564–571 (2014).
23 Kim, A. *et al.* Symmetry-breaking in patch formation on triangular gold nanoparticles by asymmetric polymer grafting. *Nat. Commun.* **13**, 6774 (2022).
24 Haario, H., Saksman, E. & Tamminen, J. An adaptive Metropolis algorithm. *Bernoulli* **7**, 223–242 (2001).
25 Ramasubramani, V., Vo, T., Anderson, J. A. & Glotzer, S. C. A mean-field approach to simulating anisotropic particles. *J. Chem. Phys.* **153**, 084106 (2020).
26 Anderson, J. A., Glaser, J. & Glotzer, S. C. HOOMD-blue: a Python package for high-performance molecular dynamics and hard particle Monte Carlo simulations. *Comput. Mater. Sci.* **173**, 109363 (2020).
27 Gong, J. *et al.* Shape-dependent ordering of gold nanocrystals into large-scale superlattices. *Nat. Commun.* **8**, 14038 (2017).
28 Henzie, J., Grünwald, M., Widmer-Cooper, A., Geissler, P. L. & Yang, P. Self-assembly of uniform polyhedral silver nanocrystals into densest packings and exotic superlattices. *Nat. Mater.* **11**, 131–137 (2012).
29 Luo, B. *et al.* Unravelling crystal growth of nanoparticles. *Nat. Nanotechnol.* **18**, 589–595 (2023).
30 Niu, W. *et al.* Selective synthesis of single-crystalline rhombic dodecahedral, octahedral, and cubic gold nanocrystals. *J. Am. Chem. Soc.* **131**, 697–703 (2009).
31 Yager, K. G., Zhang, Y., Lu, F. & Gang, O. Periodic lattices of arbitrary nano-objects: modeling and applications for self-assembled systems. *J. Appl. Cryst.* **47**, 118–129 (2014).
